# Evolutionary and functional divergence of MYB regulatory networks in flavonoid biosynthesis across Poaceae, non-Poaceae monocots, and eudicot horticultural plants

**DOI:** 10.1093/hr/uhag169

**Published:** 2026-04-27

**Authors:** Yao Gong, Xiangjing Chen, Zeyuan Qian, Pinyu Pan, Zhichao Sun, Zekang Pan, Zhengjia Wang, Xiang Gao, Shanyue Jiang, Liangsheng Zhang

**Affiliations:** Zhejiang Key Laboratory of Horticultural Crop Quality Improvement, College of Agriculture and Biotechnology, Zhejiang University, Hangzhou, China; Zhejiang Key Laboratory of Horticultural Crop Quality Improvement, College of Agriculture and Biotechnology, Zhejiang University, Hangzhou, China; Zhejiang Key Laboratory of Horticultural Crop Quality Improvement, College of Agriculture and Biotechnology, Zhejiang University, Hangzhou, China; Zhejiang Key Laboratory of Horticultural Crop Quality Improvement, College of Agriculture and Biotechnology, Zhejiang University, Hangzhou, China; National Key Laboratory for Development and Utilization of Forest Food Resources, Zhejiang A&F University, Hangzhou, China; Yazhouwan National Laboratory, Sanya, Hainan, China; National Key Laboratory for Development and Utilization of Forest Food Resources, Zhejiang A&F University, Hangzhou, China; Key Laboratory of Molecular Epigenetics of MOE and Institute of Genetics & Cytology, Northeast Normal University, Changchun, China; Zhejiang Key Laboratory of Horticultural Crop Quality Improvement, College of Agriculture and Biotechnology, Zhejiang University, Hangzhou, China; Zhejiang Key Laboratory of Horticultural Crop Quality Improvement, College of Agriculture and Biotechnology, Zhejiang University, Hangzhou, China; Yazhouwan National Laboratory, Sanya, Hainan, China

## Abstract

Horticultural plants synthesize diverse flavonoids (e.g. anthocyanins, proanthocyanidins, and flavonols) that shape key agronomic traits, including flower pigmentation, fruit flavor, stress resilience, and nutritional value. MYB transcription factors (TFs), particularly R2R3-MYBs, serve as core regulators of flavonoid biosynthesis; their functional divergence and distinct regulatory mechanisms drive the interspecific diversity of flavonoid-related traits. This review systematically summarizes the evolutionary and functional divergence of flavonoid-regulatory R2R3-MYBs across monocot (including Poaceae and non-Poaceae) and eudicot horticultural plants. Phylogenetic analysis, integrating functionally validated R2R3-MYB sequences and *Arabidopsis* references, classifies the R2R3-MYB family into 37 subgroups. Flavonoid-regulatory members primarily cluster within key dedicated subgroups, which are first categorized into six functional groups and subsequently subdivided into 11 distinct subclades based on their monophyletic distribution in angiosperms across 44 horticultural species. Distinct lineage-specific evolutionary patterns emerge: some monocots (notably Poaceae) putatively lack Subgroup 6 (SG6) and rely on SG5 for anthocyanin biosynthesis, while eudicots expand SG6 via tandem duplications. Rosaceae exhibit adaptive expansion of SG5 associated with fruit trait evolution. We further discuss cross-species divergence within conserved flavonoid-regulatory subgroups, including variations in spatiotemporal expression patterns and dependency on the MYB–bHLH–WD40 (MBW) complex. Case studies of representative species (*Arabidopsis*, rice, strawberry, and freesia) demonstrate that paralogs derived from gene duplication achieve precise regulation of distinct flavonoid branch pathways through subfunctionalization or neofunctionalization. Ecological pressures, domestication, and structural determinants collectively drive MYB divergence in copy number and function. Finally, we outline the applications of these findings in crop improvement and propose future research directions.

## Introduction

Horticultural plants, domesticated over millennia, are indispensable to human society, serving critical roles in ensuring food security, enhancing aesthetic landscapes, and providing valuable medicinal resources. To cope with dynamic environments and human demands, these plants have developed sophisticated adaptive strategies [1]. A key component of this adaptation lies in the biosynthesis of secondary metabolites, particularly in organs such as flowers, fruits, and leaves, which mediate interactions with both the environment and other organisms [2]. Among these metabolites, flavonoids, a large and diverse class of phenylpropanoid compounds, stand out as pivotal determinants of horticultural traits. They shape flower pigmentation that attracts pollinators, modulate fruit flavor and astringency that influence consumer preference, enhance stress resistance that safeguards crop productivity, and contribute to nutritional value that benefits human health [3]. Given their profound impact on crop quality and economic worth, flavonoid-related traits have long been core targets of domestication and modern breeding efforts.

**Figure 1:**
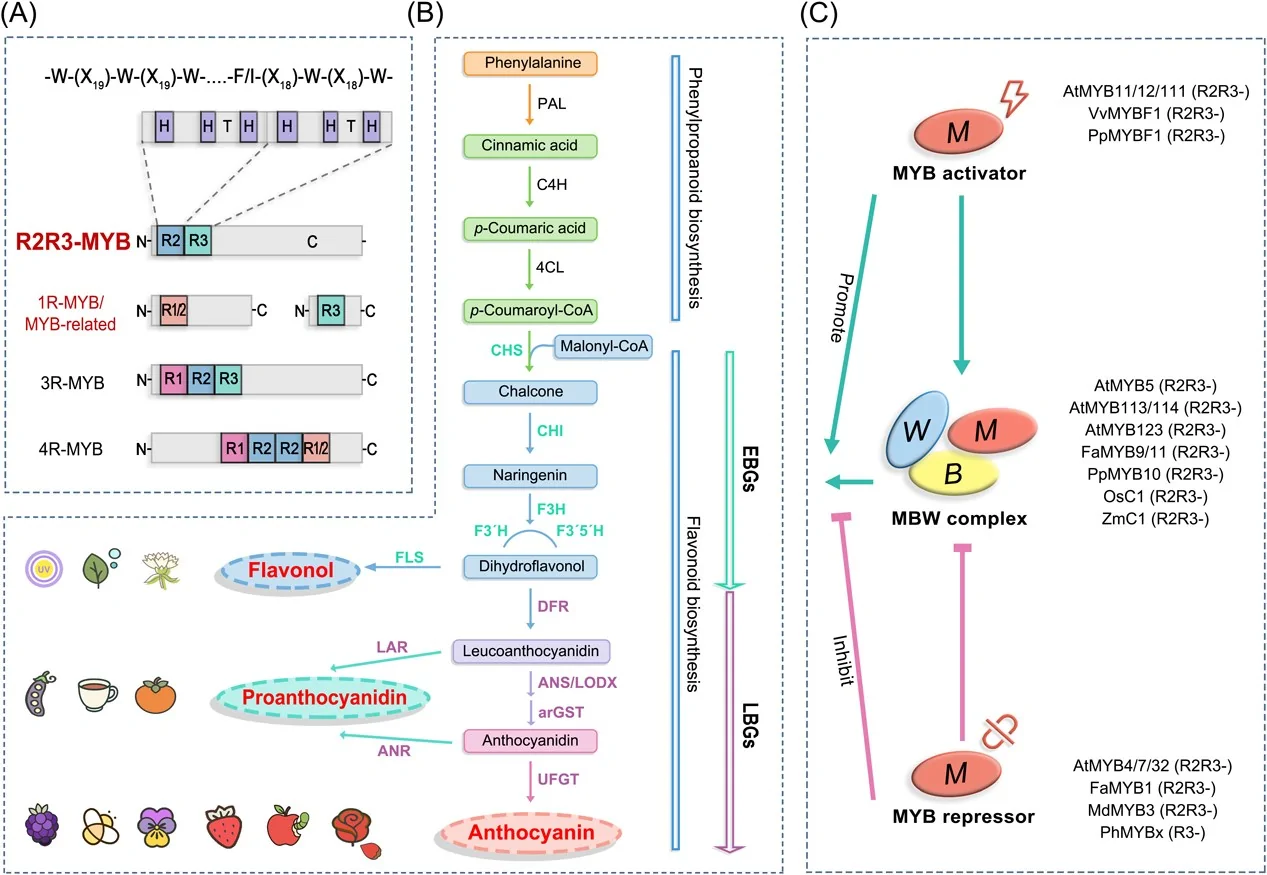
Classification of MYB TFs and a simplified model illustrating their functional roles in the general flavonoid biosynthesis pathway. (A) Structural classification of MYB TFs based on the number of MYB domains, including 1R, 2R/R2R3, 3R, and 4R groups. The primary and secondary structures of a typical R2R3-MYB are shown, with H (helix), T (turn), W (tryptophan), and X (any amino acid) labeled. (B) Schematic representation of the flavonoid biosynthesis pathway leading to anthocyanins, proanthocyanidins, and flavonols. This panel also depicts the distribution and biological functions of anthocyanins, proanthocyanidins, and flavonols in representative horticultural plants. (C) Regulatory roles of MYB TFs and MBW complexes in promoting or inhibiting flavonoid biosynthesis.

MYB transcription factors (TFs), one of the largest and most versatile TF families in plants, are central regulators of flavonoid biosynthesis [4]. Structurally, MYB proteins are defined by two functionally distinct domains: a highly conserved N-terminal MYB domain responsible for sequence-specific DNA binding, and a variable C-terminal region that drives functional diversity by mediating protein–protein interactions or acting as transcriptional activation/repression modules [5, 6]. The MYB domain comprises one to four imperfect repeating units (designated R), each consisting of ~50–53 amino acids that fold into a helix–turn–helix structure critical for DNA recognition [7]. Based on the number of R repeats, MYBs are classified into four major types: MYB-related proteins (containing R1/2 or R3 repeats), R2R3-MYBs (the most abundant type in plants), 3R-MYBs (with R1R2R3 repeats), and 4R-MYBs (Fig. 1A) [8]. R2R3-MYBs dominate flavonoid regulation, functioning as either activators or repressors by binding to cis-elements in the promoters of flavonoid biosynthetic genes [9–12]. In contrast, R3-MYBs typically act as negative regulators, attenuating flavonoid metabolism through mechanisms, such as competing with R2R3-MYBs for binding partners (Fig. 1B and C) [11, 13]. The pivotal role of MYBs in flavonoid biosynthesis was first uncovered with the identification of *COLORED1 (C1)* in *Zea mays*, the first plant R2R3-MYB shown to regulate anthocyanin production in aleurone tissues [14]. Since then, numerous *MYB* genes involved in flavonoid pathways have been characterized across a wide range of horticultural species. While previous classifications of flavonoid-regulatory R2R3–MYB subfamilies have primarily relied on sequence homology and conserved motifs, these approaches often yield overly generalized groupings that lack sufficient resolution for functional specialization. For instance, Subgroup 5 (SG5) is typically associated with proanthocyanidin biosynthesis activation, Subgroup 6 (SG6) with anthocyanin pathway promotion, Subgroup 7 (SG7) with flavonol production enhancement, and Subgroup 4 (SG4) with general flavonoid gene repression [4, 8, 15, 16]. Flavonoid biosynthesis, especially for downstream branch pathways (e.g. anthocyanins and proanthocyanidins), is usually governed by the MYB–bHLH–WD40 (MBW) complex, which consists of MYB, basic helix–loop–helix (bHLH), and WD40-repeat proteins [17]. This conserved complex in higher plants binds target gene promoters to regulate late-stage flavonoid biosynthetic steps, with MYBs serving as the primary determinants of tissue specificity and pathway specificity. Beyond direct regulation via the MBW complex, MYBs can also indirectly modulate flavonoid production by influencing the assembly, stability, or activity of this complex (Fig. 1B and C) [18, 19]. Importantly, TFs in the flavonoid pathway evolve more rapidly than structural genes, and MYBs have been shown to drive pigmentation variation to a greater extent than their bHLH or WD40 counterparts [20, 21].

Horticultural plants are predominantly angiosperms, with monocots and eudicots diverging ~140 million years ago during the Early Cretaceous. This ancient split gave rise to distinct morphological, physiological, and genetic profiles between these two lineages [22] and shaped their different pollination strategies—eudicots (e.g. Rosaceae, Asteraceae) typically rely on insect-mediated pollination, while monocots (e.g. Poaceae) are mainly wind-pollinated. During the long-term adaptive evolution following this divergence, evolutionary shifts occurred in the regulatory networks of MYB TFs, a process tightly linked to the functional diversification of the MYB gene family. This evolutionary shift in MYB regulatory networks is considered a key mechanism driving the adaptive divergence in flavonoid biosynthesis and accumulation patterns between monocots and eudicots. Consequently, such divergence in MYB-mediated flavonoid regulation further leads to phenotypic differences between the two lineages, which is prominently reflected in major horticultural families: core eudicot families including Rosaceae (strawberry, apple, rose) and Solanaceae (tomato, potato, petunia), as well as monocot families, such as Poaceae (maize, rice, wheat), Musaceae (banana), and Orchidaceae (orchids). Thus, clarifying the evolutionary trajectories of MYB TFs within and across these major horticultural families provides a critical framework for deciphering the molecular basis of flavonoid-related trait diversity in domesticated crops.

This review synthesizes current knowledge on the evolutionary and functional divergence of flavonoid-regulatory R2R3–MYBs across Poaceae, non-Poaceae monocots, and eudicot horticultural plants. Specifically, we aim to: (i) briefly introduce the diversity of flavonoids and their biological roles in horticultural crops, with a focus on the core biosynthesis pathway; (ii) dissect divergence within key flavonoid-regulatory subgroups by subdividing the previously defined Subgroups 4, 5, 6, and 7 [8] into six functional groups for refined resolution: SG6 (primarily anthocyanin activators), SG5 (primarily proanthocyanidin/anthocyanin activators), SG7 (primarily flavonol activators), SGAtM5 (primarily general flavonoid activators), SG4 (primarily flavonoid repressors), and SG15 (primarily trichome regulators). These groups are further classified into 11 monophyletic subclades to explore their ancestral origins, lineage-specific gene loss/expansion patterns, and functional specialization; (iii) explore cross-species divergence within conserved flavonoid-regulatory subgroups, with an emphasis on anthocyanin-regulatory clades (notably SG6 and SG5), by analyzing variations in spatiotemporal expression patterns, MBW complex dependency, and regulation of non-anthocyanin flavonoid classes; (iv) illustrate intra-species divergence following gene duplication through case studies in representative species, highlighting how paralogs undergo subfunctionalization or neofunctionalization to refine regulatory precision; (v) elucidate the evolutionary drivers (ecological adaptation, domestication) and molecular mechanisms (structural motifs) underlying these divergences; and (vi) propose strategies to translate these insights into crop improvement and identify priority areas for future research. By integrating evolutionary perspectives with molecular mechanisms, this review provides a comprehensive framework to advance our understanding of flavonoid regulation, facilitating the precise manipulation of flavonoid profiles to enhance key horticultural traits.

## Diversity of flavonoid biosynthesis pathways and their roles in horticultural plants

Flavonoids, a large, structurally diverse family of phenylpropanoid secondary metabolites, are defined by a conserved C6–C3–C6 carbon backbone (two aromatic rings linked by a three-carbon heterocyclic ring) [23–26]. Over 10 000 distinct flavonoid compounds have been identified in horticultural plants to date, with key subclasses including anthocyanins, proanthocyanidins, flavonols, flavones, isoflavones, dihydroflavonols, and phlobaphenes—each with unique chemical properties that underpin their functional roles in horticultural traits [24, 27]. Notably, some flavonoids exhibit strict species specificity: isoflavones are predominantly synthesized in leguminous crops such as soybean (*Glycine max*) and common bean (*Phaseolus vulgaris*), while phlobaphenes are primarily found in Poaceae species like maize [28, 29]. Among these subclasses, anthocyanins are the major contributors to plant pigmentation, with color variation primarily determined by B-ring hydroxylation and O-methylation: the number of B-ring hydroxyl groups (–OH) directly shapes hue (more hydroxyls shift colors toward blue/violet), while methylation replaces –OH with –OCH₃ to reduce molecular polarity, causing a slight reddening [27, 30].

The core flavonoid biosynthesis pathway, well-characterized in horticultural plants, features key branch points that direct metabolic flux toward anthocyanins, proanthocyanidins, or flavonols (Fig. 1B). Phenylalanine is converted to *p*-coumaroyl-CoA via phenylalanine ammonia lyase (*PAL*), followed by cinnamate 4-hydroxylase (*C4H*), and 4-coumaroyl-CoA ligase (*4CL*) in the general phenylpropanoid pathway to generate the common substrate of flavonoids [31]. Chalcone synthase (*CHS*) catalyzes the first committed step, producing chalcone from *p*-coumaroyl-CoA, and then chalcone is converted into naringenin by chalcone isomerase (*CHI*). Naringenin is subsequently hydroxylated by flavanone 3-hydroxylase (*F3H*), flavonoid 3′-hydroxylase (*F3*′*H*), or flavonoid 3′5′-hydroxylase (*F3*′*5*′*H*) to form dihydroflavonols [3]. These enzymes (*CHS*, *CHI*, *F3H*, *F3*′*H*, *F3*′*5*′*H*) mediate the initial steps and their encoding genes are considered as early biosynthetic genes (EBGs). Dihydroflavonols are then processed by flavonol synthase (*FLS*) into flavonols or by dihydroflavonol 4-reductase (*DFR*) into leucoanthocyanidins, leading to anthocyanins and proanthocyanidins [32]. *DFR*, leucoanthocyanidin reductase (*LAR*), anthocyanidin reductase (*ANR*), anthocyanidin synthase (*ANS*), anthocyanin-related glutathione S-transferase (*arGST*) [33], and UDP-glucose:flavonoid 3-O-glucosyltransferase (*UFGT*) are categorized as late biosynthetic genes (LBGs). It should be noted that EBG/LBG classification remains controversial. *FLS* is traditionally categorized as an EBG, but its terminal product (flavonols) competes with anthocyanin/proanthocyanidin pathways for dihydroflavonol precursors. Pathway transcriptional crosstalk suggests this conventional framework may oversimplify *FLS*-related regulatory complexity. Key anthocyanin biosynthetic and modification enzymes (*F3*′*H*, *F3*′*5*′*H*, *arGST*, *UFGT*, etc.) are directly or indirectly regulated by MYBs. Specifically, *F3*′*H* and *F3*′*5*′*H* determine B-ring hydroxylation, flower color, and anthocyanin stability; *arGST* functions as a catalytic dehydratase essential for the penultimate step of anthocyanin biosynthesis, converting the unstable leucoanthocyanidin dioxygenase (*LDOX)* product into cyanidin; and *UFGT* catalyzes glucose transfer to anthocyanidins, forming anthocyanin-3-O-glucosides (essential for stabilization and vacuolar storage). Additionally, vacuolar transporters for anthocyanin sequestration are complexly regulated, with MYBs potentially influencing their expression directly or indirectly. The divergence between anthocyanin and proanthocyanidin biosynthesis occurs post-leucoanthocyanidin formation (catalyzed by *DFR*, a shared precursor step), with the control point mediated by functional divergence of key enzymes and MYB transcriptional regulation. Specifically, *ANS* is predominantly dedicated to anthocyanin biosynthesis (*ans* mutants show >90% reduced anthocyanin accumulation) and contributes to proanthocyanidin extension unit generation, while *LDOX* functions exclusively in the proanthocyanidin pathway by coupling with *ANR*, preventing flux toward anthocyanins.

Flavonoids play multiple pivotal roles in horticultural plants, spanning plant adaptation and agronomic value. Flavonoids drive flower and fruit pigmentation, influencing hue, saturation, and patterning through structural diversity, accumulation, and interactions with cellular microenvironments and other pigments. Anthocyanins, derived from six widespread anthocyanin aglycons (i.e. pelargonidin, cyanidin, peonidin, delphinidin, petunidin, and malvidin), are employed as primary pigments conferring red, orange, purple, and blue hues in flowers, leaves, and fruits to attract pollinators, seed dispersers, and to respond to environmental changes (Fig. 1B) [32]. Flavonols, often colorless or pale yellow, act as co-pigments, enhancing petal gloss or transparency, as seen in ivory or cream hues of lily or carnation flowers. In ornamental crops, floral color diversity boosts market value, while uniform fruit pigmentation signals ripeness and commercial quality. MYB networks integrate environmental signals (light, temperature, hormones) to trigger flavonoid production [34]. Flavonoids in the epidermis act as UV-B ‘sunscreens’, protecting photosynthetic tissues, especially in high-light conditions [35]. As reactive oxygen species (ROS) scavengers, they mitigate oxidative stress from drought, salinity, and extreme temperatures, aiding cellular homeostasis [34]. Flavonoids may also support osmotic adjustment under water stress [36]. Anthocyanins and derivatives provide antimicrobial defense against fungi and bacteria, while vivid colors and proanthocyanidin-rich tissues deter herbivores through aposematic signaling or astringency [37, 38]. MYBs regulate balance between proanthocyanidins and anthocyanins, affecting astringency and sweetness. During ripening, anthocyanin accumulation enhances perceived sweetness, while proanthocyanidin degradation reduces bitterness; imbalances yield astringent (e.g. unripe persimmons) or bland fruits [39, 40]. MYBs and enzymes like *PAL* co-regulate anthocyanins and volatile phenolics or terpenes, linking pigmentation to aroma profiles [41]. Flavonoids, especially anthocyanins, are potent antioxidants linked to anti-inflammatory, cardiovascular, neuroprotective, and potential anticancer benefits, as well as metabolic regulation [42, 43]. Anthocyanin-rich crops (e.g. blueberry, purple grape) are valued as nutraceuticals, with color indicating nutritional quality.

## Evolutionary origins and trajectories of key flavonoid-regulatory subgroups

**Figure 2:**
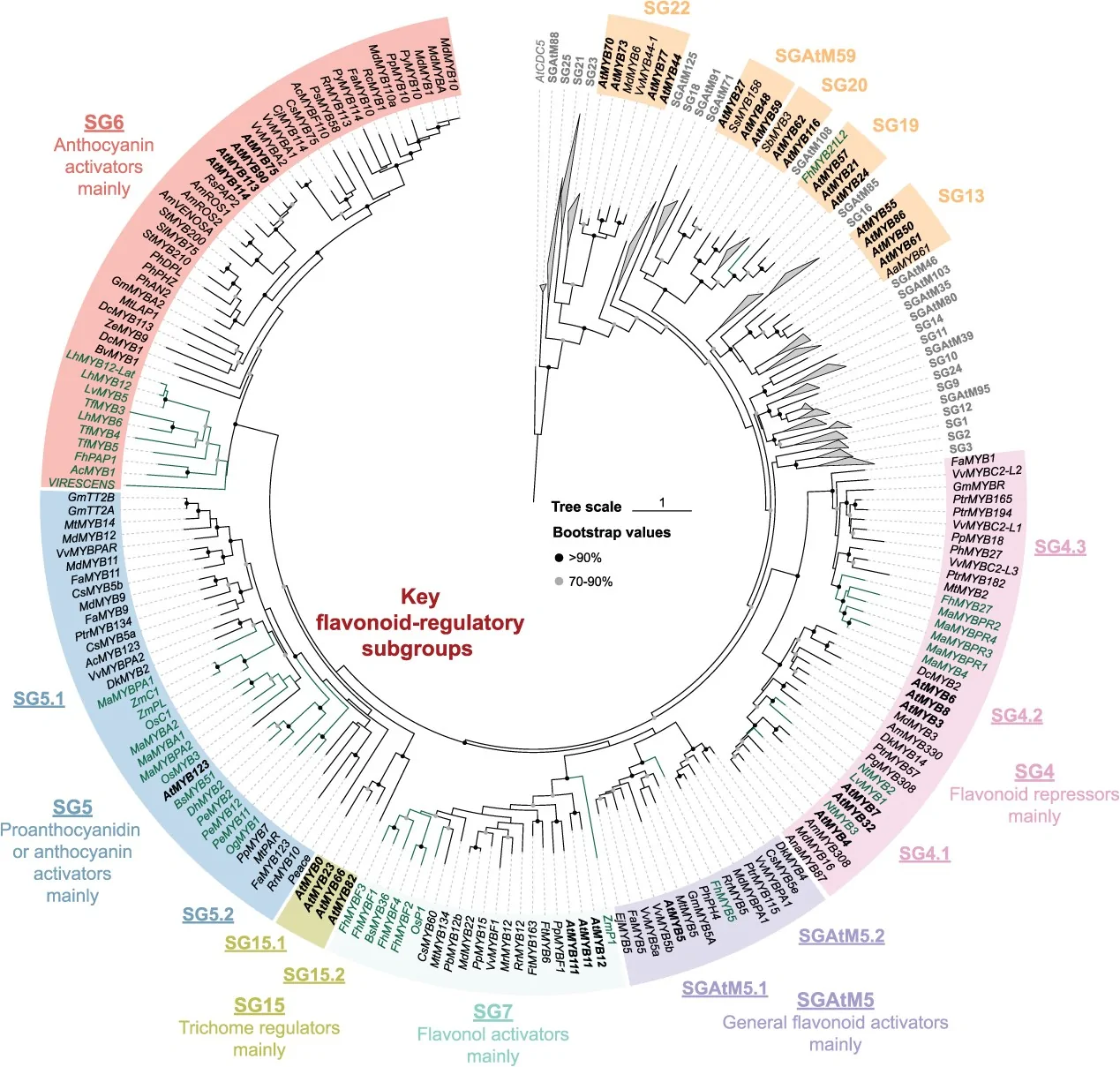
Rooted phylogenetic tree of R2R3–MYB genes from *Arabidopsis* and functionally characterized flavonoid-related R2R3–MYBs in horticultural plants. The tree includes 270 nonredundant R2R3–MYB sequences: 126 from *Arabidopsis* [7] and 143 functionally validated ones from other horticultural plants. AtCDC5 was used as the outgroup for rooting. R2R3–MYBs from monocots are highlighted in dark green, and *Arabidopsis* R2R3–MYBs are bolded for clarity. Subgroup classification followed the frameworks of *Arabidopsis* [8], dividing all candidate R2R3–MYBs into 37 subgroups. Only subgroups involved in flavonoid regulation are displayed; phylogenetic clades of other subgroups are collapsed. Key flavonoid-regulatory subgroups are categorized into six functional groups: SG6 (anthocyanin activators mainly), SG5 (proanthocyanidin or anthocyanin activators mainly), SG15 (trichome regulators mainly), SG7 (flavonol activators mainly), SGAtM5 (general flavonoid activators mainly), and SG4 (flavonoid repressors mainly). Based on phylogenetic trees constructed using orthogroup members of each subgroup across 44 horticultural species (integrated from Fig. 3 and Supplementary Fig. S3), SG5 was subdivided into SG5.1 and SG5.2, SGAtM5 into SGAtM5.1 and SGAtM5.2, SG4 into SG4.1, SG4.2, and SG4.3, and SG15 into SG15.1 and SG15.2.

**Figure 3:**
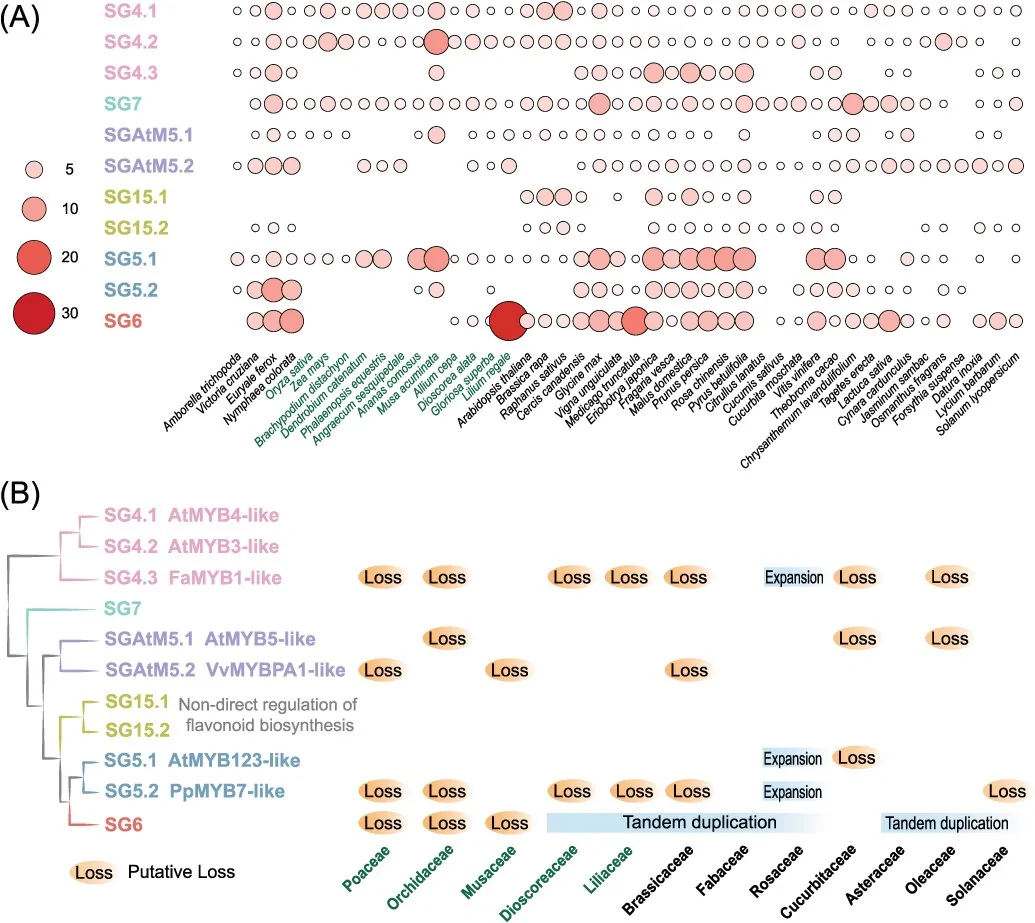
Gene count statistics and evolutionary characteristics of R2R3-MYBs in key flavonoid-regulatory subgroups across 44 horticultural species were analyzed, and ML phylogenetic trees of their homologous genes were constructed, with detailed trees provided in Supplementary Fig. S3. (A) Gene counts of R2R3-MYBs in key flavonoid-regulatory subgroups across 44 horticultural species. Each dot represents the gene count for an individual species. (B) Hypothetical evolutionary model of flavonoid-regulatory R2R3-MYBs in horticultural plants. Key expansion and putative loss events of each clade across different plant families are annotated. The evolutionary trajectory on the left is inferred from the phylogenetic tree in Supplementary Fig. S2; the putative gene loss and expansion patterns on the right are hypothesized based on the ML phylogenetic tree constructed using the 44 horticultural species in this study.

Functional divergence occurs among different flavonoid-regulatory MYB subgroups, accompanied by distinct patterns of gene loss or expansion across plant lineages. Clarifying the ancestral origins, functional evolutionary trajectories, and diversification principles of these subgroups is therefore critical. To further refine the phylogenetic classification of flavonoid-regulatory MYBs, we compiled amino acid sequences of functionally characterized flavonoid-regulatory R2R3–MYBs and constructed a rooted maximum likelihood (ML) tree (Fig. 2; Supplementary Fig. S1; Supplementary Data S1). This tree includes 270 nonredundant R2R3–MYBs, comprising 126 from *Arabidopsis* and 143 functionally validated sequences from representative horticultural species. AtCDC5 was used as the outgroup for rooting. Overall subfamily classification of the R2R3–MYB gene family follows established frameworks in *Arabidopsis* [8, 44, 45], partitioning all candidate R2R3–MYBs into 37 subgroups. Notably, flavonoid-regulatory R2R3–MYBs predominantly cluster in the defined key flavonoid-regulatory subgroups, although they are also sparsely distributed in SG22, SGAtM59, SG20, SG19, and SG13 with limited representation. This phylogenetic convergence implies that the key flavonoid-regulatory subgroups underwent an ancient expansion event early in angiosperm evolution, with subsequent functional divergence corresponding to the progressive complexity of flavonoid-mediated traits. To capture this diversity, these subgroups were further subdivided into six functional groups based on two criteria: (i) the primary flavonoid subclass they regulate, and (ii) their role as activators or repressors. Each group exhibits distinct sequence signatures and functional roles, with subclade-level differentiation reflecting additional specialization. Specifically, SG6 functions mainly as anthocyanin activators, SG5 mainly as proanthocyanidin or anthocyanin activators, SG7 mainly as flavonol activators, SGAtM5 mainly as general flavonoid activators, and SG4 mainly as flavonoid repressors [13]. By contrast, SG15 functions primarily as trichome regulators, indirectly related to flavonoid biosynthesis [46]. Consistent with the phylogenetic trees (constructed using orthogroup members of each subgroup across 44 horticultural species, as shown in Fig. 3 and Supplementary Fig. S3), further subclade differentiation was observed within the major groups: SG5 splits into SG5.1 (AtMYB123-like) and SG5.2 (PpMYB7-like); SGAtM5 partitions into SGAtM5.1 (AtMYB5-like) and SGAtM5.2 (VvMYBPA1-like); SG4 is classified into SG4.1 (AtMYB4-like), SG4.2 (AtMYB3-like), and SG4.3 (FaMYB1-like); and SG15 is divided into SG15.1 and SG15.2.

To resolve the evolutionary relationships and origins of key flavonoid-regulatory subgroups, we reconstructed a phylogenetic tree by incorporating sequences from bryophytes (*Marchantia polymorpha*, *Physcomitrium patens*), the fern *Selaginella moellendorffii*, the gymnosperm *Cycas panzhihuaensis*, and the early-diverging angiosperm *Amborella trichopoda* (Supplementary Fig. S2; Supplementary Data S2). Topological analysis of the rooted ML tree suggests that SG4 may represent the most ancient clade within key flavonoid-regulatory subgroups, occupying a basal phylogenetic position. This putative ancestral clade was likely followed by sequential divergence events that gave rise to SG7, and subsequently SGAtM5, while SG15, SG5, and SG6 are proposed to have emerged as more recently derived clades. This evolutionary sequence may align with a potential functional trajectory from broad-spectrum, general regulation to specialized roles: SG4 is proposed to function as a general repressor of the entire phenylpropanoid (including flavonoid) pathway [13], exerting wide-ranging inhibitory control, and may also represent a more ubiquitous regulatory type in early plants. SG7 likely evolved to specifically activate flavonol biosynthesis, consistent with the evolutionary principle of ‘from simple to complex’, as the flavonol biosynthesis pathway is simpler than that of anthocyanins and proanthocyanidins. Further functional specialization may have ensued, with SG6 and SG5 presumably undergoing gene duplication and neofunctionalization to adopt specialized roles in regulating anthocyanins and proanthocyanins, respectively. Notably, SG15 stands apart from other flavonoid-associated subfamilies: phylogenetic evidence suggests it may have originated from a gene duplication event in the last common ancestor of the SG6 + SG5 clade, followed by neofunctionalization. This evolutionary trajectory is further supported by its specialized role in trichome development rather than direct regulation of flavonoid biosynthesis [47]. This stepwise evolutionary trajectory may align with two key aspects during angiosperm evolution: the increasing complexity of plant–environment interactions and the evolutionary shift from essential survival-related traits to reproductive optimization. Early land plants may have relied solely on a simple ‘on–off’ regulatory switch (SG4) or more general regulatory mechanisms to meet core survival needs (e.g. UV protection). Flavonols (SG7), as early-evolving flavonoid products, are well suited to fulfill these survival-related functions. By contrast, later lineages (particularly flowering plants) likely required specialized regulators (e.g. SG6, SG5) to fine-tune anthocyanin/proanthocyanidin profiles, as these metabolites serve ‘reproductive optimization’ functions (e.g. attracting pollinators, promoting seed dispersal) that became critical with the emergence of flowers and fruits.

Next, we identified homologous R2R3–MYBs within the key flavonoid-regulatory subgroups across 44 horticultural species, ultimately subdividing them into 11 subclades (SG6, SG5.1, SG5.2, SG15.1, SG15.2, SG7, SGAtM5.1, SGAtM5.2, SG4.1, SG4.2, SG4.3) based on whether they form monophyletic clades in angiosperms. To ensure phylogenetic accuracy and mitigate potential methodological bias, we first constructed an integrated ML tree encompassing all candidate sequences to verify the robust support for these major subgroups. Subsequently, detailed phylogenetic analyses for the 11 subclades were performed using both IQ-TREE and FastTree to ensure topological congruence. We quantified gene counts across representative monocot and eudicot lineages and summarized lineage-specific expansion and putative loss events (Fig. 3; Supplementary Fig. S3; Supplementary Table S1; Supplementary Data S3). We acknowledge that these evolutionary inferences, particularly regarding putative gene loss, are currently based on a targeted selection of horticultural species. Therefore, future large-scale comparative genomic studies spanning a broader and more diverse range of plant lineages will be essential to further validate these findings and refine our understanding of R2R3–MYB evolutionary trajectories in angiosperms.

**Figure 4:**
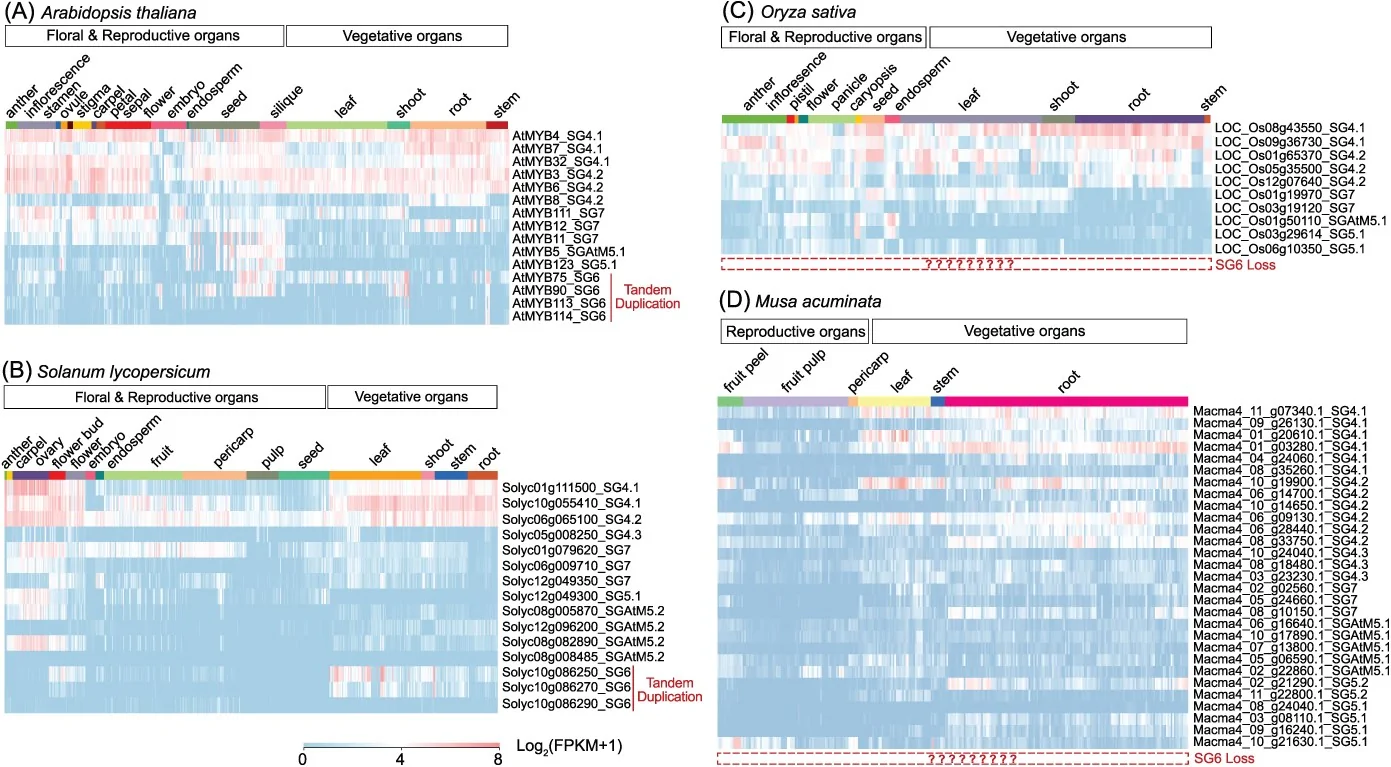
Tissue-specific expression patterns of R2R3-MYBs in key flavonoid-regulatory subgroups across different plant organs. (A) *A. thaliana*, (B) *S. lycopersicum*, (C) *O. sativa*, and (D) *M. acuminata*. Heatmaps show expression values standardized by log₂(FPKM+1), with columns clustered by tissue type (tissues belonging to the same category are grouped together). Plant organs were classified into vegetative and reproductive systems, and each R2R3-MYB was annotated with its corresponding subclade affiliation.

Additionally, 2048 expression datasets from 30 tissues were compiled to generate heatmaps, characterizing the spatiotemporal expression profiles and functional divergence of these R2R3–MYBs in four representative species: *Arabidopsis thaliana*, *Solanum lycopersicum*, *Oryza sativa*, and *Musa acuminata* (Fig. 4; Supplementary Tables S2–S9). Notably, Fig. 4 is designed to illustrate the variation in expression signatures between monocots and eudicots rather than generalize species-specific patterns. This analysis offers two primary insights: first, flavonoid-regulatory R2R3–MYBs have evolved tissue-specific expression patterns—particularly in reproductive organs and vegetative tissues—that likely align with their functional specialization; second, the observed intra-species expression variation reflects the functional refinement following gene duplication, providing context for the regulatory strategies unique to each subclade. While acknowledging the significant morphological disparities between the siliques of *Arabidopsis*, the grains of rice, and the fleshy fruits of tomato and banana, we included them within a broader ‘reproductive organ’ framework. This approach aims to explore the divergent evolutionary trajectories of regulatory strategies across these lineages, rather than asserting a direct functional or structural equivalence between these distinct fruit types. Collectively, our phylogenetic and expression analyses underscore the dynamic evolutionary divergence of subclades derived from key flavonoid-regulatory subgroups, highlighting the adaptive functional radiation of these subclades in horticultural plants.

## Regulatory mode shift between SG6 and SG5

Based on sequence homology, R2R3–MYBs regulating anthocyanin biosynthesis cluster into two functional subgroups: SG6 and SG5 (Figs 2 and 3). SG6 includes well-characterized anthocyanin regulators such as *Arabidopsis* AtPAP1/AtMYB75 [48] and petunia PhAN2 [49], while SG5, traditionally associated with proanthocyanidin regulation, also encompasses genes that mediate anthocyanin biosynthesis in monocots, exemplified by rice *OsC1* [50] and maize *ZmC1* [14]. Most anthocyanin-regulatory R2R3-MYBs in eudicot flowers or fruits belong to SG6; by contrast, monocots show lineage-specific divergence: those in Poaceae and Orchidaceae (e.g. OgMYB1, DhMYB2) [51, 52] fall into SG5, while lily LhMYB6/LhMYB12 and onion AcMYB1 are categorized into SG6 [53–55]. Anthocyanin-regulatory R2R3-MYBs have diverged along distinct trajectories in monocots and eudicots, with two primary hypotheses to explain this divergence. First, SG6 may have undergone neofunctionalization in monocots (e.g. Poaceae and Orchidaceae), followed by loss of the PAP1 (MYB75)-like clade. Second, SG6 may have evolved from SG5 to specialize in anthocyanin regulation in eudicots, while SG5 retained roles in proanthocyanidin biosynthesis.

## SG6: loss in monocots (especially Poaceae) vs expansion in eudicots

Our analysis of 44 horticultural species (Fig. 3; Supplementary Fig. S3; Supplementary Table S1) reveals distinct evolutionary patterns of SG6. We observed expansion via tandem duplication in most eudicot families (e.g. Brassicaceae, Fabaceae, Rosaceae, Asteraceae, Oleaceae, Solanaceae), including key crops such as peach, apple, grape, osmanthus, and tomato, while it remains evolutionarily conserved without tandem duplication in Cucurbitaceae. In contrast, SG6 exhibits putative loss in most monocot families (e.g. Poaceae, Orchidaceae, Musaceae), as it was not identified in the genomes of rice, maize, and banana. However, it is retained and expanded via tandem duplication in others (e.g. Liliaceae, Dioscoreaceae); notably, the retention of SG6 in Liliaceae is closely associated with floral pigmentation, consistent with its ornamental phenotypic requirements. The ornamental monocot lily thus represents a unique evolutionary case that highlights the complexity of anthocyanin biosynthesis in monocots. These duplicated SG6 MYBs often undergo subfunctionalization or neofunctionalization to regulate tissue-specific anthocyanin biosynthesis in petals, fruits, and leaves, a key evolutionary event driving floral and fruit color diversification in eudicots. For instance, *AtMYB113* and *AtMYB114* are highly expressed in vegetative organs, such as stems, while *AtMYB75* and *AtMYB90* exhibit high expression in both reproductive organs (e.g. siliques) and vegetative tissues (leaves, shoots, stems) in *Arabidopsis* (Fig. 4A; Supplementary Tables S2, S6). In tomato, *Solyc10g086250* is highly expressed in flowers, leaves, and shoots; *Solyc10g086270* is preferentially expressed in leaves and shoots; whereas *Solyc10g086290* shows low expression across tissues (Fig. 4B; Supplementary Tables S3, S7).

## SG5: retention in monocots and expansion in Rosaceae

SG5 is further subdivided into SG5.1 and SG5.2 based on whether they form monophyletic clades in angiosperms (Figs 2, 3; Supplementary Fig. S3). SG5.1 includes representative genes such as *Arabidopsis AtTT2/AtMYB123* [56], rice *OsC1* [50], and maize *ZmC1* [57], while SG5.2 is exemplified by peach *PpMYB7* [58]. Notably, SG5.1 is putatively lost in Cucurbitaceae, whereas SG5.2 is putatively absent from Poaceae, Orchidaceae, Liliaceae, Brassicaceae, and Solanaceae. As the mixed anthocyanin or proanthocyanidin regulator clade SG5 appears polyphyletic, we speculate that sequences from a broader taxonomic range may be required to more accurately resolve their complex phylogenetic relationships and regulatory patterns. Poaceae retains SG5.1 to regulate anthocyanin and proanthocyanidin biosynthesis; and a previous phylogenetic analysis of numerous Poaceae species identified an independently evolved MYB lineage (ncaMYB) that also mediates anthocyanin biosynthesis in this family [59]. Rosaceae exhibits significant expansion of SG5, putatively linked to the adaptive evolution of horticulturally relevant traits including fruit flavor, pigmentation, antioxidant activity, and stress resistance (e.g. PpMYB7 in peach, MdMYB11 and MdMYB12 in apple).

Expression analyses reveal distinct patterns across species: *Arabidopsis* and tomato each contain a single SG5 gene (i.e. *AtMYB123* and *Solyc12g049300*), both highly expressed in reproductive organs. In contrast, rice and banana harbor more SG5 genes with broader expression, showing activity in both vegetative and reproductive tissues (Fig. 4; Supplementary Tables S2–S9). This evolutionary pattern is consistent with a hypothetical functional compensation strategy in monocots: where SG6 is not evolved, lost, or reduced, SG5 may assume partial anthocyanin regulatory roles as an evolutionary adaptation. Functional characterization of anthocyanin and proanthocyanidin regulators across diverse monocots, eudicots, gymnosperms, and non-seed plants is essential to test this hypothesis, as such studies will clarify whether SG5-mediated compensation represents a conserved evolutionary strategy in flavonoid regulation with direct implications for horticultural trait improvement.

## Divergence in evolutionary patterns of other subgroups

In our study, the repressor SG4 is subdivided into three subclades (SG4.1, SG4.2, and SG4.3) based on whether they form monophyletic clades in angiosperms. SG4.1 and SG4.2 are relatively conserved with no significant loss across major families, while SG4.3 is putatively lost in multiple key lineages including monocots (e.g. Poaceae, Orchidaceae, Liliaceae) and eudicots (e.g. Brassicaceae, Cucurbitaceae, Oleaceae), but expanded in Rosaceae (Figs 2, 3; Supplementary Fig. S3). *Arabidopsis* and rice contain only SG4.1 and SG4.2, whereas tomato and banana possess all three subclades. Expression analyses reveal distinct diversification patterns: most SG4 genes across four representative species show ubiquitous tissue expression, consistent with their conserved roles as general repressors (Fig. 4; Supplementary Tables S2–S9). We propose two hypotheses to explain the evolutionary retention of these subclades. One is functional divergence, where subclades exhibit specialization for different metabolic pathways or abiotic stresses. Alternatively, the observed redundancy may represent a buffered regulatory network. Contrary to the view that redundant copies are evolutionary transient, these genes may be under purifying selection to maintain a critical inhibitory ‘threshold’. Their lineage-specific expansion or loss would thus reflect an adaptive tuning of the flavonoid toolkit to meet the specific physiological demands of different species.

SGAtM5 is further divided into SGAtM5.1 and SGAtM5.2, with SGAtM5.2 putatively lost in Poaceae but retained in lilies, orchids, and yams (Figs 2, 3; Supplementary Fig. S3). Phylogenetic distribution analyses confirm that *Arabidopsis*, rice, and banana exclusively possess SGAtM5.1, while tomato harbors only SGAtM5.2 (Fig. 4; Supplementary Fig. S3). SG7 is retained in the major families represented by the 44 horticultural species examined in this study; additionally, our phylogenetic analysis (Supplementary Fig. S3) and previous studies suggest a deep duplication in the SG7 lineage (MYB11/12 vs MYB111) [60], a pattern that warrants further investigation with expanded taxonomic diversity.

SG7 and SGAtM5 exhibit divergent expression profiles among genes and species, indicating subfunctionalization, which align with the earlier proposed trajectory of key flavonoid-regulatory subgroups evolving from broad-spectrum to specialized regulation (Fig. 4; Supplementary Tables S2–S9). For example, in tomato SG7, *Solyc01g079620* is highly expressed in all reproductive organs, while *Solyc06g009710* and *Solyc12g049350* are preferentially expressed in specific floral tissues. In tomato SGAtM5, *Solyc08g005870* and *Solyc08g082890* show high floral expression, whereas *Solyc12g096200* and *Solyc08g008485* are minimally expressed (Fig. 4B; Supplementary Tables S3, S7). In *Arabidopsis* SG7, *AtMYB111* is highly expressed in leaves and reproductive organs, *AtMYB12* in roots and reproductive organs, while *AtMYB11* is restricted to vegetative tissues; *Arabidopsis* SGAtM5 gene *AtMYB5* is specifically highly expressed in seeds and siliques (Fig. 4A; Supplementary Tables S2, S6). In banana SG7, all three genes show negligible expression in reproductive organs (Fig. 4D; Supplementary Tables S5, S9).

SG15 is subdivided into SG15.1 and SG15.2, which function mainly in trichome regulation and are not directly associated with flavonoid biosynthesis. To gain a more comprehensive understanding of the evolutionary history of SG15, we included sequences based on orthology to examine their presence across 44 horticultural species (Fig. 3; Supplementary Fig. S3; Supplementary Table S1). Our analysis revealed that SG15 is putatively lost in all monocots surveyed. Key unresolved questions remain: how is trichome formation regulated in lineages lacking SG15 orthologs, and why are three *Arabidopsis* MYBs required to control this single process? These questions demand substantive functional validation in future studies.

Collectively, phylogenetic and expression analyses underscore the substantial evolutionary dynamism in flavonoid-regulatory R2R3–MYB subgroups, particularly between monocots (especially Poaceae) and eudicots. Synthesizing these findings, we propose an evolutionary model of flavonoid-regulatory R2R3–MYBs in horticultural plants (Fig. 3B), which integrates their diversification history, expansion and putative loss events, and functional specialization within individual clades.

## Cross-species divergence within conserved flavonoid-regulatory subgroups: specialization of regulatory mechanisms

To investigate functional divergence of conserved flavonoid-regulatory subgroups across species, particularly in subclades regulating anthocyanin biosynthesis, we focus on key traits including spatiotemporal expression patterns (governing anthocyanin synthesis timing and tissue specificity), dependency on MBW complexes, and regulation of non-anthocyanin flavonoid classes. We categorized plants into monocots or eudicots and compiled a systematic dataset of functionally validated flavonoid-regulatory MYBs (Tables 1, 2). This dataset details species names and families, MYB types, subgroups, targeted flavonoid classes, regulatory roles (activation or repression), MBW complex dependency, and associated horticultural traits, providing a comprehensive overview of current research. Analyses reveal that R2R3-MYBs dominate reported flavonoid regulators. Anthocyanins and proanthocyanidins are the most commonly targeted flavonoid classes, though regulators of flavonols and other subtypes are also documented, and some R2R3–MYBs even coordinate multiple flavonoid pathways simultaneously. Regarding regulatory roles, all documented R3–MYBs function as flavonoid repressors [13], whereas R2R3–MYBs exhibit dual roles as activators or repressors.

**Table 1:** MYB TFs involved in the regulation of flavonoid biosynthesis in monocots.

**Species (Family)**	**Gene name**	**MYB type**	**Subgroup**	**Flavonoids type**	**Activate or repress**	**MBW complex or independently or both**	**Main horticultural traits influenced**	**Accession ID**	**Reference**
*Lilium* spp. (Liliaceae)	*LhMYB1*	R2R3	SG6	Anthocyanins	Activate	/	Flower pigmentation	/	[61]
	*LhMYB6*	R2R3	SG6	Anthocyanins	Activate	MBW	Flower pigmentation	BAJ05399.1	[54]
	*LhMYB12*	R2R3	SG6	Anthocyanins	Activate	MBW	Flower pigmentation	BAJ05398.1	[53]
	*LhR3MYB1*	R3	/	Anthocyanins	Repress	/	Flower pigmentation	BBG71951.1	[62]
	*LhR3MYB2*	R3	/	Anthocyanins	Repress	/	Flower pigmentation	BBG71952.1	[62]
	*LvMYB1*	R2R3	SG4.1	Anthocyanins	Repress	/	Flower pigmentation	MZ882485	[63]
	*LvMYB5*	R2R3	SG6	Anthocyanins	Activate	/	Flower pigmentation	MZ882486	[63]
*Tulipa fosteriana* (Liliaceae)	*TfMYB3*	R2R3	SG6	Anthocyanins	Activate	MBW	Flower pigmentation	AHY20034.1	[64]
	*TfMYB4*	R2R3	SG6	Anthocyanins	Activate	MBW	Flower pigmentation	AHY20035.1	[64]
	*TfMYB5*	R2R3	SG6	Anthocyanins	Activate	MBW	Flower pigmentation	AHY20036.1	[64]
*Bletilla striata* (Orchidaceae)	*BsMYB36*	R2R3	SG7	Flavonoids	Activate	/	Stress resistance, nutritional value	/	[65]
	*BsMYB51*	R2R3	SG5.1	Anthocyanins, proanthocyanidins	Activate	/	Stress resistance, nutritional value	/	[65]
*Oncidium* spp. (Orchidaceae)	*OgMYB1*	R2R3	SG5.1	anthocyanins	activate	/	flower pigmentation	EF570115	[51]
*Phalaenopsis aphrodite* (Orchidaceae)	*PeMYB2*	R2R3	SG5.1	Anthocyanins	Activate	Both	Flower pigmentation	AIS35919.1	[66]
	*PeMYB11*	R2R3	SG5.1	Anthocyanins	Activate	MBW	Flower pigmentation	AIS35928.1	[66]
	*PeMYB12*	R2R3	SG5.1	Anthocyanins	Activate	Both	Flower pigmentation	AIS35929.1	[66]
*Dendrobium* spp. (Orchidaceae)	*DhMYB2*	R2R3	SG5.1	Anthocyanins	Activate	MBW	Flower pigmentation	AQS79852.1	[52]
*Musa acuminata* (Musaceae)	*MaMYBPA1*	R2R3	SG5.1	Proanthocyanidins	Activate	Both	Stress resistance, nutritional value	ON981385	[67]
	*MaMYBPA2*	R2R3	SG5.1	Proanthocyanidins	Activate	Both	Stress resistance, nutritional value	ON981386	[67, 68]
	*MaMYBA1*	R2R3	SG5.1	Anthocyanins	Activate	MBW	Nutritional value	Ma06_t05960.1	[68]
	*MaMYBA2*	R2R3	SG5.1	Anthocyanins	Activate	MBW	Nutritional value	Ma09_t27990.1	[68]
	*MaMYB4*	R2R3	SG4.3	Anthocyanins, proanthocyanidins	Repress	Both	Stress resistance, nutritional value	Ma03_t21920.1	[67, 69]
	*MaMYBPR1/MaMYB4*	R2R3	SG4.3	Anthocyanins, proanthocyanidins	Repress	MBW	Stress resistance, nutritional value	ON981392	[67, 69]
	*MaMYBPR2*	R2R3	SG4.3	Anthocyanins	Repress	MBW	Stress resistance, nutritional value	ON981393	[67]
	*MaMYBPR3*	R2R3	SG4.3	Anthocyanins	Repress	MBW	Stress resistance, nutritional value	ON981394	[67]
	*MaMYBPR4*	R2R3	SG4.3	Anthocyanins	Repress	MBW	stress resistance, nutritional value	ON981395	[67]
*Allium cepa* (Amaryllidaceae)	*AcMYB1*	R2R3	SG6	Anthocyanins	Activate	MBW	Fruit pigmentation, nutritional value	AQP25672.1	[55]
*Narcissus tazetta* (Amaryllidaceae)	*NtMYB2*	R2R3	SG4.1	Anthocyanins	Repress	/	Flower pigmentation	ATO58377.1	[70]
	*NtMYB3*	R2R3	SG4.1	Anthocyanins	Repress	/	Flower pigmentation	AGO33166.1	[71]
*Elaeis guineensis* (Arecaceae)	*VIRESCENS*	R2R3	SG6	Anthocyanins	Activate	MBW	Fruit pigmentation	KJ789862	[72]
*Zea mays* (Poaceae)	*ZmC1*	R2R3	SG5.1	Anthocyanins	Activate	MBW	Pigmentation	Zm00001d044975_P001	[57]
	*ZmPL1*	R2R3	SG5.1	Anthocyanins	Activate	MBW	Pigmentation	Zm00001d037118_P001	[57, 73]
	*ZmP1*	R2R3	SG7	Phlobaphenes	Activate	Independently	Pigmentation, nutritional value	Zm00001d028850_P001	[74]

‘/’ indicates that this information is not mentioned in the reference.

**Table 2:** MYB TFs involved in the regulation of flavonoid biosynthesis in eudicots.

**Species (Family)**	**Gene name**	**MYB type**	**Subgroup**	**Flavonoids type**	**Activate or repress**	**MBW complex or independently or both**	**Main horticultural traits influenced**	**Accession ID**	**Reference**
*Vitis vinifera* (Vitaceae)	*VvMYBF1*	R2R3	SG7	Flavonols	Activate	Independently	Stress resistance, nutritional value	FJ948477	[75]
	*VvMYBA1*	R2R3	SG6	Anthocyanins	Activate	MBW	Fruit pigmentation, nutritional value	AB097923	[76]
	*VvMYBA2*	R2R3	SG6	Anthocyanins	Activate	MBW	Fruit pigmentation, nutritional value	AB252699	[76]
	*VvMYBPA1*	R2R3	SGAtM5.2	Proanthocyanidins	Activate	MBW	Stress resistance, nutritional value	AM259485	[77]
	*VvMYBPA2*	R2R3	SG5.1	Proanthocyanidins	Activate	/	stress resistance, nutritional value	EU919682	[78]
	*VvMYB5a*	R2R3	SGAtM5.1	Anthocyanins, proanthocyanidins	Activate	MBW	Stress resistance, nutritional value	AAS68190	[79]
	*VvMYB5b*	R2R3	SGAtM5.1	Anthocyanins, proanthocyanidins	Activate	MBW	Stress resistance, nutritional value	AAX51291	[79]
	*VvMYBPAR*	R2R3	SG5.1	Proanthocyanidins	Activate	MBW	Stress resistance, nutritional value	AB911341	[80]
	*VvMYBC2-L1*	R2R3	SG4.3	Flavonoids	Repress	MBW	Stress resistance, pigmentation	EU181425	[81]
	*VvMYBC2-L2*	R2R3	SG4.3	Flavonoids	Repress	MBW	stress resistance	ACX50288	[82]
	*VvMYBC2-L3*	R2R3	SG4.3	Flavonoids	Repress	MBW	Stress resistance, pigmentation	KM046932	[81]
	*VvMYB44–1*	R2R3	SG22	Anthocyanins	Repress	Both	Fruit pigmentation	VIT_18s0001g09850	[83]
*Populus trichocarpa* (Salicaceae)	*PtrMYB115*	R2R3	SGAtM5.2	Proanthocyanidins	Activate	MBW	Stress resistance	XM_002302608.2	[84]
	*PtrMYB134*	R2R3	SG5.1	Proanthocyanidins	Activate	MBW	Stress resistance	Potri.006G221800.1	[84]
	*PtrMYB165*	R2R3	SG4.3	Flavonoids	Repress	MBW	Stress resistance	MH746926	[85]
	*PtrMYB182*	R2R3	SG4.3	Anthocyanins, proanthocyanidins	Repress	MBW	Stress resistance	KP723392	[86]
	*PtrMYB194*	R2R3	SG4.3	Flavonoids	Repress	MBW	Stress resistance	MH746927	[85]
	*PtrMYB57*	R2R3	SG4.1	Anthocyanins, proanthocyanidins	Repress	MBW	Stress resistance	Potri.T011400.1	[87]
	*PtrRML1*	R3	/	Anthocyanins	Repress	MBW	Stress resistance	Potri.012G031200	[88]
*Medicago sativa* (Fabaceae)	*MtPAR*	R2R3	SG5.2	Proanthocyanidins	Activate	MBW	Stress resistance	HQ337434	[89]
	*MtMYB5*	R2R3	SGAtM5.1	Proanthocyanidins	Activate	MBW	Stress resistance, pigmentation	XM_003601561	[90]
	*MtMYB14*	R2R3	SG5.1	Proanthocyanidins	Activate	MBW	Stress resistance, pigmentation	Medtr4g125520.1	[90]
	*MtMYB134*	R2R3	SG7	Flavonols	Activate	Independently	Stress resistance	MW176076	[91]
	*MtLAP1*	R2R3	SG6	Anthocyanins	Activate	MBW	Stress resistance	FJ199998	[92]
	*MtMYB2*	R2R3	SG4.3	Anthocyanins, proanthocyanidins	Repress	MBW	Stress resistance, pigmentation	XM_003616340	[93]
*Glycine max* (Fabaceae)	*GmTT2A*	R2R3	SG5.1	Anthocyanins, proanthocyanidins	Activate	MBW	Seed pigmentation	XP_003541301.1	[94]
	*GmTT2B*	R2R3	SG5.1	Anthocyanins, proanthocyanidins	Activate	MBW	Seed pigmentation	NM_001355658.1	[94]
	*GmMYB5A*	R2R3	SGAtM5.1	Anthocyanins, proanthocyanidins	Activate	MBW	Seed pigmentation	NM_001254563.1	[94]
	*GmMYBA2*	R2R3	SG6	Anthocyanins	Activate	MBW	Seed pigmentation	Glyma.09G235100	[95]
	*GmMYBR*	R2R3	SG4.3	Anthocyanins	Repress	MBW	Seed pigmentation	Glyma.20G013000	[95]
*Ammopiptanthus nanus* (Fabaceae)	*AnaMYB87*	R2R3	SG4.1	Anthocyanins	Repress	/	Stress resistance	EVM0025673.1	[96]
*Spatholobus suberectus* (Fabaceae)	*SsMYB158*	R2R3	SGAtM59	Flavonoids	Activate	/	Medicinal value	TKY48447.1	[97]
*Diospyros kaki* (Ebenaceae)	*DkMYB2*	R2R3	SG5.1	Proanthocyanidins	Activate	/	Fruit flavor	/	[98]
	*DkMYB4*	R2R3	SGAtM5.2	Proanthocyanidins	Activate	/	Fruit flavor	AB503701	[99]
	*DkMYB14*	R2R3	SG4.2	Proanthocyanidins	Repress	Independently	Fruit flavor	MG551309	[100]
*Prunus persica* (Rosaceae)	*PpMYB7*	R2R3	SG5.2	Proanthocyanidins	Activate	MBW	Fruit flavor, nutritional value	KT159231	[58]
	*PpMYB15*	R2R3	SG7	Flavonols	Activate	Independently	Fruit flavor, nutritional value	Prupe.8G270000	[101]
	*PpMYBF1*	R2R3	SG7	Flavonols	Activate	Independently	Fruit flavor, nutritional value	Prupe.1G125300	[101]
	*PpMYB10*	R2R3	SG6	Anthocyanins	Activate	MBW	Fruit pigmentation, nutritional value	ABX79945.1	[102]
	*Peace*	R2R3	SG5.2	Anthocyanins	Activate	/	Flower pigmentation	AB897866	[103]
	*PpMYB18*	R2R3	SG4.3	Anthocyanins, proanthocyanidins	Repress	MBW	Fruit pigmentation, nutritional value	ALO81021.1	[104]
*Rosa chinensis* (Rosaceae)	*RcMYB1*	R2R3	SG6	Anthocyanins	Activate	Both	Flower pigmentation	XP_024189921.1	[105]
*Rosa rugosa* (Rosaceae)	*RrMYB5*	R2R3	SGAtM5.2	Proanthocyanidins	Activate	Both	Stress resistance	AYP10274.1	[106]
	*RrMYB10*	R2R3	SG5.2	Proanthocyanidins	Activate	Both	Stress resistance	CCA29099.1	[106]
	*RrMYB113*	R2R3	SG6	Anthocyanins	Activate	/	Flower pigmentation	AXQ12351.1	[107]
	*RrMYB111*	R2R3	SG7	Flavonols	Activate	Independently	Flower pigmentation	XM_062138075	[108]
	*RrMYB12*	R2R3	SG7	Flavonols	Activate	Independently	Flower pigmentation	XM_062132433	[108]
*Malus domestica* (Rosaceae)	*MdMYB1*	R2R3	SG6	Anthocyanins	Activate	/	Fruit pigmentation	ADQ27443.1	[109]
	*MdMYB3*	R2R3	SG4.2	Anthocyanins	Repress	/	Fruit pigmentation	AEX08668.1	[110]
	*MdMYB9*	R2R3	SG5.1	Anthocyanins, proanthocyanidins	Activate	MBW	Fruit pigmentation, nutritional value	ABB84757.1	[111]
	*MdMYB10*	R2R3	SG6	Anthocyanins	Activate	MBW	Fruit pigmentation	ACQ45201.1	[112]
	*MdMYB11*	R2R3	SG5.1	Anthocyanins, proanthocyanidins	Activate	MBW	Fruit pigmentation, nutritional value	AAZ20431.1	[111]
	*MdMYB12*	R2R3	SG5.1	Proanthocyanidins	Activate	MBW	Nutritional value	XP_008337875.1	[113]
	*MdMYB22*	R2R3	SG7	Flavonols	Activate	Independently	Nutritional value	AAZ20438.1	[113]
	*MdMYB110a*	R2R3	SG6	Anthocyanins	Activate	/	Fruit pigmentation	AFC88038.1	[114]
	*MdMYBA*	R2R3	SG6	Anthocyanins	Activate	Both	Fruit pigmentation	BAF80582.1	[115]
	*MdMYBPA1*	R2R3	SGAtM5.2	Proanthocyanidins	Activate	MBW	Fruit pigmentation, stress resistance	AIF70441.1	[116]
	*MdMYB16*	R2R3	SG4.1	Anthocyanins	Repress	Both	Nutritional value	ADL36756.1	[117]
*Pyrus* spp. (Rosaceae)	*PbMYB12b*	R2R3	SG7	Flavonols	Activate	Independently	Nutritional value	XP_009339792.2	[118]
	*PyMYB10*	R2R3	SG6	Anthocyanins	Activate	MBW	Fruit pigmentation	ADN26574.1	[119]
	*PyMYB114*	R2R3	SG6	Anthocyanins	Activate	MBW	Fruit pigmentation	ASY06612.1	[120]
	*PpMYB140*	R2R3	SG4.2	Anthocyanins	Repress	MBW	Fruit pigmentation	Pbr000876.1	[121]
*Eriobotrya japonica* (Rosaceae)	*EjMYB5*	R2R3	SGAtM5.1	Proanthocyanidins	Activate	/	Nutritional value	Eja04G026110	[122]
*Antirrhinum majus* (Plantaginaceae)	*AmROS1*	R2R3	SG6	Anthocyanins	Activate	/	Flower pigmentation	ABB83826.1	[123]
	*AmROS2*	R2R3	SG6	Anthocyanins	Activate	/	Flower pigmentation	ABB83827.1	[123]
	*AmVENOSA*	R2R3	SG6	Anthocyanins	Activate	/	Flower pigmentation	ABB83828.1	[123]
	*AmMYB340*	R2R3	/	Flavonoids	Activate	/	Flower pigmentation	X70874.1	[124]
	*AmMYB305*	R2R3	/	Flavonoids	Activate	/	Flower pigmentation	X70873.1	[124]
	*AmMYB308*	R2R3	SG4.1	Flavonoids	Repress	/	Flower pigmentation	P81393	[125]
	*AmMYB330*	R2R3	SG4.2	Flavonoids	Repress	/	Flower pigmentation	P81395	[125]
*Petunia hybrida* (Solanaceae)	*PhAN2*	R2R3	SG6	Anthocyanins	Activate	/	Flower pigmentation	AAF66727.1	[49]
	*PhPH4*	R2R3	SGAtM5.1	Anthocyanins	Activate	MBW	Flower pigmentation	AAY51377.1	[126]
	*PhDPL*	R2R3	SG6	Anthocyanins	Activate	MBW	flower pigmentation	ADW94950.1	[127]
	*PhPHZ*	R2R3	SG6	Anthocyanins	Activate	MBW	Flower pigmentation	ADW94951.1	[127]
	*PhMYB27*	R2R3	SG4.3	Anthocyanins	Repress	MBW	Flower pigmentation	AHX24372.1	[128]
	*PhMYBx*	R3	/	Anthocyanins	Repress	MBW	Flower pigmentation	AHX24371.1	[128]
*Solanum lycopersicum*(Solanaceae)	*SlMYBATV*	R3	/	Anthocyanins	Repress	MBW	Fruit pigmentation	MF197509	[129]
*Solanum tuberosum* (Solanaceae)	*StMYB200*	R2R3	SG6	Anthocyanins	Activate	MBW	Fruit pigmentation	DM8C10G21200	[130]
	*StMYB210*	R2R3	SG6	Anthocyanins	Activate	MBW	Fruit pigmentation	DM8C10G21210	[130]
*Camellia sinensis* (Theaceae)	*CsMYB5a*	R2R3	SG5.1	Proanthocyanidins	Activate	MBW	Leaf flavor, stress resistance	KAF5936438.1	[131]
	*CsMYB5b*	R2R3	SG5.1	Proanthocyanidins	Activate	MBW	Leaf flavor, stress resistance	XP_028089226.1	[131]
	*CsMYB5e*	R2R3	SGAtM5.2	Proanthocyanidins	Activate	MBW	Leaf flavor, stress resistance	XP_028116920.1	[131]
	*CsMYB75*	R2R3	SG6	Anthocyanins	Activate	MBW	Leaf pigmentation	UYR25389.1	[131]
*Camellia japonica* (Theaceae)	*CjMYB114*	R2R3	SG6	Anthocyanins	Activate	MBW	Flower pigmentation	ON646219	[132]
*Actinidia chinensis* (Actinidiaceae)	*AcMYBF110*	R2R3	SG6	Anthocyanins	Activate	MBW	Fruit pigmentation, nutritional value	AYJ72555.1	[133]
	*AcMYB123*	R2R3	SG5.1	Anthocyanins	Activate	MBW	Fruit pigmentation, nutritional value	QAT77713.1	[134]
*Actinidia arguta* (Actinidiaceae)	*AaMYB61*	R2R3	SG13	Anthocyanins	Activate	MBW	Fruit pigmentation, nutritional value	PQ179273	[135]
	*AaMYBC1*	R2R3	SG5.1	Anthocyanins	Activate	MBW	Fruit pigmentation	MN239491	[136]
*Paeonia suffruticosa* (Paeoniaceae)	*PsMYB58*	R2R3	SG6	Anthocyanins	Activate	Both	Flower pigmentation	AMW36067	[137]
*Morella rubra* (Myricaceae)	*MrMYB1*	R2R3	SG6	Anthocyanins	Activate	MBW	Fruit pigmentation, nutritional value	GQ340767	[138]
	*MrMYB12*	R2R3	SG7	Flavonols	Activate	Independently	Nutritional value	KAB1200696.1	[139]
*Zinnia elegans* (Asteraceae)	*ZeMYB32*	R2R3	SG4.1	Anthocyanins	Repress	MBW	flower pigmentation	OQ418108	[140]
	*ZeMYB9*	R2R3	SG6	Anthocyanins	Activate	MBW	Flower pigmentation	ON959236	[140]
*Carthamus tinctorius* (Asteraceae)	*CtMYB76*	R2R3	SG7	flavonols	Activate	Independently	Nutritional and medicinal value	CtAH10T0102600.1	[141]
*Brassica oleracea* (Brassicaceae)	*BoMYBL2b*	R3	/	Anthocyanins	Repress	Independently	Leaf pigmentation, nutritional value	Bo6g112670	[142]
	*BroMYB34*	R2R3	/	Flavonoids	Activate	Independently	Nutritional value, stress resistance	BroT26.C07gMYB34	[143]
*Raphanus sativus* (Brassicaceae)	*RsPAP2*	R2R3	SG6	Anthocyanins	Activate	MBW	Root pigmentation	MT459822	[144]
	*RsMYB1*	R2R3	SG6	Anthocyanins	Activate	/	Root pigmentation	Rsa10033919	[145]
*Daucus carota* (Apiaceae)	*DcMYB113*	R2R3	SG6	Anthocyanins	Activate	MBW	Fruit pigmentation, nutritional value	DCAR_008994	[146]
*Dianthus caryophyllus* (Caryophyllaceae)	*DcMYB1*	R2R3	SG6	Anthocyanins	Activate	MBW	Petal pigmentation	Dca7400	[147]
	*DcMYB2*	R2R3	SG4.2	Anthocyanins	Repress	MBW	Petal pigmentation	Dca11837	[147]
*Beta vulgaris* (Amaranthaceae)	*BvMYB1*	R2R3	SG6	Betalains (not flavonoids)	Activate	Independently	Root pigmentation	JF432080	[148]
*Amaranthus tricolor* (Amaranthaceae)	*AtrMYB72*	R2R3	SG6	Betalains (not flavonoids)	Activate	Independently	Leaf pigmentation	/	[149]
*Fagopyrum tataricum* (Polygonaceae)	*FtMYB163*	R2R3	SG7	Flavonols	Activate	Independently	Nutritional value, stress resistance	FtPinG0009153900.01	[150]
	*FtMYB6*	R2R3	SG7	Flavonols	Activate	Independently	Nutritional value, stress resistance	MT333222	[151]

‘/’ indicates that this information is not mentioned in the reference.

### Spatiotemporal expression patterns

Even within the same anthocyanin-regulatory subgroup (SG6 or SG5), spatiotemporal expression patterns (across organs and developmental stages) diverge among different species and even among varieties of the same species. For instance, *RsPAP2* (SG6) [144], a key regulator of red skin color in radish taproots, exhibits differential expression across genotypes with distinct skin and flesh coloration. In three red-skinned genotypes, its expression is significantly higher in root skin than in flesh; conversely, in the green-skinned, red-fleshed genotype, *RsPAP2* is markedly upregulated in root flesh compared to skin. Notably, no significant expression differences between skin and flesh are observed in four white-skinned and white-fleshed genotypes or one green-skinned and green-fleshed genotype [144]. However, in rose, *RcMYB1* (SG6) [105] transcript levels show stage-specific dynamics: they gradually increase alongside anthocyanin accumulation, peak at developmental stage S5, and then decrease as anthocyanins degrade. Further analysis across roses with distinct flower colors reveals relatively high *RcMYB1* expression in red roses, with equally high levels in green, yellow, and white roses, but lower expression in several blue-purple roses [105]. In the monocot orchid *Phalaenopsis*, differential expression of SG5 genes *PeMYB2*, *PeMYB11*, and *PeMYB12* [66] correlates with red flower color formation, supporting their involvement in regulating anthocyanin biosynthesis. In the variety with white sepals/petals and a yellow lip, *PeMYB2* expression in sepals correlates with pink coloration on sepal abaxial surfaces, while *PeMYB11* expression in lips is associated with red lip spots; *PeMYB2* and *PeMYB12* show little expression in these tissues, with only weak *PeMYB11* expression in lips. In the variety with white sepals/petals and a red lip, *PeMYB11* and *PeMYB12* are expressed in red lips (with *PeMYB12* showing higher expression), and *PeMYB2* expression in sepals/petals corresponds to pink abaxial surfaces of these organs. In the red-flowered variety, *PeMYB2*, *PeMYB11*, and *PeMYB12* are highly expressed in floral organs overall, except for *PeMYB2* which shows lower expression in lips [66].

### MBW dependency

Current research on the divergence of MYB regulatory mechanisms between monocots and eudicots has focused on two primary dimensions. First, recent studies emphasize the multifaceted architecture of the MBW complex, which extends beyond a simple presence-or-absence dependency to encompass high degrees of combinatorial complexity. Rather than adhering to a binary regulatory mode, MBW-mediated control operates through a sophisticated ‘mix-and-match’ logic, where the specific identity and selective assembly of diverse MYB and bHLH subunits dictate functional specificity. Second, research highlights the divergence in the repertoires of targeted structural genes within the flavonoid biosynthetic pathway. In maize, anthocyanin biosynthetic genes (e.g. *CHS*, *CHI*, *F3H*, *DFR*, *ANS/LDOX*, *UFGT*) are coordinately activated by an MBW complex comprising C1/PL1 family MYBs, R1/B1 family bHLHs, and WD40 scaffold proteins [57]. Allelic variation in these components dictates tissue- and developmental stage-specific pigmentation, representing a conserved MBW-dependent activation mode for anthocyanin biosynthesis. Notably, MYB-mediated repression of flavonoid biosynthesis can operate independently of the MBW complex, relying on a single MYB to exert inhibitory effects (Tables 2, 3). For instance, persimmon DkMYB14 can directly targeting the promoters of proanthocyanidin biosynthetic genes (*DkF3′5′H*, *DkANR*) to inhibit their transcription without the need for bHLH or WD40 cofactors [100]. Grape VvMYB44–1 employs a dual inhibitory mechanism: it directly binds the promoters of anthocyanin biosynthetic genes (*VvDFR*, *VvUFGT*) to suppress anthocyanin accumulation, while also competitively binding to the WD40 subunit (VvWDR2) of the MBW complex to disrupt its assembly and transcriptional activity [83].

**Table 3:** MYB TFs involved in the regulation of flavonoid biosynthesis in *A. thaliana*, *F. × ananassa*, *O. sativa*, and *F. hybrida.*

**Species**	**Gene name**	**MYB type**	**Subgroup**	**Flavonoids type**	**Activate or repress**	**MBW complex or independently or both**	**Experimental validation types**	**Accession ID**	**Reference**
*A. thaliana*	*AtMYB11*	R2R3	SG7	Flavonols	Active	independently	protoplast transient transfection; Pro:GUS fusion; T-DNA mutant analysis; DPBA in situ staining; HPLC-PDA-ESI/MS analysis	AT3G62610	[152]
	*AtMYB12*	R2R3	SG7	Flavonols	Active	independently	protoplast transient transfection; Pro:GUS fusion; T-DNA mutant analysis; DPBA in situ staining	AT2G47460	[152, 153]
	*AtMYB111*	R2R3	SG7	Flavonols	Active	independently	protoplast transient transfection; Pro:GUS fusion; T-DNA mutant analysis; DPBA in situ staining	AT5G49330	[152, 153]
	*AtPAP1/AtMYB75*	R2R3	SG6	Anthocyanins	Active	MBW	OE & complementation; genetic cross; transgenic transformation	AT1G56650	[154]
	*AtPAP2/AtMYB90*	R2R3	SG6	Anthocyanins	Active	MBW	OE; transgenic transformation	AT1G66390	[154]
	*AtMYB113*	R2R3	SG6	Anthocyanins	Active	MBW	OE; genetic cross; Pro:GUS fusion; RNAi silencing; transgenic transformation	AT1G66370	[48]
	*AtMYB114*	R2R3	SG6	Anthocyanins	Active	MBW	OE; genetic cross; Pro:GUS fusion; RNAi silencing; transgenic transformation	AT1G66380	[48]
	*AtTT2/AtMYB123*	R2R3	SG5.1	Proanthocyanidins	Active	MBW	OE & complementation; mutant phenotype analysis; transgenic transformation	AT5G35550	[56, 155]
	*AtMYB5*	R2R3	SGAtM5.1	Proanthocyanidins	Active	MBW	OE; ISH; complementation; Y2H; mutant phenotype analysis; Pro:GUS fusion	AT3G13540	[155, 156]
	*AtMYB4*	R2R3	SG4.1	Anthocyanins, proanthocyanidins	Repress	Both	OE & mutant phenotype analysis; Y1H; Y2H; BiFC; Co-IP; EMSA; transient expression; ChIP-qPCR	AT4G38620	[157]
	*AtMYB7*	R2R3	SG4.1	Flavonols, anthocyanins	Repress	Both	OE & mutant phenotype analysis; Y2H; BiFC; Co-IP; Pro:GUS fusion	AT2G16720	[158]
	*AtMYB32*	R2R3	SG4.1	Anthocyanins, proanthocyanidins	Repress	Both	mutant phenotype analysis; Y2H; BiFC; Co-IP; Pro:GUS fusion	AT4G34990	[158]
	*AtMYB3*	R2R3	SG4.2	Anthocyanins	Repress	With LNK1/2	OE & mutant phenotype analysis; Y2H; BiFC; EMSA; transient transactivation; ChIP-qPCR	AT1G22640	[159]
	*AtMYB21*	R2R3	SG19	Flavonols	Active	Independently and MYB triad	dual-luciferase assay; EMSA; ChIP; mutant analysis; complementation	AT3G27810	[160–162]
	*AtMYB24*	R2R3	SG19	Flavonols	Active	Independently and MYB triad	dual-luciferase assay; EMSA; ChIP; mutant analysis; complementation	AT5G40350	[160–162]
	*AtMYB99*	R2R3	SGAtM85	Flavonols	Active	Independently and MYB triad	dual-luciferase assay; ChIP; mutant analysis; complementation	AT5G62320	[160–162]
	*AtMYBL2*	R3	/	Anthocyanins, proanthocyanidins	Repress	MBW	OE & mutant phenotype analysis; Y2H; transient transactivation; protein domain deletion; Pro:GUS fusion	AT1G71030	[163, 164]
	*AtCPC*	R3	/	Anthocyanins, proanthocyanidins	Repress	MBW	OE & mutant phenotype analysis; Y2H; transient promoter activity	AT2G46410	[165]
*Fragaria × ananassa*	*FaMYB10*	R2R3	SG6	Flavonoids	Active	Both	RNAi silencing & OE; promoter activity & tissue expression; agroinfiltration transient expression	EU155162	[166]
	*FaMYB123*	R2R3	SG5.2	Anthocyanins, proanthocyanidins	Active	MBW	RNAi silencing & OE; Y2H; BiFC; transient transactivation	MW081987	[167]
	*FaMYB5*	R2R3	SGAtM5.1	Anthocyanins, proanthocyanidins	Active	MBW	transient OE; Y2H; BiFC; dual-luciferase assay; EMSA; protein degradation & ubiquitination	MW700311	[168, 169]
	*FaMYB9*	R2R3	SG5.1	Proanthocyanidins	Active	MBW	Y2H; mutant complementation; transient expression	JQ989281	[170]
	*FaMYB11*	R2R3	SG5.1	Proanthocyanidins	Active	MBW	Y2H; mutant complementation; transient expression	JQ989282	[170]
	*FaMYB1*	R2R3	SG4.3	Anthocyanins, flavonols	Repress	MBW	heterologous OE; Y2H; DNA gel blot	AF401220	[171]
*O. sativa*	*OsC1*	R2R3	SG5.1	Anthocyanins	Active	MBW	transient activation; agro-mediated transformation	LOC_Os06g10350	[172, 173]
	*OsKala3/OsMYB3*	R2R3	SG5.1	Anthocyanins	Active	MBW	Y2H; protoplast transient transfection; OE; CRISPR-KO;	LOC_Os03g29614	[174, 175]
	*OsP1*	R2R3	SG7	Flavonols	Active	Independently	CRISPR-KO; agro-mediated OE; Y2H; dual-luciferase transient transcriptional activity	LOC_Os03g19120	[172]
*F. hybrida*	*FhPAP1*	R2R3	SG6	Anthocyanins, proanthocyanidins	Active	MBW	Agro-mediated transient expression; stable transformation; Protoplast transient transfection; BiFC; ChIP-qPCR	MT210093	[176]
	*FhMYB5*	R2R3	SGAtM5.1	Anthocyanins, proanthocyanidins	Active	MBW	Protoplast transient transfection; BiFC; promoter activation; stable transformation	MK168337	[177]
	*FhMYB27*	R2R3	SG4.3	Anthocyanins, proanthocyanidins	Repress	MBW	Protoplast transient transfection; BiFC; promoter activation/repression; stable transformation; Y2H; ChIP-qPCR	MT210094	[178]
	*FhMYBPA1/2/3/4*	R2R3	SG5.1	Proanthocyanidins	Active	MBW	Y2H; BiFC; protoplast transient transfection; stable transformation; promoter truncation/mutation; histochemical staining; transactivation	/	[179]
	*FhMYBF1/2/3/4*	R2R3	SG7	Flavonols	Active	Independently	Transactivation; protoplast transient transfection; promoter truncation/mutation; ChIP-qPCR	MT741688	[180]
	*FhMYB21L2*	R2R3	SG19	Flavonols	Active	Independently	Transactivation; protoplast transient transfection; promoter truncation/mutation; ChIP-qPCR	MT741684	[180]
	*FhMYBx*	R3	/	Anthocyanins, proanthocyanidins	repress	MBW	Protoplast transient transfection; BiFC; promoter repression; stable transformation; Y2H	MT210095	[178]

‘/’ indicates that this information is not mentioned in the reference.

The Caryophyllales emerge as a compelling model for eudicot divergence, defined by the independent loss of the functional MBW complex across multiple families. While anthocyanins are ubiquitous in higher plants, they have been largely replaced by betalains in most species of this order, with these two red pigment classes (anthocyanins and betalains) never documented to coexist in a single plant [181]. A key feature of MYBs in Caryophyllales is the presence of two paralogous lineages (PAP1a and PAP1b), which exhibit a ‘reciprocal loss/retention’ pattern across clades with independent origins of betalain biosynthesis [182]. The loss of functional MBW complexes is a central driver of the anthocyanin-to-betalain shift: Caryophyllales MYB homologs (e.g. PAP1a/b) are unable to form functional MBW complexes due to intrinsic structural variations. For instance, the impaired anthocyanin synthesis in spinach is primarily attributed to six amino acid variations in PAP-like MYBs, which abrogate their functional activity and thus prevent the activation of late anthocyanin biosynthetic genes, such as *DFR* and *ANS* [183]. In contrast, betalain biosynthesis has undergone evolutionary specialization to be regulated independently by MYBs, without reliance on the MBW complex. *Beta vulgaris* has evolved a betalain-specific R2R3*-*MYB (BvMYB1, belonging to SG6) that activates the betalain biosynthetic pathway autonomously. Lacking key amino acids required for bHLH interaction, BvMYB1 does not form an MBW complex but directly binds to the promoters of core betalain biosynthetic genes (*BvDODA1* and *BvCYP76AD1*) to initiate their expression [148]. Similarly, in *Amaranthus tricolor*, AtrMYB72 regulates betalain biosynthesis by directly binding to the promoter of *AtrCYP76AD1*, thereby activating its transcription [149]. Exceptions to this pattern exist within Caryophyllales: in carnation (Caryophyllaceae), DcbHLH1 interacts with DcMYB1 and DcMYB2 to dynamically regulate petal pigmentation [147], reflecting the retention of MBW complex-mediated pigment regulation in certain lineages of this order (Table 2).

### Targeted flavonoid types

Expanding the scope to a broader range of horticultural plants uncovers remarkable mechanistic complexity. Even within the same functional subgroup, the flavonoid classes regulated can differ across species. For example, subgroups conventionally associated with proanthocyanidin biosynthesis in eudicots may also regulate anthocyanin biosynthesis in certain species, and their roles may extend beyond anthocyanins to other flavonoid types. While most subgroup members exhibit conserved functions across species, notable exceptions challenge this paradigm. For instance, *Arabidopsis* AtMYB5 (SGAtM5.1) specifically regulates proanthocyanidin biosynthesis in seeds [184], whereas grape VvMYB5a (SGAtM5.1) displays pleiotropic regulation of both anthocyanin and proanthocyanidin pathways in berries [79]. In kiwifruit, AcMYB123 (SG5) activates *AcANS* and *AcF3GT1* transcription, mediating spatiotemporal control of anthocyanin biosynthesis specifically in the inner pericarp [134]. Similarly, Peace, a TT2-like R2R3-MYB in SG5, has been shown to regulate anthocyanin biosynthesis, contributing to petal pigmentation in flowering peach (with both variegated and fully pigmented flowers) [103]. Notably, in *Arabidopsis*, the SG5 does not participate in regulating anthocyanin synthesis, highlighting functional divergence even within orthologous subgroups. These findings illustrate how evolutionary divergence drives lineage-specific functional shifts in orthologous MYBs, underscoring the intricate interplay between flavonoid regulatory networks and adaptive evolution. Such observations highlight the need for species-specific functional validation alongside phylogenetic analyses to decipher the evolutionary basis of horticultural traits.

## Case studies from models to horticultural crops: intra-species divergence after gene duplication

**Figure 5:**
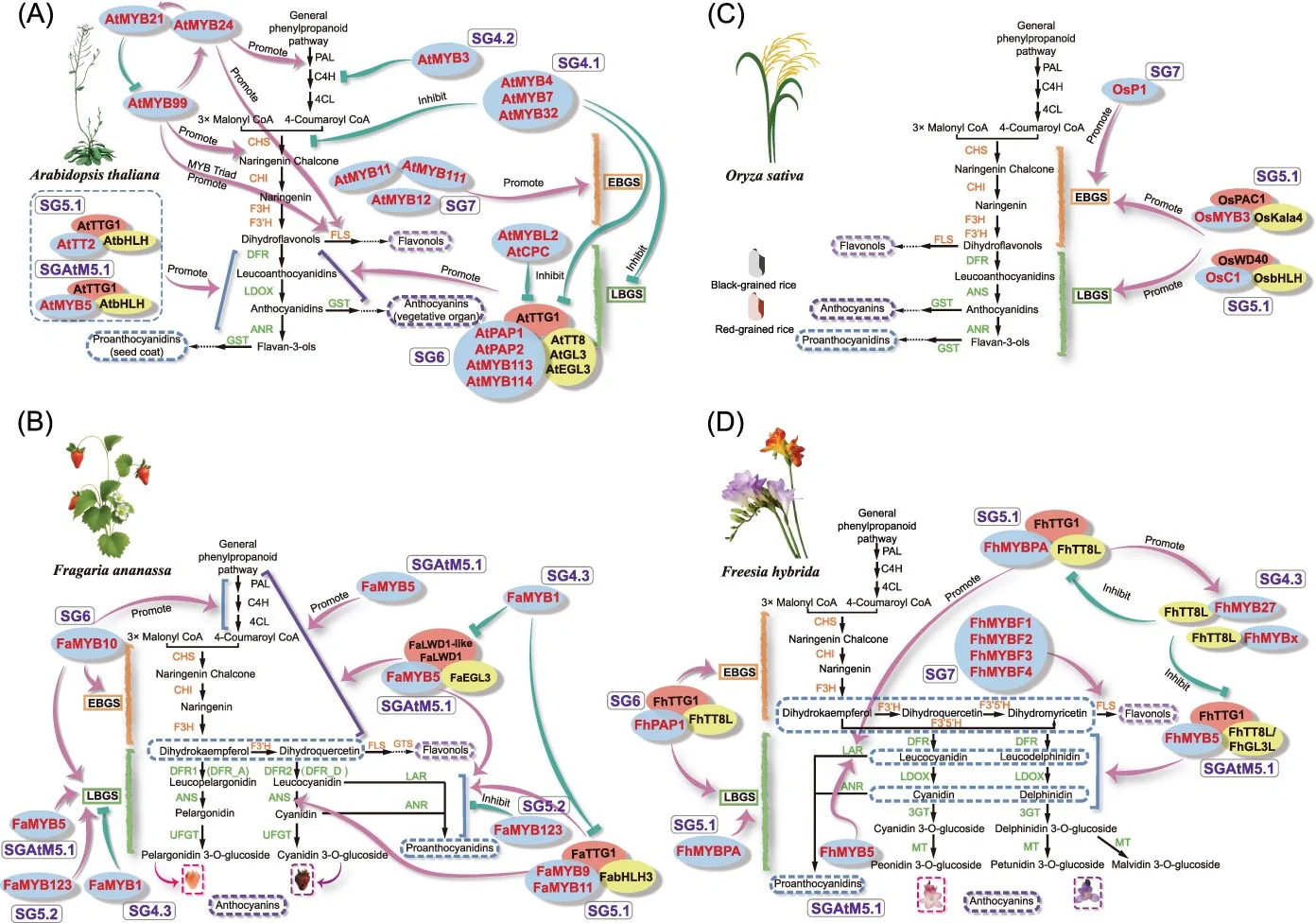
MYB-centered transcriptional regulatory networks of flavonoid biosynthesis in model plants and horticultural plants. (A) *A. thaliana*, (B) *F.* × *ananassa*, (C) *O. sativa*, and (D) *F. hybrida*. The core framework is based on the structural gene pathway of flavonoid regulation, with key transcriptional regulators (MYB-centered) annotated peripherally. Arrows depict intergenic regulatory networks: pink arrows indicate promotion, and teal arrows indicate inhibition. MYBs included in the regulatory network are highlighted in red font, annotated with their respective subgroups in purple font. The network integrates known regulatory interactions to demonstrate hierarchical control of flavonoid metabolism, emphasizing both conserved and lineage-specific regulatory modules across these representative species.

To gain deeper insights into intra-species functional divergence of flavonoid-regulatory MYBs following gene duplication, we selected model plants *A. thaliana* (eudicot, Brassicaceae) and *O. sativa* (monocot, Poaceae), alongside representative horticultural species *Fragaria* × *ananassa* (eudicot, Rosaceae) and *Freesia hybrida* (monocot, Iridaceae) (Fig. 5; Table 3). These species exemplify distinct evolutionary trajectories, ecological adaptations, and agronomic traits. Within a single species, these paralogous genes achieve precise regulation of different tissues and flavonoid branches through subfunctionalization or neofunctionalization.

In *Arabidopsis*, protoplast transient expression and T-DNA mutant analyses have demonstrated that SG7 genes (*AtMYB11*, *AtMYB12*, and *AtMYB111*) exhibit structural similarity and functional redundancy, and independently regulate the EBGs of flavonoid biosynthesis, including *CHS*, *CHI*, *F3H*, and *FLS*. During seedling development, these MYBs act additively via differential spatial expression: *AtMYB12* primarily drives flavonol biosynthesis in roots, whereas *AtMYB111* mediates cotyledon-specific flavonol production [152, 153]. AtPAP1, AtPAP2, AtMYB113, and AtMYB114 form MBW complexes with AtTTG1 and AtbHLH (e.g. AtTT8) proteins to activate LBGs for anthocyanin biosynthesis (e.g. *DFR*, *LDOX*), inducing deep purple pigmentation in organs [48, 154]. The MBW complex containing AtTT2 and AtMYB5 is essential for *ANR* expression, a core enzyme in seed coat proanthocyanidin biosynthesis [56, 155, 156]. Conversely, AtMYB4 interacts with AtTT8 in MBW complexes to repress transcriptional activity and suppress expression of MBW complex components (AtPAP1, AtPAP2, and AtTT2). AtMYB4 can also independently repress EBGs (e.g. *CHS*) [157]. AtMYB7 and AtMYB32, closely related to AtMYB4, function as repressors of distinct flavonoid metabolic branches, with *AtMYB7* expression repressed by AtMYB4 [13, 158]. EMSA and ChIP-qPCR analyses have shown that AtMYB3 negatively regulates the phenylpropanoid pathway by binding to the promoter of *C4H* [159]. Additionally, R3-MYB proteins AtMYBL2 and CPC act as repressors of anthocyanin biosynthesis: AtMYBL2 directly binds AtTT8 to form a complex that suppresses *DFR* expression, while AtCPC competes with AtPAP1/2 for bHLH binding (Fig. 5A) [163–165]. In addition to SG7 genes, SG19 members act as pollen-specific flavonol regulators via three mechanisms: first, independent activation, where AtMYB21 and AtMYB24 directly activate the flavonol biosynthetic gene *FLS1* [160, 161], and dual-luciferase and ChIP assays confirm AtMYB21 independently activates the *PAL4* promoter while AtMYB24 activates *PAL1/2* promoters; second, transcriptional cascade regulation, where promoter activation assays and mutant analyses clearly verify a closed-loop network—*AtMYB99* activates *AtMYB24*, which in turn activates *AtMYB21*, and *AtMYB21* negatively regulates *AtMYB99* to prevent excessive accumulation of individual factors and maintain expression homeostasis [161]; third, synergistic regulation via a MYB triad (AtMYB21/24/99), where protein–protein interactions enhance regulatory efficiency as AtMYB99 directly binds the *CHS* promoter, and the complex synergistically activates targets including *FLS1* [160, 161].

As a commercially pivotal fruit crop, strawberry relies on specialized MYB TFs to coordinate flavonoid biosynthesis during fruit ripening. FaMYB10 functions as a global regulator of the flavonoid/phenylpropanoid pathway, activating both EBGs and LBGs, including *PAL*, *C4H*, *4CL*, *CHS*, *CHI*, *F3H*, *DFR*, and *ANS*, with preferential control over EBGs such as *FaPAL* and *FaCHS* [166]. FaMYB123 promotes anthocyanin biosynthesis while suppressing proanthocyanidin accumulation. Genetic knockout of *FaMYB123* disrupts this balance, downregulating the anthocyanin-related gene *ANS* while upregulating proanthocyanidin pathway genes (*ANR*, a downstream gene of *ANS*/*LDOX*; *LAR*) [167]. This regulatory circuitry is critical for maintaining metabolic balance between anthocyanins and proanthocyanidins, safeguarding fruit flavor by preventing metabolic flux redirection toward proanthocyanidins. Such redirection could otherwise cause ectopic accumulation of condensed tannins in mature fruits, which are typically synthesized during the green fruit stage. Mechanistically, FaMYB10 and FaMYB123 exhibit a coordinated division of labor: FaMYB10 orchestrates broad pathway activation with primary control over EBG regulation, whereas FaMYB123 acts on LBGs (e.g. *ANS*), fine-tuning metabolic flux to maintain pigmentation and organoleptic balance. Transcriptomic analysis shows that *FaMYB5* overexpression in fruit significantly upregulates EBGs (*C4H*, *PAL*, *CHS*) and LBGs (*F3′H*, *ANS*, *LAR*). Functional validation confirms FaMYB5 positively regulates anthocyanin and proanthocyanidin accumulation by independently trans-activating *F3′H* and *LAR*. Additionally, FaEGL3 and FaLWD1/FaLWD1-like form an MBW complex with FaMYB5 to enhance regulatory efficiency toward downstream targeted genes (e.g. *F3′H*, *LAR*) [168]. FaMYB9 and FaMYB11 collaborate with FabHLH3 and FaTTG1 to form a ternary MBW complex, directly activating promoters of proanthocyanidin biosynthetic genes (*FaANS*, *FaANR*, *FaLAR*). This regulatory module redirects flavonoid metabolic flux from anthocyanins to proanthocyanidins, resulting in reduced anthocyanin and increased proanthocyanidin content in ripe fruits [170]. *FaMYB9* and *FaMYB11* are highly expressed during the early fruit development stage, coinciding with the peak accumulation of proanthocyanidin metabolites. During fruit ripening, proanthocyanidin content gradually declines, paralleled by a concurrent decrease in *FaMYB9*/*FaMYB11* expression. Conversely, FaMYB1 suppresses excessive anthocyanin and flavonol synthesis during late ripening to prevent cytotoxicity. FaMYB1 can directly repress *ANS* and *DFR* or competitively inhibit MBW complex formation by interacting with anthocyanin-regulatory bHLH proteins (Fig. 5B) [171]. In summary, the expansion and divergence of genes such as *FaMYB10* (SG6), *FaMYB123* (SG5.2), *FaMYB9/11* (SG5.1), *FaMYB5* (SGAtM5.1), and *FaMYB1* (SG4.3) within strawberry collectively form an intricate regulatory network. This network precisely modulates metabolic flux balance across different fruit developmental stages (proanthocyanidin accumulation in young fruits vs anthocyanin accumulation and suppression in mature fruits) and across distinct flavonoid branches (anthocyanins vs proanthocyanins vs flavonols). Notably, strawberries harbor two *DFR* isoforms with distinct substrate specificities. *DFR1* (*DFR_A* type) is highly selective for dihydrokaempferol (*DHK*) and does not accept dihydroquercetin (*DHQ*) or dihydromyricetin (*DHM*), while *DFR2* (*DFR_D* type) prefers *DHQ*/*DHM*, catalyzing only their conversion and completely excluding *DHK* [185]. Differences in their expression ratio, catalytic efficiency, and the loss of *F3′H* activity in late fruit development of cultivated strawberries collectively constitute an elaborate regulatory system. Shaped by natural selection, this system precisely modulates anthocyanin biosynthesis and fruit coloration. For example, it facilitates the accumulation of large amounts of pelargonidin-type pigments in cultivated strawberries. This non-classical *DFR1* (*DFR_A* type) with strict *DHK* specificity is rare across most plant lineages. Most angiosperms possess classical *DFR* types, including *DFR_N* (recognizing all three dihydroflavonols: *DHK*, *DHQ*, *DHM*) and *DFR_D* (preferring *DHQ*/*DHM*), which lack the stringent substrate specificity of strawberry *DFR* isoforms. Thus, the unique divergence of strawberry *DFR* isoforms (coexistence of *DFR_A* and *DFR_D*) is the core driver of its complex regulatory mechanism. Such a mechanism is presumably absent in plant lineages with only classical *DFR* types, as these lack the isoform-specific substrate partitioning and coordinated expression patterns observed in strawberries [185, 186].

To date, only a limited number of flavonoid-regulatory MYBs have been validated in rice (Fig. 5C). OsC1, belonging to SG5, directly interacts with OsbHLH and OsWD40 to form an MBW complex. This complex positively regulates the entire flavonoid pathway, particularly promoting the biosynthesis of anthocyanins and proanthocyanidins in rice grains and leaves. In contrast, OsP1 from SG7 independently and directly activates EBGs of flavonoid biosynthesis, including *CHS*, *CHI*, *F3′H*, and *FLS*, thus positively regulating flavonol synthesis [50, 172, 187–194].

In freesia, FhPAP1 forms an MBW complex with FhTT8L and FhTTG1 to specifically activate anthocyanin accumulation in petals [176]. When expressed alone, *FhMYB5* mildly activates LBGs (e.g. *DFR*, *LDOX*), but co-expression with bHLH factors (FhTT8L or FhGL3L) significantly enhances transcription of both EBGs and LBGs. The FhMYB5-FhTT8L/FhGL3L-FhTTG1 complex directly binds promoters of anthocyanin target genes (e.g. *FhDFR3*, *FhLDOX1*), potentiating their expression. Ectopic expression of *FhMYB5* in tobacco additionally activates *NtLAR*, promoting proanthocyanidin monomer accumulation [177]. Conversely, FhMYB27 and FhMYBx suppress anthocyanin synthesis by competitively binding bHLH proteins, disrupting MBW complex formation [178]. FhMYBPAs can directly bind promoters of proanthocyanidin genes (e.g. *FhDFR3*, *FhLAR*). They can also form MBW complexes with FhTT8L and FhTTG1 to co-activate proanthocyanidin pathways, and exert feedback regulation on the repressors FhMYB27 and FhMYBx to dampen their own activity [179]. Additionally, protoplast transient transfections, promoter truncations, and ChIP-qPCR analyses have shown that FhMYBF1/2/3/4 directly activate flavonol biosynthetic genes, thereby coordinately regulating flavonol metabolism (Fig. 5D) [180].

## Evolutionary drivers and molecular mechanisms underlying functional divergence

### Pollinator attraction

Plants rely on diverse pollination vectors (wind, water, insects, or birds) and are classified as anemophilous, hydrophilous, entomophilous, or ornithophilous based on their primary pollinators [195, 196]. For entomophilous (insect-pollinated) eudicots, anthocyanin-mediated floral pigmentation is a key adaptive trait for pollinator attraction [197]. Tandem duplication and subfunctionalization of SG6 R2R3–MYBs in core eudicots (e.g. Rosaceae, Asteraceae) enable precise regulation of anthocyanin patterning. For example, snapdragon *ROSEA* and *VENOSA* govern complex petal pigmentation to enhance pollinator specificity [123]; petunia *PhAN2* and homologs (*PhPHZ*, *PhDPL*) control both uniform coloration and discrete spot patterns that guide hawkmoth or bee visitation [49, 127]. In apple, *MYB10*-like paralogs exhibit distinct tissue specificity: some drive deep red fruit skin during maturation, others regulate petal blush in spring blossoms, and still others control red pigmentation in young leaves and shoot tips [109, 112, 114, 115].

By contrast, monocots, particularly anemophilous (wind-pollinated) Poaceae species, such as maize and rice, lack SG6 MYBs (Figs 2, 3; Supplementary Fig. S3), likely due to pseudogenization and subsequent gene loss [59]. This loss reflects reduced selection pressure for floral pigmentation, as their reproductive success relies on pollen dispersal rather than pollinator attraction [195, 196]. Maize and rice flowers are typically inconspicuous, with minimal floral anthocyanin accumulation, and our phylogenetic analyses suggest the absence of SG6 orthologs in Poaceae, indicating early gene loss or absence during monocot evolution. Compensatory mechanisms have evolved: SG5 MYBs (e.g. OsC1 in rice) regulate anthocyanin biosynthesis to support stress adaptation and crop yield, not floral display [50]. We hypothesize that the loss of SG6 and the evolution of wind pollination in Poaceae do not represent a unidirectional causal relationship but rather a coevolutionary process. Early Poaceae may have adapted to wind pollination by reducing floral pigments (lowering energy costs), weakening selection pressure on anthocyanin regulators like SG6 and ultimately leading to its loss. Conversely, SG6 loss further restricted floral anthocyanin synthesis, reinforcing wind pollination dependence. This bidirectional feedback is also reported in other wind-pollinated lineages (e.g. Cyperaceae) [196], representing a conserved adaptive pattern. SG5's failure to take over floral pigmentation in Poaceae is presumably attributed to two key factors. First, functional specialization: SG5’s core role encompasses both proanthocyanidin and anthocyanin synthesis, with expression mainly confined to vegetative organs (e.g. rice leaves) and seeds (Fig. 4C) and extremely low levels in floral organs, lacking tissue-specific modules to drive floral pigment accumulation. Second, absence of selection pressure: Poaceae flowers have simplified structures (without showy petals), and wind pollination does not require pigment-based pollinator attraction, preventing SG5 from undergoing flower-specific functional divergence and retaining its ancestral roles in vegetative organs and seeds. Notable exceptions exist in insect-pollinated ornamental monocots. In lily (Liliaceae), tandem duplicates LhMYB12 and LhMYB6 create contrasting tepal bands and spots that enhance pollinator attraction [53]. Similarly, freesia (Iridaceae) FhPAP1 reconstitutes eudicot-like MBW complexes to drive petal color diversity, demonstrating convergent retention of eudicot-like regulatory modules in certain monocot lineages [176].

### Abiotic stress adaptation

Flavonoid-mediated abiotic stress responses (UV protection, drought tolerance, and oxidative mitigation) underpin distinct MYB regulatory strategies in angiosperms. Monocots employ a ‘streamlined efficiency’ model: rice OsC1 (SG5) coordinates anthocyanin and proanthocyanidin biosynthesis in leaves and hulls through MBW complexes, enhancing UV tolerance and antioxidant capacity in open habitats [50]. This centralized regulation, enabled by SG5 retention and SG6 loss, minimizes metabolic latency but lacks tissue-specific fine-tuning. Eudicots exhibit a more ‘modular’ network comprising distinct R2R3-MYBs that specialize in activating EBGs, LBGs, or acting as repressors, with multiple R2R3-MYB subgroups (e.g. SG6, SG5, SG7, SGAtM5, SG4) governing specific flavonoid classes across tissues. For instance, *Arabidops*is AtMYB12, AtMYB11, and AtMYB111 (SG7) activate genes to accumulate flavonols in different tissues for UV protection, independently of MBW complexes [152]. Moreover, a negative feedback regulatory module, comprising the R3-MYB repressor MYBL2 and R2R3-MYB activator PAP1, fine-tunes high light-induced anthocyanin biosynthesis in *Arabidopsis* [198, 199]. This decentralized architecture allows precise spatiotemporal control of flavonoid profiles across varying environmental conditions, optimizing resource allocation during development.

### Domestication pressures

Artificial selection has profoundly reshaped MYB regulatory networks to meet agronomic needs. The evolution of rice anthocyanin pigmentation is highly complex, primarily due to Poaceae-specific regulatory patterns, with MYBs as central players [172]. As the ancestor of cultivated rice, *Oryza rufipogon* (common wild rice) typically accumulates leaf anthocyanins, confirming this as a primitive trait [172, 188]. Wild rice retains fully functional core regulators (e.g. OsC1), with a regulatory logic conserved in Poaceae (e.g. maize) that relies on the MBW complex to activate anthocyanin biosynthetic structural genes [172, 188, 191]. During domestication, anthocyanin accumulation diverged sharply, driven by MYB and related gene mutations: indica rice carries a 10-bp deletion in *OsC1* [172], while the aus subpopulation has a C → G SNP in *OsC1* [172]. Both artificially selected mutations eliminate leaf anthocyanins in most cultivated varieties. In contrast, black rice achieves ectopic high anthocyanin expression (especially in the pericarp) via a specific mechanism: the R2R3-MYB OsKala3 forms an MBW complex with bHLH OsKala4 [175], and tandem repeats in the *OsKala3* promoter positively correlate with pericarp anthocyanin levels, enabling fine regulation via MYB promoter structural variations [174, 175]. Additionally, OsC1 induces anthocyanin accumulation in specific tissues (calli, shoot apices, leaf sheaths) and enhances antioxidant capacity. Its overexpression in indica rice does not affect pericarp pigmentation, demonstrating tissue-specific regulation [50, 173].

Conversely, consumer demand for vivid appearance and high antioxidant content has fueled MYB expansions in fruit and vegetable crops. Tandem duplications and positive selection have enhanced the diversity of SG6 and SG5, boosting regulatory plasticity. For instance, duplication of VvMYBA1 and VvMYBA2 underlies the red–black skin of table grapes and the diversity of wine grape varieties [76]. Phylogenetic analyses reveal lineage-specific expansion of SG5 in Rosaceae: strawberry harbors three functionally validated SG5 genes (SG5.1: *FaMYB9/11*; SG5.2: *FaMYB123*) that orchestrate the metabolic balance between anthocyanins and proanthocyanidins (Fig. 3 and Supplementary Fig. S3). This regulatory complexity is absent in three other representative species (e.g. *Arabidopsis*), where the SG5 contains only one gene assigned to SG5.1. FaMYB123 (SG5.2) and FaMYB9/11 (SG5.1) fine-tune this balance to modulate fruit flavor (reducing astringency) and pigmentation, while FaMYB1 (SG4.3) acts as a late-ripening repressor, maintaining postharvest quality by preventing excessive anthocyanin accumulation [167, 170, 171, 200]. Since MYB10 (SG6) is the major driver of natural variation in strawberry skin and flesh color, breeders have exploited allelic variants of these MYBs to select for deeper/earlier coloration, bicolored fruits, and improved postharvest stability [201]. Ornamental breeders have similarly harnessed MYB diversity to generate novel floral patterns. Unusually among monocots, lily and tulip retain tandem-duplicated SG6 MYBs, enabling diverse floral pigmentation for ornamental value. In freesia, FhPAP1 and FhMYB5 employ a conserved MBW complex-mediated regulatory mode similar to that of eudicots to activate anthocyanin biosynthesis [176, 177]. This similarity stems from the retention and functional specialization of a conserved MBW regulatory module inherited from the common ancestor of monocots and eudicots [19, 202, 203]. Additionally, FhMYB5 uniquely activates *LAR* to promote proanthocyanidin monomers, while FhMYBF1/2/3/4 exhibit tissue-specific flavonol regulation, demonstrating lineage-specific innovations for floral color adaptation [180]. Notably, while rice exemplifies stress-yield adaptation, ornamental monocots like lily and freesia retain a demand for floral color evolution alongside core monocot traits, highlighting intra-monocot divergence [64].

During sugar beet domestication, anthocyanin regulatory factors were co-opted into the betalain pathway, with BvMYB1 as the core regulator. Derived from anthocyanin-associated MYB TFs (SG6), BvMYB1 neofunctionalized to directly activate betalain biosynthesis via key enzyme genes. Devoid of bHLH-interacting conserved residues, it is independent of the MBW complex. Human preference for red, high-betalain flesh drove core selection pressure on the functional Y allele of *BvMYB1*, which confers high gene expression and betalain accumulation. In contrast, varieties with a non-functional allele remain white. Domestication-selected ‘betalain-overproducing’ red beets thus fulfill human needs for pigments, nutrition, and stress tolerance [148].

### Structural determinants

The MYB–bHLH interaction domain is critical for complex formation, while the MYB DNA-binding domain dictates target gene recognition specificity. Studies on ZmR-dependent (ZmC1-mediated) and ZmR-independent (ZmP1-mediated) regulation in maize identified specific MYB domain residues enabling ZmC1-ZmR interaction [204]. Transferring six ZmC1 residues to ZmP1 conferred ZmR responsiveness while preserving ZmR-independent activity. Further research defined a conserved subgroup IIIf bHLH interaction motif ([DE]Lx₂[RK]x₃Lx_6_Lx₃R) critical for MYB–bHLH complex specificity and stability [205, 206]. Consistent with these conserved motifs, a comprehensive analysis of Caryophyllales species revealed that residues within the R3 repeat of PAP1-like MYBs, including positions 5, 6, 9, 10, 13, 14, and 24, are pivotal for mediating MYB–bHLH interactions. Convergent substitutions in these residues were observed in betalain-pigmented lineages, leading to impaired MBW complex assembly and functional degeneration of anthocyanin biosynthesis regulation [182]. This aligns with observations in banana R2R3-MYBs (MaMYBA1, MaMYBA2, MaMYBPA2), which exhibit stronger anthocyanin activation potential when interacting with the monocot bHLH ZmR than with the eudicot bHLH AtEGL3 [68]. Importantly, such interaction interface differences likely represent a key molecular basis for divergent MBW complex dependencies among distinct MYBs (e.g. SG6 vs SG5). Although the R2R3 DNA-binding domain is highly conserved, the variable C-terminal region likely harbors clade-specific motifs influencing co-regulator interactions (e.g. with bHLHs) and target gene activation. Despite low conservation in non-MYB regions, closely related subgroup members often share short conserved C-terminal motifs, such as the EAR motif (‘pdLNL(D/E)L’ or ‘DLNxxP’ in SG4) [207], ‘(R/K)PRPRx(F/L)’ motif in SG6, the SID motif, and the TLLLFR motif. These motifs indicate common evolutionary origins and functional or structural conservation [13, 208–210]. Specifically, future studies should focus on C-terminal structural characteristics of members from different flavonoid-regulatory subgroups, particularly functionally divergent members within the same subgroup.

Diverse evolutionary drivers and molecular mechanisms have collectively shaped MYB regulatory network divergence between monocots (especially Poaceae) and eudicots. These divergences define flavonoid profiles, including the relative abundance and tissue-specific distribution of anthocyanins, proanthocyanidins, and flavonols, ultimately shaping key horticultural traits: flower/fruit color, flavor, stress resilience, and nutritional value. However, extrapolating absolute divergence rules between the two lineages requires caution, even though our review has systematically summarized the representative available data from model and major horticultural species. Multiple factors may bias current conclusions: first, the narrow scope of studied species (e.g. underrepresented non-Poaceae monocots like Arecaceae/Zingiberaceae, basal eudicots, such as Ranunculales, and wild crop relatives with adaptive flavonoid traits); second, variations in experimental systems or genetic backgrounds, plus overrepresentation of model plants and major horticultural crops, which may highlight non-universal lineage-specific patterns. Future studies should prioritize expanded comparative analyses across diverse species to validate these divergence patterns' generalizability and refine our understanding of MYB-mediated flavonoid regulation in horticultural plants.

## Harnessing evolutionary and functional divergence for targeted trait engineering

This review systematically synthesizes and compares the current understanding of functional divergence among flavonoid-regulatory MYB TFs, with a particular focus on R2R3–MYBs within key flavonoid-regulatory subgroups across monocot (particularly Poaceae) and eudicot horticultural plants. We highlight that this divergence manifests at three distinct levels: inter-subgroups, cross-species, and intra-species. In addition, we elucidate the key roles of ecological pressures, domestication processes, and structural determinants in shaping these patterns of divergence. We also discuss the potential horticultural applications of this knowledge and propose future directions for related research.

The conserved core functions of MYB TFs, combined with their lineage-specific adaptive features, may potentially facilitate the transfer and optimization of regulatory modules between monocots and eudicots. For example, introducing eudicot SG6 MYB activators (e.g. grape VvMYBA1, apple MdMYB10) into monocots such as rice or maize might help to bypass certain inherent limitations associated with SG6 loss in these species, which could in turn promote anthocyanin accumulation in grains to potentially improve nutritional value and visual appeal. That said, this strategy would likely require the concurrent transfer of their cognate bHLH partner proteins to achieve functional outcomes. Conversely, engineering key components of the relatively streamlined and efficient monocot MBW complexes (e.g. specific protein interaction domains from OsC1 or ZmC1) into eudicots could potentially strengthen the coordinated activation of both EBGs and LBGs in the flavonoid pathway, which may offer a viable approach to enhance stress tolerance in horticultural crops such as tomato or strawberry.

CRISPR/Cas9 and base-editing technologies represent promising tools that could enable precise modification of MYB function by targeting key structural motifs or regulatory regions. Targeted modification of EAR motifs in R3-MYB repressors (e.g. strawberry FaMYB1, lily LhR3MYB1) might help to alleviate the repression of the flavonoid biosynthetic pathway, with the potential to enhance anthocyanin or flavonol accumulation while potentially reducing the risk of undesirable pleiotropic effects. In addition, targeted editing of the promoter regions of SG6 or SG5 MYBs to introduce tissue-specific cis-acting elements (e.g. fruit- or petal-specific enhancers) could help to restrict flavonoid synthesis to target plant organs, which may contribute to reducing unnecessary energy expenditure in non-essential tissues.

Domestication bottlenecks have frequently led to the loss of beneficial MYB alleles in cultivated crop varieties, but pangenome analyses of wild relatives provide a potential strategy for recovering such alleles to restore valuable traits. Wild relatives of strawberry or apple may harbor SG6 or SG5 alleles associated with more vibrant fruit pigmentation or higher proanthocyanidin content, and these alleles could be reintroduced into elite cultivars through marker-assisted breeding approaches. Extremophile relatives (e.g. drought-tolerant wild rice, UV-resistant desert plants) are also likely to possess stress-responsive MYB alleles that enhance flavonoid-mediated stress resilience, and the transfer of these alleles could potentially improve crop performance under the constraints of climate change. Haplotype analysis of MYB genes across diverse germplasm resources, combined with genome-wide association studies (GWAS), can help identify natural variants linked to desirable agronomic traits, thereby enabling marker-assisted selection to accelerate crop breeding processes. This integrated approach has already been successfully applied to key horticultural crops including eggplant and citrus [211].

One key challenge in flavonoid trait engineering is achieving a balance in the distribution of metabolic flux across the interconnected branches of the flavonoid biosynthetic pathway (i.e. anthocyanins, proanthocyanidins, flavonols, flavones, and other subclasses), as these pathways compete for common precursors such as dihydroflavonols. Disrupting this balance induces undesirable traits: excessive proanthocyanidin accumulation causes astringency in ripe fruits (e.g. unripe persimmons, a rich source of proanthocyanidins), while reduced flavonol levels impair UV protection and stress resilience. Precision metabolic engineering thus requires targeted modulation of branch-specific regulators to redirect flux without compromising plant fitness or product quality, and the interplay between activating and repressing MYBs provides a potential molecular switch for this balance. In strawberry, FaMYB123 (SG5.2) activates anthocyanin biosynthesis while repressing proanthocyanidin biosynthetic genes (*ANR*, *LAR*), whereas FaMYB9/11 (SG5.1) promote proanthocyanidin accumulation during early fruit development. Co-expression of *FaMYB123* with a hypomorphic *FaMYB9* mutant could potentially reduce astringency in ripe berries by limiting the persistence of proanthocyanidins, while preserving anthocyanin-derived pigmentation. Similarly, in grape, targeting the balance between VvMYBA1 (anthocyanin activator) and VvMYBPA1 (proanthocyanidin activator) via promoter editing by enhancing *VvMYBA1* expression in ripe berries while repressing *VvMYBPA1* might enhance berry pigmentation without increasing the tannin accumulation. CRISPR-based tools enable more nuanced control of these pathways: base editing of FaMYB1 (SG4.3), a late-ripening repressor of anthocyanin synthesis, could attenuate its repressive activity to enhance fruit pigmentation, while potentially avoiding ectopic proanthocyanidin synthesis. In tomato, modifying the cis-regulatory elements of SG6 MYBs to restrict their expression to fruit peels, thus preventing flavonoid accumulation in leaves, could help direct flavonoid metabolic flux toward plant tissues relevant to consumers, optimizing resource allocation within the plant. Additionally, flavones, a category not addressed in earlier sections, play crucial roles in representative Apiaceae crops, such as carrot [212], and constitute a distinct competing branch in the flavonoid biosynthesis pathway, alongside the previously characterized anthocyanin and flavonol pathways. Furthermore, elucidating the roles of MYBs that co-regulate flavonoid and volatile organic compound (VOC) biosynthesis pathways, for instance, those linking anthocyanin accumulation to aroma compound production, opens up potential new avenues for the simultaneous enhancement of pigmentation, flavor, and fragrance in fruits and flowers via targeted MYB editing or controlled overexpression strategies [41].

## Concluding remarks and key challenges

Research on MYB-mediated regulation of flavonoid biosynthesis has emerged as a dynamic frontier in horticultural and plant sciences. Despite significant advances in deciphering this regulatory network in horticultural plants, critical gaps and challenges persist.

A primary challenge lies in unraveling the molecular determinants of functional divergence within key flavonoid-regulatory subgroups, which manifests in two key aspects. First, it involves deciphering the function of the variable C-terminal regulatory domains of R2R3–MYBs, regions harboring clade-specific motifs (e.g. activation or repression domains) that determine regulatory specificity. Equally critical is elucidating how structural differences in MYB–bHLH interaction domains modulate the assembly and functional activity of MBW complexes. Addressing these questions requires experiments including functional interaction assays; the core question is not merely whether two proteins interact, but whether their interaction retains the capacity to activate transcription at target gene promoters. Despite high sequence similarity, MYB paralogs (e.g. strawberry FaMYB10/FaMYB5) exhibit distinct target gene recognition, necessitating high-resolution binding analyses, such as ChIP-seq. Additionally, WRKY proteins (e.g. *Arabidopsis* TTG2, *Petunia* PH3) have been reported to physically interact with core MBW subunits, forming modified transcriptional complexes (e.g. MBWW quartets) that refine target specificity [213]. We therefore advocate for integrating the regulatory roles of WRKYs and other associated components into future studies.

While extensive studies have revealed that flavonoid regulatory mechanisms (largely driven by MYB variations) are generally well conserved across plants, and cross-species comparative analyses have provided valuable insights into interspecific pigmentation differences and evolutionary patterns [21], targeted investigations remain necessary to resolve the lineage-specific regulatory nuances within this broadly conserved framework. Such efforts are particularly critical given the current underrepresentation of key evolutionary lineages in functional studies, including monocots (e.g. gingers, palms, non-Poaceae grasses) and basal eudicots (e.g. Ranunculales), as well as their wild relatives. Beyond the core MYBs, our understanding of the full regulatory complexity of flavonoid pathways remains far from comprehensive, particularly regarding the co-evolution of MYBs with their bHLH/WD40 partners and other TF families (e.g. WRKY, NAC). For instance, how have bHLH proteins co-adapted with MYBs to maintain the functional integrity of MBW complexes across monocots and eudicots? Additionally, how environmental and hormonal signals (e.g. light, ABA, JA) are integrated into MYB-mediated regulatory networks remains poorly understood from an evolutionary perspective.

In conclusion, the evolutionary and functional divergence of MYB regulatory networks across Poaceae, non-Poaceae monocots, and eudicots provides a blueprint for precision horticultural breeding. Integrating evolutionary insights with advanced technologies will facilitate the engineering of horticultural crops with enhanced color, flavor, stress tolerance, and nutritional value, aligning with consumer preferences and agronomic requirements. The next decade of research is poised to bridge existing gaps, translating fundamental knowledge of MYB evolution into tangible agricultural innovations.

## Materials and methods

### Collection and identification of MYBs

Sequences for Fig. 2 were retrieved from public datasets cited in the literature. For Fig. 3, proteome files of 44 horticultural species were downloaded to establish a local database. BUSCO analysis was performed to evaluate genome assembly quality, with detailed results in Supplementary Table S1. Local HMM searches (HMMER) identified proteins containing the MYB domain (PF00249). Candidate R2R3-MYB proteins were filtered by three criteria: (i) exactly two MYB domains, (ii) minimum bit score of 35 for domain matching, and (iii) MYB domains separated by <20 amino acids. Redundant sequences and low-quality sequences (<100 amino acids) were removed. For Supplementary Fig. S2, all R2R3-MYB proteins from *M. polymorpha*, *S. moellendorffii*, *C. panzhihuaensis*, *A. trichopoda*, and *A. thaliana* were included. To infer their evolutionary origins, additional proteins from the key flavonoid-regulatory subgroups of selected horticultural species in Fig. 3 were also integrated.

### Multiple sequence alignment and phylogenetic analysis

Full-length amino acid sequences of identified MYBs were aligned using MAFFT with the --auto parameter. Alignments were trimmed with trimAl (-gt 0.15). For Fig. 2 and Supplementary Fig. S1, ML phylogenetic trees were constructed using IQ-TREE 2. ModelFinder implemented in IQ-TREE 2 was used to select the best-fitting substitution model based on the Bayesian Information Criterion (BIC). Branch support was assessed with 1000 bootstrap replicates. Trees were visualized and edited using ChiPlot, with bootstrap values ≥70% labeled on the branches. This threshold reflects moderate-to-strong branch support, a widely accepted standard for distinguishing reliable clades while balancing stringency and biological relevance [214]. AtCDC5 was chosen as the outgroup for rooting, as it encodes a conserved MYB-related protein evolutionarily distant from R2R3–MYBs, ensuring accurate tree rooting [215]. For Supplementary Fig. S2, ML phylogenetic trees were built with FastTree. For Fig. 3 and Supplementary Fig. S3, ML phylogenetic trees were constructed using both IQ-TREE 2 and FastTree. For large-scale classification of R2R3–MYB genes across 44 horticultural species, a stepwise phylogenetic tree construction strategy was employed: (i) all retrieved protein sequences were included for initial tree inference; (ii) sequences were further split based on whether they formed angiosperm-specific monophyletic clades; (iii) additional phylogenetic analyses were performed for each subclade independently. This approach ensures monophyly for each subgroup, which contains orthologous genes spanning from early-diverging angiosperms to derived eudicots and monocots. Using this strategy, we established a novel classification system for R2R3-MYBs within the key flavonoid-regulatory subgroups of angiosperms. Subsequently, phylogenetic trees for the 11 subclades were constructed using orthogroup members from 44 species, with *A. trichopoda*, *V. cruziana*, waterlily and *Euryale ferox* designated as sister lineages to all other angiosperms.

### Expression profile analysis

Standardized expression data for *A. thaliana* and *O. sativa* were downloaded from the Plant Public RNA-seq Database (https://www.plantrnadb.com/). Data for *S. lycopersicum* and *M. acuminata* were retrieved from Gene Expression Regulation Database of Horticultural plants (https://dphdatabase.com/). Detailed FPKM values are provided in Supplementary Tables S2–S5. Expression heatmaps were generated with TBtools after log₂(FPKM+1) transformation. Tissue-specific expression was quantified using the Tau metric (range: 0 = ubiquitous to 1 = highly tissue-specific), calculated from quantile-normalized log₂(FPKM+1) values averaged across biological replicates [216].

## Supplementary Material

Web_Material_uhag169

## Data Availability

All data supporting the findings of this study are included in the supplementary materials.
