# Supplementary material for: Evolutionary and functional divergence of MYB regulatory networks in flavonoid biosynthesis across Poaceae, non-Poaceae monocots, and eudicot horticultural plants
Source: Hortic Res. 2026 Apr 27;13(9):uhag169. doi: 10.1093/hr/uhag169 (PMC13533577; doi:10.1093/hr/uhag169)
Supplement: Supplementary file 1 [file web_material_uhag169.zip › 20260310FigS123.pdf]

Horticulture Research **Supplementary materials**

Article title: Evolutionary and functional divergence of MYB regulatory networks in flavonoid biosynthesis across Poaceae, non-Poaceae monocots, and eudicot horticultural plants

The following **Supplementary materials** is available for this article:

Fig. S1 Complete rooted phylogenetic tree of R2R3-MYBs from *Arabidopsis* and functionally characterized flavonoid-related R2R3-MYBs in horticultural plants. All subgroups are labeled adjacent to their respective gene clusters. Subgroups specifically involved in flavonoid regulation are highlighted in red. Bootstrap values are indicated at the nodes/branches.

Fig. S2 ML phylogenetic tree illustrating the evolutionary origins of the six functional groups within key flavonoid-regulatory subgroups. The analysis includes the complete R2R3-MYB protein complements from *Marchantia polymorpha*, *Selaginella moellendorffii*, *Physcomitrium patens*, *Cycas panzhihuaensis*, *Amborella trichopoda*, and *A. thaliana*. Key flavonoid-regulatory sequences from representative horticultural species (consistent with Fig. 3) were integrated to provide robust phylogenetic support for inferring ancestral relationships and lineage diversification.

Fig. S3 ML phylogenetic trees of key flavonoid-regulatory R2R3-MYB subgroups across 44 horticultural species. Bootstrap values are displayed on the branches. Different plant lineages are distinguished by distinct branch colors. Functionally validated genes (as referenced in Fig. 2) are highlighted in purple. Inferred gene expansion and loss events are annotated to the right of each tree. (a) SG6; (b) SG5.1; (c) SG5.2; (d) SG15.1/SG15.2; (e) SG7; (f) SGAtM5.1; (g) SGAtM5.2; (h) SG4.1; (i) SG4.2; (j) SG4.3; (k) Comprehensive tree of all key flavonoid-regulatory subgroups

constructed using IQ-TREE; (l) Comprehensive tree of all key flavonoid-regulatory subgroups constructed using FastTree.

Table S1 List of surveyed horticultural species, including data sources, BUSCO assessment results, and R2R3-MYB gene counts within each subclade of key flavonoid-regulatory subgroups.

Table S2 Gene FPKM values of R2R3-MYB genes from key flavonoid-regulatory subgroups in *A. thaliana*. This table lists genes and their corresponding expression levels across various tissues or conditions.

Table S3 Gene FPKM values of R2R3-MYB genes from key flavonoid-regulatory subgroups in *S. lycopersicum*. This table lists genes and their corresponding expression levels across various tissues or conditions.

Table S4 Gene FPKM values of R2R3-MYB genes from key flavonoid-regulatory subgroups in *O. sativa*. This table lists genes and their corresponding expression levels across various tissues or conditions.

Table S5 Gene FPKM values of R2R3-MYB genes from key flavonoid-regulatory subgroups in *M. acuminata*. This table lists genes and their corresponding expression levels across various tissues or conditions.

Table S6 Average expression levels and tissue specificity (tau) values of R2R3-MYB genes from key flavonoid-regulatory subgroups across various tissues of *A. thaliana*.

Table S7 Average expression levels and tissue specificity (tau) values of R2R3-MYB genes from key flavonoid-regulatory subgroups across various tissues of *S. lycopersicum*.

Table S8 Average expression levels and tissue specificity (tau) values of R2R3-MYB genes from key flavonoid-regulatory subgroups across various tissues of *O. sativa*.

Table S9 Average expression levels and tissue specificity (tau) values of R2R3-MYB

genes from key flavonoid-regulatory subgroups across various tissues of *M. acuminata*.

Data S1 Supporting data for Fig. 2, including the protein sequences used for phylogenetic tree construction, the multiple sequence alignment file, the trimmed alignment file, and the phylogenetic tree source file.

Data S2 Supporting data for Fig. S2, including the protein sequences used for phylogenetic tree construction, the multiple sequence alignment file, the trimmed alignment file, and the phylogenetic tree source file.

Data S3 Supporting data for Fig. 3 and Fig. S3, including R2R3-MYB protein sequences from key flavonoid-regulatory subgroups across 44 horticultural species (used for the phylogenetic tree construction of Fig. 3 and Fig. S3) and the corresponding multiple sequence alignment file.

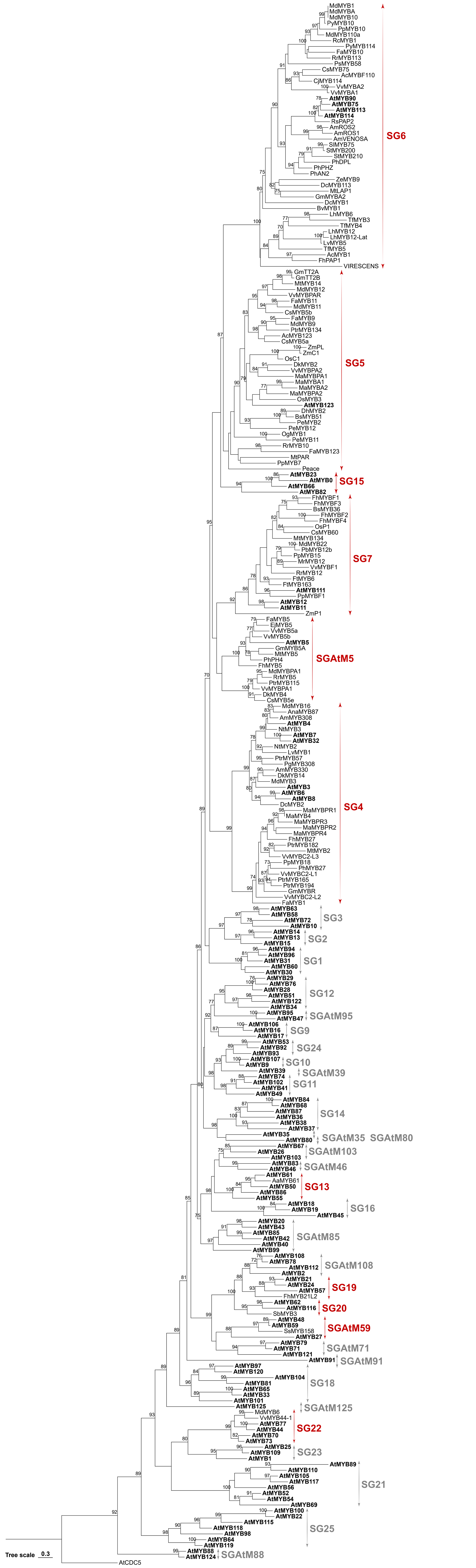

MdMYB1
MdMYBA
MdMYB10
PyMYB10
PpMYB10
MdMYB110a
RcMYB1
PyMYB114
FaMYB10
RrMYB113
PsMYB58
CsMYB75
AcMYBF110
CjMYB114
VvMYBA2
VvMYBA1
AtMYB90
AtMYB75
AtMYB113
AtMYB114
RsPAP2
AmROS2
AmROS1
AmVENOSA
SlMYB75
StMYB200
StMYB210
PhDPL
PhPHZ
PhAN2
ZeMYB9
DcMYB113
MtLAP1
GmMYBA2
DcMYB1
BvMYB1
LhMYB6
TfMYB3
TfMYB4
LhMYB12
LhMYB12-Lat
LvMYB5
TfMYB5
AcMYB1
FhPAP1
VIRESCENS
SG6
GmTT2A
GmTT2B
MtMYB14
MdMYB12
VvMYBPAR
FaMYB11
MdMYB11
CsMYB5b
FaMYB9
MdMYB9
PtrMYB134
AcMYB123
CsMYB5a
ZmPL
ZmC1
OsC1
DkMYB2
VvMYBPA2
MaMYBPA1
MaMYBA1
MaMYBA2
MaMYBPA2
OsMYB3
AtMYB123
DhMYB2
BsMYB51
PeMYB2
PeMYB12
OgMYB1
PeMYB11
RrMYB10
FaMYB123
MtPAR
PpMYB7
Peace
SG5
AtMYB23
AtMYB0
AtMYB66
AtMYB82
SG15
FhMYBF1
FhMYBF3
BsMYB36
FhMYBF2
FhMYBF4
OsP1
CsMYB60
MtMYB134
MdMYB22
PbMYB12b
PpMYB15
MrMYB12
VvMYBF1
RrMYB12
FtMYB6
FtMYB163
AtMYB111
PpMYBF1
AtMYB12
AtMYB11
ZmP1
SG7
FaMYB5
EjMYB5
VvMYB5a
VvMYB5b
AtMYB5
GmMYB5A
MtMYB5
PhPH4
FhMYB5
MdMYBPA1
RrMYB5
PtrMYB115
VvMYBPA1
DkMYB4
CsMYB5e
SGAtM5
MdMYB16
AnaMYB87
AmMYB308
AtMYB4
NtMYB3
AtMYB7
AtMYB32
NtMYB2
LvMYB1
PtrMYB57
PgMYB308
AmMYB330
DkMYB14
MdMYB3
AtMYB3
AtMYB6
AtMYB8
DcMYB2
MaMYBPR1
MaMYB4
MaMYBPR3
MaMYBPR2
MaMYBPR4
FhMYB27
PtrMYB182
MtMYB2
VvMYBC2-L3
PpMYB18
PhMYB27
VvMYBC2-L1
PtrMYB165
PtrMYB194
GmMYBR
VvMYBC2-L2
FaMYB1
SG4
AtMYB63
AtMYB58
AtMYB72
AtMYB10
SG3
AtMYB14
AtMYB13
AtMYB15
SG2
AtMYB94
AtMYB96
AtMYB31
AtMYB60
AtMYB30
SG1
AtMYB29
AtMYB76
AtMYB28
AtMYB51
AtMYB122
AtMYB34
SG12
AtMYB95
AtMYB47
SGAtM95
AtMYB106
AtMYB16
AtMYB17
SG9
AtMYB53
AtMYB92
AtMYB93
SG24
AtMYB107
AtMYB9
SG10
AtMYB39
SGAtM39
AtMYB74
AtMYB102
AtMYB41
AtMYB49
SG11
AtMYB84
AtMYB68
AtMYB87
AtMYB36
AtMYB38
AtMYB37
SG14
AtMYB35
AtMYB80
SGAtM35 SGAtM80
AtMYB67
AtMYB26
AtMYB103
SGAtM103
AtMYB83
AtMYB46
SGAtM46
AtMYB61
AaMYB61
AtMYB50
AtMYB86
AtMYB55
SG13
AtMYB18
AtMYB19
AtMYB45
SG16
AtMYB20
AtMYB43
AtMYB85
AtMYB42
AtMYB40
AtMYB99
SGAtM85
AtMYB108
AtMYB78
AtMYB112
AtMYB2
SGAtM108
AtMYB21
AtMYB24
AtMYB57
FhMYB21L2
SG19
AtMYB62
AtMYB116
SbMYB3
SG20
AtMYB48
AtMYB59
SsMYB158
AtMYB27
SGAtM59
AtMYB79
AtMYB71
AtMYB121
SGAtM71
AtMYB91
SGAtM91
AtMYB97
AtMYB120
AtMYB104
AtMYB81
AtMYB65
AtMYB33
AtMYB101
SG18
AtMYB125
SGAtM125
MdMYB6
VvMYB44-1
AtMYB77
AtMYB44
AtMYB70
AtMYB73
SG22
AtMYB25
AtMYB109
AtMYB1
SG23
AtMYB89
AtMYB110
AtMYB105
AtMYB117
AtMYB56
AtMYB52
AtMYB54
AtMYB69
SG21
AtMYB100
AtMYB22
AtMYB115
AtMYB118
AtMYB98
AtMYB64
AtMYB119
SG25
AtMYB88
AtMYB124
SGAtM88
AtCDC5
Tree scale 0.3

Fig. S1 Complete rooted phylogenetic tree of R2R3-MYBs from *Arabidopsis* and functionally characterized flavonoid-related R2R3-MYBs in horticultural plants. All subgroups are labeled adjacent to their respective gene clusters. Subgroups specifically involved in flavonoid regulation are highlighted in red. Bootstrap values are indicated at the nodes/branches.

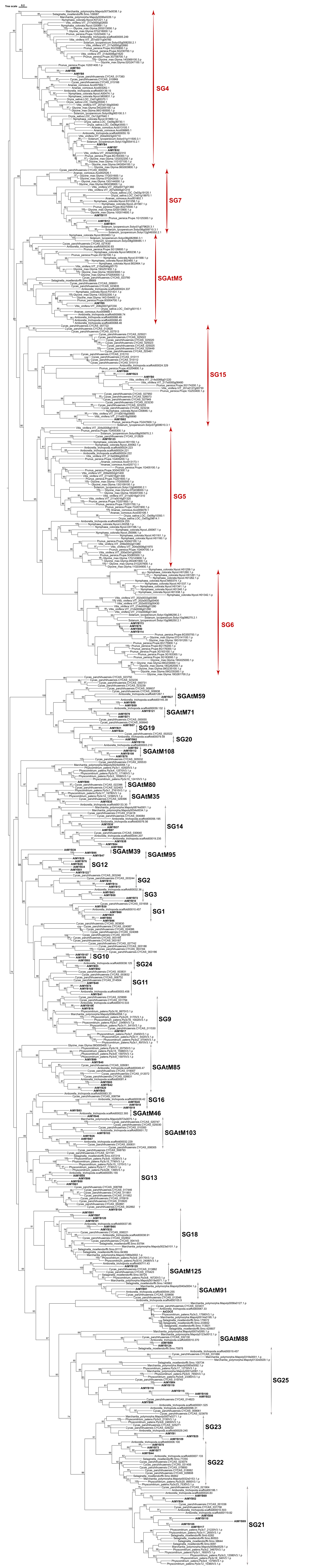
Tree scale
0.1
SG4
SG7
SGAtM5
SG15
SG5
SG6
SGAtM59
SGAtM71
SG19
SG20
SGAtM108
SGAtM80
SGAtM35
SG14
SGAtM39
SGAtM95
SG12
SG2
SG3
SG1
SG10
SG24
SG11
SG9
SGAtM85
SG16
SGAtM46
SGAtM103
SG13
SG18
SGAtM125
SGAtM91
SGAtM88
SG25
SG23
SG22
SG21

Fig. S2 ML phylogenetic tree illustrating the evolutionary origins of the six functional groups within key flavonoid-regulatory subgroups. The analysis includes the complete R2R3-MYB protein complements from *Marchantia polymorpha*, *Selaginella moellendorffii*, *Physcomitrium patens*, *Cycas panzhihuaensis*, *Amborella trichopoda*, and *A. thaliana*. Key flavonoid-regulatory sequences from representative horticultural species (consistent with Fig. 3) were integrated to provide robust phylogenetic support for inferring ancestral relationships and lineage diversification.

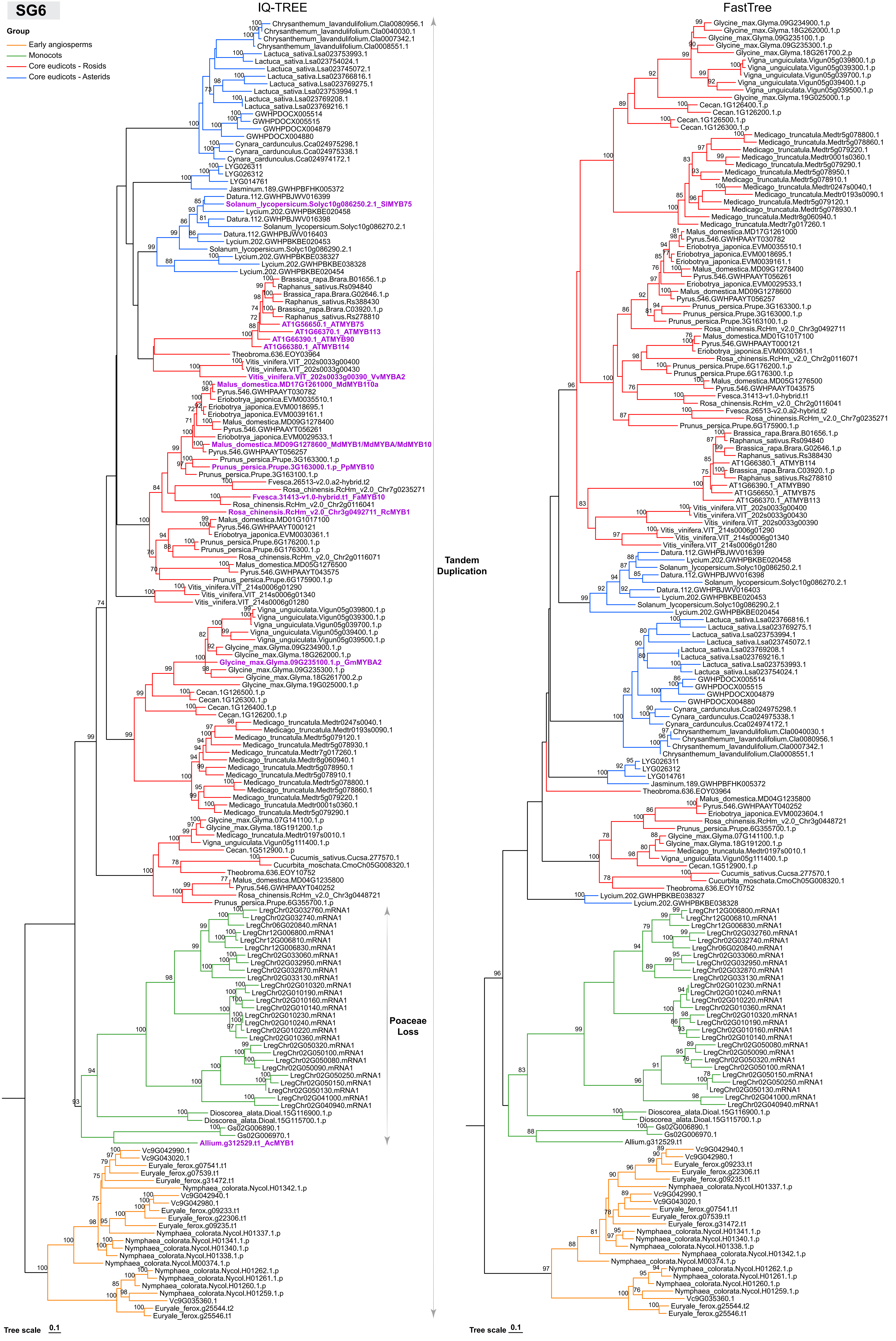

SG6
IQ-TREE
FastTree
Group
Early angiosperms
Monocots
Core eudicots - Rosids
Core eudicots - Asterids
Solanum_lycopersicum.Solyc10g086250.2.1_SlMYB75
AT1G56650.1_ATMYB75
AT1G66370.1_ATMYB113
AT1G66390.1_ATMYB90
AT1G66380.1_ATMYB114
Vitis_vinifera.VIT_202s0033g00390_VvMYBA2
Malus_domestica.MD17G1261000_MdMYB110a
Malus_domestica.MD09G1278600_MdMYB1/MdMYBA/MdMYB10
Prunus_persica.Prupe.3G163000.1.p_PpMYB10
Fvesca.31413-v1.0-hybrid.t1_FaMYB10
Rosa_chinensis.RcHm_v2.0_Chr3g0492711_RcMYB1
Glycine_max.Glyma.09G235100.1.p_GmMYBA2
Allium.g312529.t1_AcMYB1
Tandem Duplication
Poaceae Loss
Tree scale 0.1
Tree scale 0.1

SG5.1

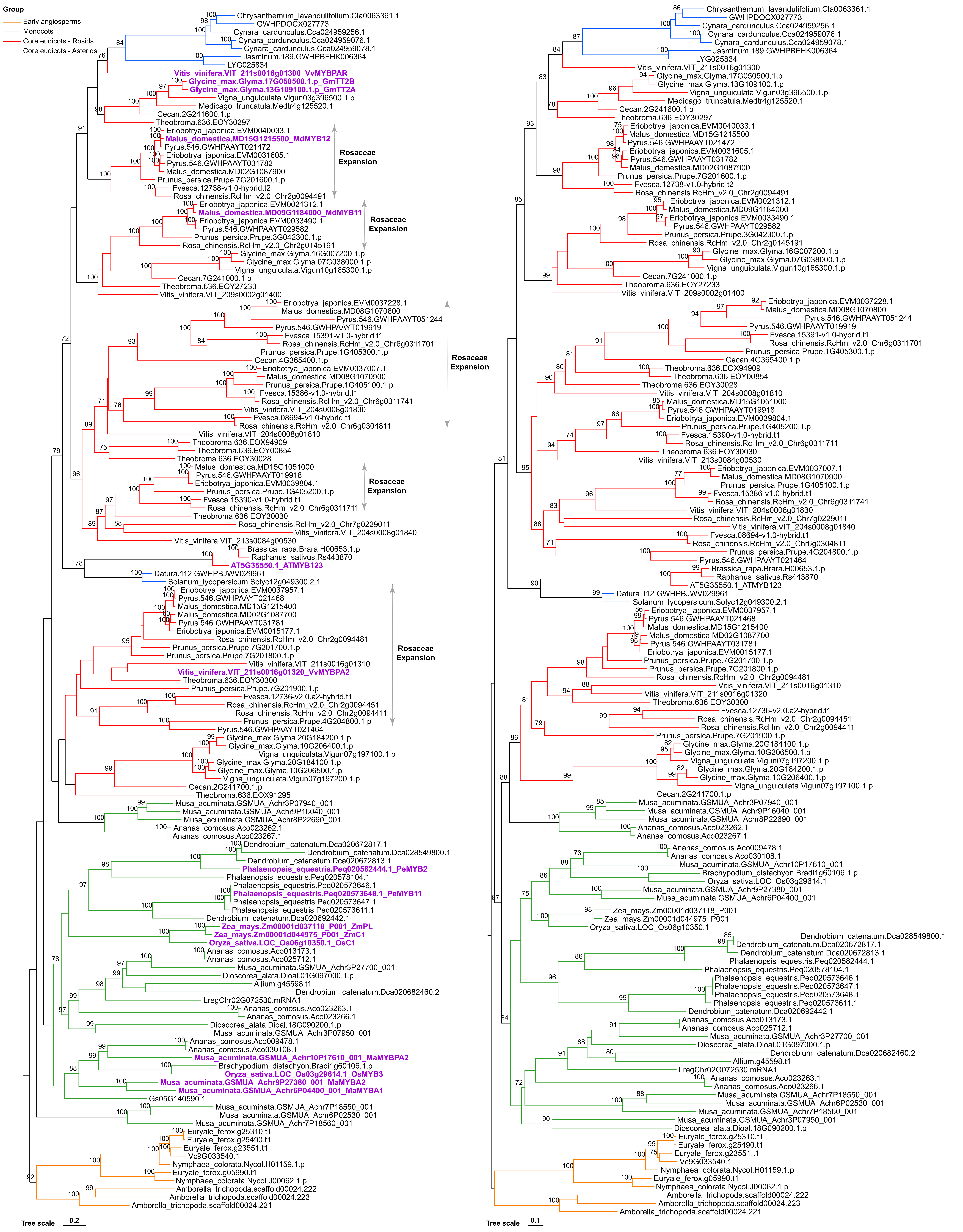
IQ-TREE
FastTree
Group
Early angiosperms
Monocots
Core eudicots - Rosids
Core eudicots - Asterids
Chrysanthemum_lavandulifolium.Cla0063361.1
GWHPDOCX027773
Cynara_cardunculus.Cca024959256.1
Cynara_cardunculus.Cca024959076.1
Cynara_cardunculus.Cca024959078.1
Jasminum.189.GWHPBFHK006364
LYG025834
Vitis_vinifera.VIT_211s0016g01300_VvMYBPAR
Glycine_max.Glyma.17G050500.1.p_GmTT2B
Glycine_max.Glyma.13G109100.1.p_GmTT2A
Vigna_unguiculata.Vigun03g396500.1.p
Medicago_truncatula.Medtr4g125520.1
Cecan.2G241600.1.p
Theobroma.636.EOY30297
Eriobotrya_japonica.EVM0040033.1
Malus_domestica.MD15G1215500_MdMYB12
Pyrus.546.GWHPAAYT021472
Eriobotrya_japonica.EVM0031605.1
Pyrus.546.GWHPAAYT031782
Malus_domestica.MD02G1087900
Prunus_persica.Prupe.7G201600.1.p
Fvesca.12738-v1.0-hybrid.t2
Rosa_chinensis.RcHm_v2.0_Chr2g0094491
Rosaceae Expansion
Eriobotrya_japonica.EVM0021312.1
Malus_domestica.MD09G1184000_MdMYB11
Eriobotrya_japonica.EVM0033490.1
Pyrus.546.GWHPAAYT029582
Prunus_persica.Prupe.3G042300.1.p
Rosa_chinensis.RcHm_v2.0_Chr2g0145191
Glycine_max.Glyma.16G007200.1.p
Glycine_max.Glyma.07G038000.1.p
Vigna_unguiculata.Vigun10g165300.1.p
Cecan.7G241000.1.p
Theobroma.636.EOY27233
Vitis_vinifera.VIT_209s0002g01400
Eriobotrya_japonica.EVM0037228.1
Malus_domestica.MD08G1070800
Pyrus.546.GWHPAAYT051244
Pyrus.546.GWHPAAYT019919
Fvesca.15391-v1.0-hybrid.t1
Rosa_chinensis.RcHm_v2.0_Chr6g0311701
Prunus_persica.Prupe.1G405300.1.p
Cecan.4G365400.1.p
Eriobotrya_japonica.EVM0037007.1
Malus_domestica.MD08G1070900
Prunus_persica.Prupe.1G405100.1.p
Fvesca.15386-v1.0-hybrid.t1
Rosa_chinensis.RcHm_v2.0_Chr6g0311741
Vitis_vinifera.VIT_204s0008g01830
Fvesca.08694-v1.0-hybrid.t1
Rosa_chinensis.RcHm_v2.0_Chr6g0304811
Vitis_vinifera.VIT_204s0008g01810
Theobroma.636.EOX94909
Theobroma.636.EOY00854
Theobroma.636.EOY30028
Malus_domestica.MD15G1051000
Pyrus.546.GWHPAAYT019918
Eriobotrya_japonica.EVM0039804.1
Prunus_persica.Prupe.1G405200.1.p
Fvesca.15390-v1.0-hybrid.t1
Rosa_chinensis.RcHm_v2.0_Chr6g0311711
Theobroma.636.EOY30030
Rosa_chinensis.RcHm_v2.0_Chr7g0229011
Vitis_vinifera.VIT_204s0008g01840
Vitis_vinifera.VIT_213s0084g00530
Brassica_rapa.Brara.H00653.1.p
Raphanus_sativus.Rs443870
AT5G35550.1_ATMYB123
Datura.112.GWHPBJWV029961
Solanum_lycopersicum.Solyc12g049300.2.1
Eriobotrya_japonica.EVM0037957.1
Pyrus.546.GWHPAAYT021468
Malus_domestica.MD15G1215400
Malus_domestica.MD02G1087700
Pyrus.546.GWHPAAYT031781
Eriobotrya_japonica.EVM0015177.1
Rosa_chinensis.RcHm_v2.0_Chr2g0094481
Prunus_persica.Prupe.7G201700.1.p
Prunus_persica.Prupe.7G201800.1.p
Vitis_vinifera.VIT_211s0016g01310
Vitis_vinifera.VIT_211s0016g01320_VvMYBPA2
Theobroma.636.EOY30300
Prunus_persica.Prupe.7G201900.1.p
Fvesca.12736-v2.0.a2-hybrid.t1
Rosa_chinensis.RcHm_v2.0_Chr2g0094451
Rosa_chinensis.RcHm_v2.0_Chr2g0094411
Prunus_persica.Prupe.4G204800.1.p
Pyrus.546.GWHPAAYT021464
Glycine_max.Glyma.20G184200.1.p
Glycine_max.Glyma.10G206400.1.p
Vigna_unguiculata.Vigun07g197100.1.p
Glycine_max.Glyma.20G184100.1.p
Glycine_max.Glyma.10G206500.1.p
Vigna_unguiculata.Vigun07g197200.1.p
Cecan.2G241700.1.p
Theobroma.636.EOX91295
Musa_acuminata.GSMUA_Achr3P07940_001
Musa_acuminata.GSMUA_Achr9P16040_001
Musa_acuminata.GSMUA_Achr8P22690_001
Ananas_comosus.Aco023262.1
Ananas_comosus.Aco023267.1
Dendrobium_catenatum.Dca020672817.1
Dendrobium_catenatum.Dca028549800.1
Dendrobium_catenatum.Dca020672813.1
Phalaenopsis_equestris.Peq020582444.1_PeMYB2
Phalaenopsis_equestris.Peq020578104.1
Phalaenopsis_equestris.Peq020573646.1
Phalaenopsis_equestris.Peq020573648.1_PeMYB11
Phalaenopsis_equestris.Peq020573647.1
Phalaenopsis_equestris.Peq020573611.1
Dendrobium_catenatum.Dca020692442.1
Zea_mays.Zm00001d037118_P001_ZmPL
Zea_mays.Zm00001d044975_P001_ZmC1
Oryza_sativa.LOC_Os06g10350.1_OsC1
Ananas_comosus.Aco013173.1
Ananas_comosus.Aco025712.1
Musa_acuminata.GSMUA_Achr3P27700_001
Dioscorea_alata.Dioal.01G097000.1.p
Allium.g45598.t1
Dendrobium_catenatum.Dca020682460.2
LregChr02G072530.mRNA1
Ananas_comosus.Aco023263.1
Ananas_comosus.Aco023266.1
Dioscorea_alata.Dioal.18G090200.1.p
Musa_acuminata.GSMUA_Achr3P07950_001
Ananas_comosus.Aco009478.1
Ananas_comosus.Aco030108.1
Musa_acuminata.GSMUA_Achr10P17610_001_MaMYBPA2
Brachypodium_distachyon.Bradi1g60106.1.p
Oryza_sativa.LOC_Os03g29614.1_OsMYB3
Musa_acuminata.GSMUA_Achr9P27380_001_MaMYBA2
Musa_acuminata.GSMUA_Achr6P04400_001_MaMYBA1
Gs05G140590.1
Musa_acuminata.GSMUA_Achr7P18550_001
Musa_acuminata.GSMUA_Achr6P02530_001
Musa_acuminata.GSMUA_Achr7P18560_001
Euryale_ferox.g25310.t1
Euryale_ferox.g25490.t1
Euryale_ferox.g23551.t1
Vc9G033540.1
Nymphaea_colorata.Nycol.H01159.1.p
Euryale_ferox.g05990.t1
Nymphaea_colorata.Nycol.J00062.1.p
Amborella_trichopoda.scaffold00024.222
Amborella_trichopoda.scaffold00024.223
Amborella_trichopoda.scaffold00024.221
Tree scale 0.2
Tree scale 0.1

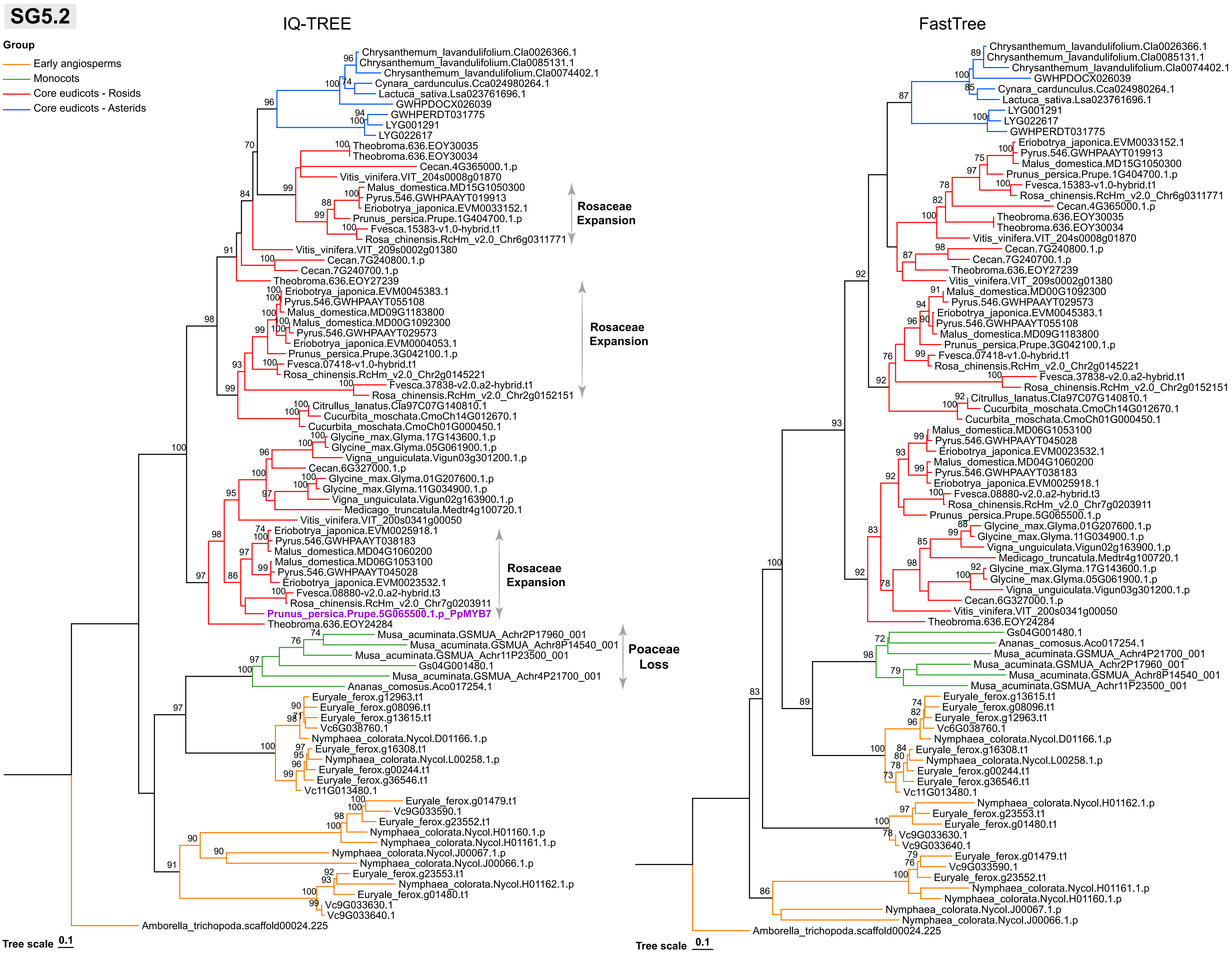
SG5.2
IQ-TREE
FastTree
Group
Early angiosperms
Monocots
Core eudicots - Rosids
Core eudicots - Asterids
Chrysanthemum_lavandulifolium.Cla0026366.1
Chrysanthemum_lavandulifolium.Cla0085131.1
Chrysanthemum_lavandulifolium.Cla0074402.1
Cynara_cardunculus.Cca024980264.1
Lactuca_sativa.Lsa023761696.1
GWHPDOCX026039
GWHPERDT031775
LYG001291
LYG022617
Theobroma.636.EOY30035
Theobroma.636.EOY30034
Cecan.4G365000.1.p
Vitis_vinifera.VIT_204s0008g01870
Malus_domestica.MD15G1050300
Pyrus.546.GWHPAAYT019913
Eriobotrya_japonica.EVM0033152.1
Prunus_persica.Prupe.1G404700.1.p
Fvesca.15383-v1.0-hybrid.t1
Rosa_chinensis.RcHm_v2.0_Chr6g0311771
Vitis_vinifera.VIT_209s0002g01380
Cecan.7G240800.1.p
Cecan.7G240700.1.p
Theobroma.636.EOY27239
Rosaceae Expansion
Eriobotrya_japonica.EVM0045383.1
Pyrus.546.GWHPAAYT055108
Malus_domestica.MD09G1183800
Malus_domestica.MD00G1092300
Pyrus.546.GWHPAAYT029573
Eriobotrya_japonica.EVM0004053.1
Prunus_persica.Prupe.3G042100.1.p
Fvesca.07418-v1.0-hybrid.t1
Rosa_chinensis.RcHm_v2.0_Chr2g0145221
Fvesca.37838-v2.0.a2-hybrid.t1
Rosa_chinensis.RcHm_v2.0_Chr2g0152151
Rosaceae Expansion
Citrullus_lanatus.Cla97C07G140810.1
Cucurbita_moschata.CmoCh14G012670.1
Cucurbita_moschata.CmoCh01G000450.1
Glycine_max.Glyma.17G143600.1.p
Glycine_max.Glyma.05G061900.1.p
Vigna_unguiculata.Vigun03g301200.1.p
Cecan.6G327000.1.p
Glycine_max.Glyma.01G207600.1.p
Glycine_max.Glyma.11G034900.1.p
Vigna_unguiculata.Vigun02g163900.1.p
Medicago_truncatula.Medtr4g100720.1
Vitis_vinifera.VIT_200s0341g00050
Eriobotrya_japonica.EVM0025918.1
Pyrus.546.GWHPAAYT038183
Malus_domestica.MD04G1060200
Malus_domestica.MD06G1053100
Pyrus.546.GWHPAAYT045028
Eriobotrya_japonica.EVM0023532.1
Fvesca.08880-v2.0.a2-hybrid.t3
Rosa_chinensis.RcHm_v2.0_Chr7g0203911
Prunus_persica.Prupe.5G065500.1.p_PpMYB7
Theobroma.636.EOY24284
Rosaceae Expansion
Musa_acuminata.GSMUA_Achr2P17960_001
Musa_acuminata.GSMUA_Achr8P14540_001
Musa_acuminata.GSMUA_Achr11P23500_001
Gs04G001480.1
Musa_acuminata.GSMUA_Achr4P21700_001
Ananas_comosus.Aco017254.1
Poaceae Loss
Euryale_ferox.g12963.t1
Euryale_ferox.g08096.t1
Euryale_ferox.g13615.t1
Vc6G038760.1
Nymphaea_colorata.Nycol.D01166.1.p
Euryale_ferox.g16308.t1
Nymphaea_colorata.Nycol.L00258.1.p
Euryale_ferox.g00244.t1
Euryale_ferox.g36546.t1
Vc11G013480.1
Euryale_ferox.g01479.t1
Vc9G033590.1
Euryale_ferox.g23552.t1
Nymphaea_colorata.Nycol.H01160.1.p
Nymphaea_colorata.Nycol.H01161.1.p
Nymphaea_colorata.Nycol.J00067.1.p
Nymphaea_colorata.Nycol.J00066.1.p
Euryale_ferox.g23553.t1
Nymphaea_colorata.Nycol.H01162.1.p
Euryale_ferox.g01480.t1
Vc9G033630.1
Vc9G033640.1
Amborella_trichopoda.scaffold00024.225
Tree scale 0.1
Tree scale 0.1

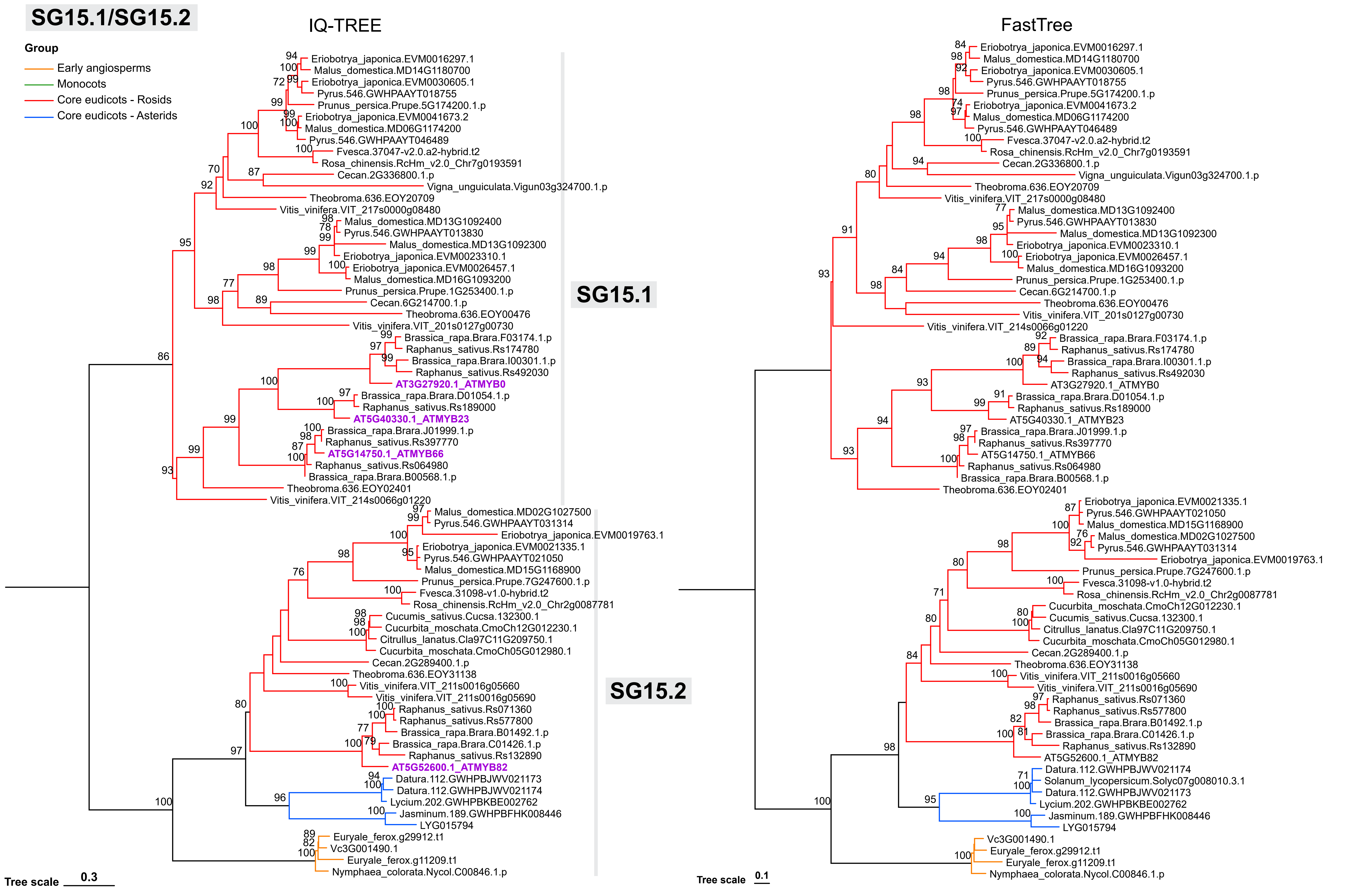

SG15.1/SG15.2
IQ-TREE
FastTree
Group
Early angiosperms
Monocots
Core eudicots - Rosids
Core eudicots - Asterids
SG15.1
SG15.2
Eriobotrya_japonica.EVM0016297.1
Malus_domestica.MD14G1180700
Eriobotrya_japonica.EVM0030605.1
Pyrus.546.GWHPAAYT018755
Prunus_persica.Prupe.5G174200.1.p
Eriobotrya_japonica.EVM0041673.2
Malus_domestica.MD06G1174200
Pyrus.546.GWHPAAYT046489
Fvesca.37047-v2.0.a2-hybrid.t2
Rosa_chinensis.RcHm_v2.0_Chr7g0193591
Cecan.2G336800.1.p
Vigna_unguiculata.Vigun03g324700.1.p
Theobroma.636.EOY20709
Vitis_vinifera.VIT_217s0000g08480
Malus_domestica.MD13G1092400
Pyrus.546.GWHPAAYT013830
Malus_domestica.MD13G1092300
Eriobotrya_japonica.EVM0023310.1
Eriobotrya_japonica.EVM0026457.1
Malus_domestica.MD16G1093200
Prunus_persica.Prupe.1G253400.1.p
Cecan.6G214700.1.p
Theobroma.636.EOY00476
Vitis_vinifera.VIT_201s0127g00730
Brassica_rapa.Brara.F03174.1.p
Raphanus_sativus.Rs174780
Brassica_rapa.Brara.I00301.1.p
Raphanus_sativus.Rs492030
AT3G27920.1_ATMYB0
Brassica_rapa.Brara.D01054.1.p
Raphanus_sativus.Rs189000
AT5G40330.1_ATMYB23
Brassica_rapa.Brara.J01999.1.p
Raphanus_sativus.Rs397770
AT5G14750.1_ATMYB66
Raphanus_sativus.Rs064980
Brassica_rapa.Brara.B00568.1.p
Theobroma.636.EOY02401
Vitis_vinifera.VIT_214s0066g01220
Malus_domestica.MD02G1027500
Pyrus.546.GWHPAAYT031314
Eriobotrya_japonica.EVM0019763.1
Eriobotrya_japonica.EVM0021335.1
Pyrus.546.GWHPAAYT021050
Malus_domestica.MD15G1168900
Prunus_persica.Prupe.7G247600.1.p
Fvesca.31098-v1.0-hybrid.t2
Rosa_chinensis.RcHm_v2.0_Chr2g0087781
Cucumis_sativus.Cucsa.132300.1
Cucurbita_moschata.CmoCh12G012230.1
Citrullus_lanatus.Cla97C11G209750.1
Cucurbita_moschata.CmoCh05G012980.1
Cecan.2G289400.1.p
Theobroma.636.EOY31138
Vitis_vinifera.VIT_211s0016g05660
Vitis_vinifera.VIT_211s0016g05690
Raphanus_sativus.Rs071360
Raphanus_sativus.Rs577800
Brassica_rapa.Brara.B01492.1.p
Brassica_rapa.Brara.C01426.1.p
Raphanus_sativus.Rs132890
AT5G52600.1_ATMYB82
Datura.112.GWHPBJWV021173
Datura.112.GWHPBJWV021174
Solanum_lycopersicum.Solyc07g008010.3.1
Lycium.202.GWHPBKBE002762
Jasminum.189.GWHPBFHK008446
LYG015794
Euryale_ferox.g29912.t1
Vc3G001490.1
Euryale_ferox.g11209.t1
Nymphaea_colorata.Nycol.C00846.1.p
Tree scale 0.3
Tree scale 0.1

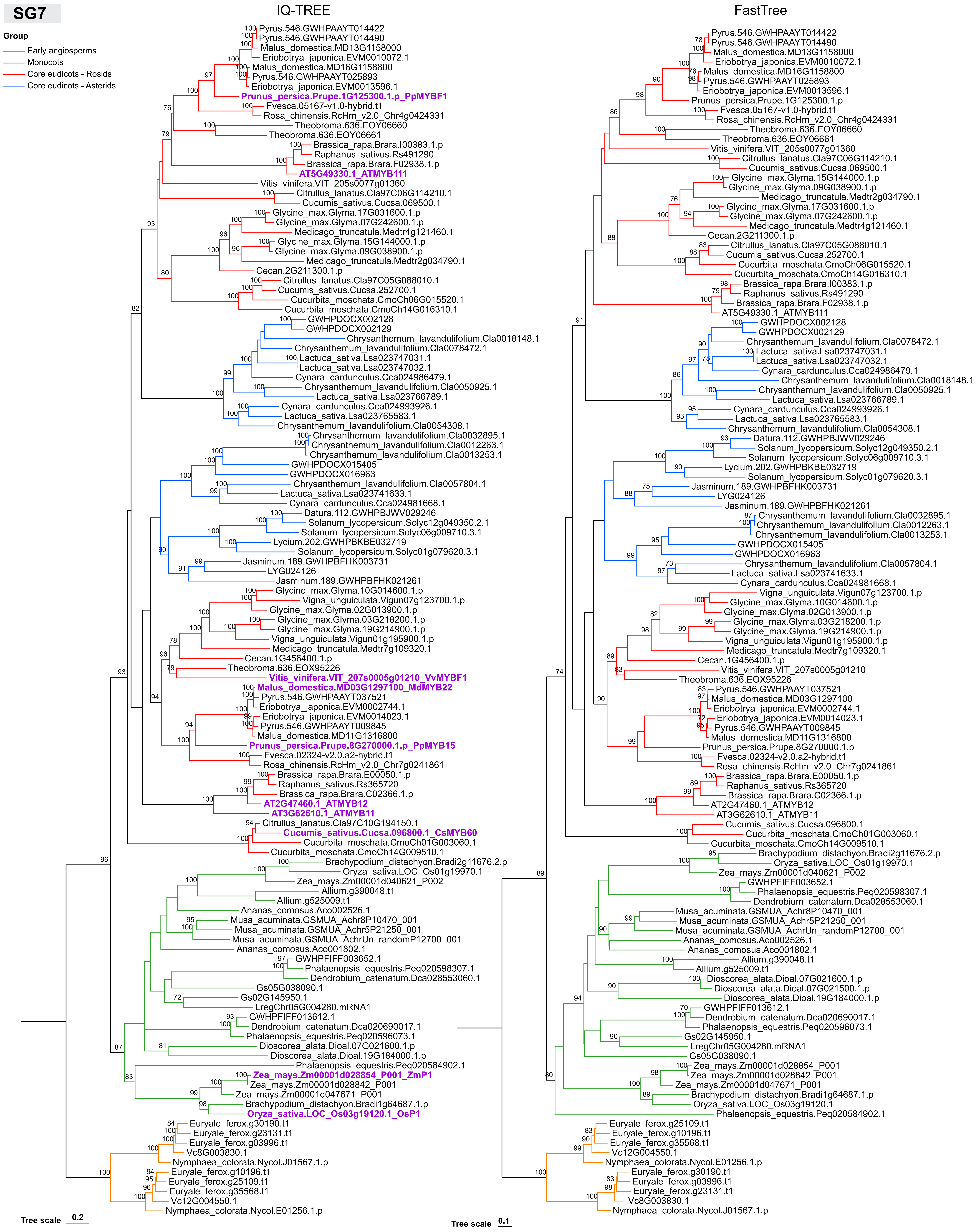

SG7
Group
Early angiosperms
Monocots
Core eudicots - Rosids
Core eudicots - Asterids
IQ-TREE
Pyrus.546.GWHPAAYT014422
Pyrus.546.GWHPAAYT014490
Malus_domestica.MD13G1158000
Eriobotrya_japonica.EVM0010072.1
Malus_domestica.MD16G1158800
Pyrus.546.GWHPAAYT025893
Eriobotrya_japonica.EVM0013596.1
Prunus_persica.Prupe.1G125300.1.p_PpMYBF1
Fvesca.05167-v1.0-hybrid.t1
Rosa_chinensis.RcHm_v2.0_Chr4g0424331
Theobroma.636.EOY06660
Theobroma.636.EOY06661
Brassica_rapa.Brara.I00383.1.p
Raphanus_sativus.Rs491290
Brassica_rapa.Brara.F02938.1.p
AT5G49330.1_ATMYB111
Vitis_vinifera.VIT_205s0077g01360
Citrullus_lanatus.Cla97C06G114210.1
Cucumis_sativus.Cucsa.069500.1
Glycine_max.Glyma.17G031600.1.p
Glycine_max.Glyma.07G242600.1.p
Medicago_truncatula.Medtr4g121460.1
Glycine_max.Glyma.15G144000.1.p
Glycine_max.Glyma.09G038900.1.p
Medicago_truncatula.Medtr2g034790.1
Cecan.2G211300.1.p
Citrullus_lanatus.Cla97C05G088010.1
Cucumis_sativus.Cucsa.252700.1
Cucurbita_moschata.CmoCh06G015520.1
Cucurbita_moschata.CmoCh14G016310.1
GWHPDOCX002128
GWHPDOCX002129
Chrysanthemum_lavandulifolium.Cla0018148.1
Chrysanthemum_lavandulifolium.Cla0078472.1
Lactuca_sativa.Lsa023747031.1
Lactuca_sativa.Lsa023747032.1
Cynara_cardunculus.Cca024986479.1
Chrysanthemum_lavandulifolium.Cla0050925.1
Lactuca_sativa.Lsa023766789.1
Cynara_cardunculus.Cca024993926.1
Lactuca_sativa.Lsa023765583.1
Chrysanthemum_lavandulifolium.Cla0054308.1
Chrysanthemum_lavandulifolium.Cla0032895.1
Chrysanthemum_lavandulifolium.Cla0012263.1
Chrysanthemum_lavandulifolium.Cla0013253.1
GWHPDOCX015405
GWHPDOCX016963
Chrysanthemum_lavandulifolium.Cla0057804.1
Lactuca_sativa.Lsa023741633.1
Cynara_cardunculus.Cca024981668.1
Datura.112.GWHPBJWV029246
Solanum_lycopersicum.Solyc12g049350.2.1
Solanum_lycopersicum.Solyc06g009710.3.1
Lycium.202.GWHPBKBE032719
Solanum_lycopersicum.Solyc01g079620.3.1
Jasminum.189.GWHPBFHK003731
LYG024126
Jasminum.189.GWHPBFHK021261
Glycine_max.Glyma.10G014600.1.p
Vigna_unguiculata.Vigun07g123700.1.p
Glycine_max.Glyma.02G013900.1.p
Glycine_max.Glyma.03G218200.1.p
Glycine_max.Glyma.19G214900.1.p
Vigna_unguiculata.Vigun01g195900.1.p
Medicago_truncatula.Medtr7g109320.1
Cecan.1G456400.1.p
Theobroma.636.EOX95226
Vitis_vinifera.VIT_207s0005g01210_VvMYBF1
Malus_domestica.MD03G1297100_MdMYB22
Pyrus.546.GWHPAAYT037521
Eriobotrya_japonica.EVM0002744.1
Eriobotrya_japonica.EVM0014023.1
Pyrus.546.GWHPAAYT009845
Malus_domestica.MD11G1316800
Prunus_persica.Prupe.8G270000.1.p_PpMYB15
Fvesca.02324-v2.0.a2-hybrid.t1
Rosa_chinensis.RcHm_v2.0_Chr7g0241861
Brassica_rapa.Brara.E00050.1.p
Raphanus_sativus.Rs365720
Brassica_rapa.Brara.C02366.1.p
AT2G47460.1_ATMYB12
AT3G62610.1_ATMYB11
Citrullus_lanatus.Cla97C10G194150.1
Cucumis_sativus.Cucsa.096800.1_CsMYB60
Cucurbita_moschata.CmoCh01G003060.1
Cucurbita_moschata.CmoCh14G009510.1
Brachypodium_distachyon.Bradi2g11676.2.p
Oryza_sativa.LOC_Os01g19970.1
Zea_mays.Zm00001d040621_P002
Allium.g390048.t1
Allium.g525009.t1
Ananas_comosus.Aco002526.1
Musa_acuminata.GSMUA_Achr8P10470_001
Musa_acuminata.GSMUA_Achr5P21250_001
Musa_acuminata.GSMUA_AchrUn_randomP12700_001
Ananas_comosus.Aco001802.1
GWHPFIFF003652.1
Phalaenopsis_equestris.Peq020598307.1
Dendrobium_catenatum.Dca028553060.1
Gs05G038090.1
Gs02G145950.1
LregChr05G004280.mRNA1
GWHPFIFF013612.1
Dendrobium_catenatum.Dca020690017.1
Phalaenopsis_equestris.Peq020596073.1
Dioscorea_alata.Dioal.07G021600.1.p
Dioscorea_alata.Dioal.19G184000.1.p
Phalaenopsis_equestris.Peq020584902.1
Zea_mays.Zm00001d028854_P001_ZmP1
Zea_mays.Zm00001d028842_P001
Zea_mays.Zm00001d047671_P001
Brachypodium_distachyon.Bradi1g64687.1.p
Oryza_sativa.LOC_Os03g19120.1_OsP1
Euryale_ferox.g30190.t1
Euryale_ferox.g23131.t1
Euryale_ferox.g03996.t1
Vc8G003830.1
Nymphaea_colorata.Nycol.J01567.1.p
Euryale_ferox.g10196.t1
Euryale_ferox.g25109.t1
Euryale_ferox.g35568.t1
Vc12G004550.1
Nymphaea_colorata.Nycol.E01256.1.p
Tree scale 0.2
FastTree
Pyrus.546.GWHPAAYT014422
Pyrus.546.GWHPAAYT014490
Malus_domestica.MD13G1158000
Eriobotrya_japonica.EVM0010072.1
Malus_domestica.MD16G1158800
Pyrus.546.GWHPAAYT025893
Eriobotrya_japonica.EVM0013596.1
Prunus_persica.Prupe.1G125300.1.p
Fvesca.05167-v1.0-hybrid.t1
Rosa_chinensis.RcHm_v2.0_Chr4g0424331
Theobroma.636.EOY06660
Theobroma.636.EOY06661
Vitis_vinifera.VIT_205s0077g01360
Citrullus_lanatus.Cla97C06G114210.1
Cucumis_sativus.Cucsa.069500.1
Glycine_max.Glyma.15G144000.1.p
Glycine_max.Glyma.09G038900.1.p
Medicago_truncatula.Medtr2g034790.1
Glycine_max.Glyma.17G031600.1.p
Glycine_max.Glyma.07G242600.1.p
Medicago_truncatula.Medtr4g121460.1
Cecan.2G211300.1.p
Citrullus_lanatus.Cla97C05G088010.1
Cucumis_sativus.Cucsa.252700.1
Cucurbita_moschata.CmoCh06G015520.1
Cucurbita_moschata.CmoCh14G016310.1
Brassica_rapa.Brara.I00383.1.p
Raphanus_sativus.Rs491290
Brassica_rapa.Brara.F02938.1.p
AT5G49330.1_ATMYB111
GWHPDOCX002128
GWHPDOCX002129
Chrysanthemum_lavandulifolium.Cla0078472.1
Lactuca_sativa.Lsa023747031.1
Lactuca_sativa.Lsa023747032.1
Cynara_cardunculus.Cca024986479.1
Chrysanthemum_lavandulifolium.Cla0018148.1
Chrysanthemum_lavandulifolium.Cla0050925.1
Lactuca_sativa.Lsa023766789.1
Cynara_cardunculus.Cca024993926.1
Lactuca_sativa.Lsa023765583.1
Chrysanthemum_lavandulifolium.Cla0054308.1
Datura.112.GWHPBJWV029246
Solanum_lycopersicum.Solyc12g049350.2.1
Solanum_lycopersicum.Solyc06g009710.3.1
Lycium.202.GWHPBKBE032719
Solanum_lycopersicum.Solyc01g079620.3.1
Jasminum.189.GWHPBFHK003731
LYG024126
Jasminum.189.GWHPBFHK021261
Chrysanthemum_lavandulifolium.Cla0032895.1
Chrysanthemum_lavandulifolium.Cla0012263.1
Chrysanthemum_lavandulifolium.Cla0013253.1
GWHPDOCX015405
GWHPDOCX016963
Chrysanthemum_lavandulifolium.Cla0057804.1
Lactuca_sativa.Lsa023741633.1
Cynara_cardunculus.Cca024981668.1
Vigna_unguiculata.Vigun07g123700.1.p
Glycine_max.Glyma.10G014600.1.p
Glycine_max.Glyma.02G013900.1.p
Glycine_max.Glyma.03G218200.1.p
Glycine_max.Glyma.19G214900.1.p
Vigna_unguiculata.Vigun01g195900.1.p
Medicago_truncatula.Medtr7g109320.1
Cecan.1G456400.1.p
Vitis_vinifera.VIT_207s0005g01210
Theobroma.636.EOX95226
Pyrus.546.GWHPAAYT037521
Malus_domestica.MD03G1297100
Eriobotrya_japonica.EVM0002744.1
Eriobotrya_japonica.EVM0014023.1
Pyrus.546.GWHPAAYT009845
Malus_domestica.MD11G1316800
Prunus_persica.Prupe.8G270000.1.p
Fvesca.02324-v2.0.a2-hybrid.t1
Rosa_chinensis.RcHm_v2.0_Chr7g0241861
Brassica_rapa.Brara.E00050.1.p
Raphanus_sativus.Rs365720
Brassica_rapa.Brara.C02366.1.p
AT2G47460.1_ATMYB12
AT3G62610.1_ATMYB11
Cucumis_sativus.Cucsa.096800.1
Cucurbita_moschata.CmoCh01G003060.1
Cucurbita_moschata.CmoCh14G009510.1
Brachypodium_distachyon.Bradi2g11676.2.p
Oryza_sativa.LOC_Os01g19970.1
Zea_mays.Zm00001d040621_P002
GWHPFIFF003652.1
Phalaenopsis_equestris.Peq020598307.1
Dendrobium_catenatum.Dca028553060.1
Musa_acuminata.GSMUA_Achr8P10470_001
Musa_acuminata.GSMUA_Achr5P21250_001
Musa_acuminata.GSMUA_AchrUn_randomP12700_001
Ananas_comosus.Aco002526.1
Ananas_comosus.Aco001802.1
Allium.g390048.t1
Allium.g525009.t1
Dioscorea_alata.Dioal.07G021600.1.p
Dioscorea_alata.Dioal.07G021500.1.p
Dioscorea_alata.Dioal.19G184000.1.p
GWHPFIFF013612.1
Dendrobium_catenatum.Dca020690017.1
Phalaenopsis_equestris.Peq020596073.1
Gs02G145950.1
LregChr05G004280.mRNA1
Gs05G038090.1
Zea_mays.Zm00001d028854_P001
Zea_mays.Zm00001d028842_P001
Zea_mays.Zm00001d047671_P001
Brachypodium_distachyon.Bradi1g64687.1.p
Oryza_sativa.LOC_Os03g19120.1
Phalaenopsis_equestris.Peq020584902.1
Euryale_ferox.g25109.t1
Euryale_ferox.g10196.t1
Euryale_ferox.g35568.t1
Vc12G004550.1
Nymphaea_colorata.Nycol.E01256.1.p
Euryale_ferox.g30190.t1
Euryale_ferox.g03996.t1
Euryale_ferox.g23131.t1
Vc8G003830.1
Nymphaea_colorata.Nycol.J01567.1.p
Tree scale 0.1

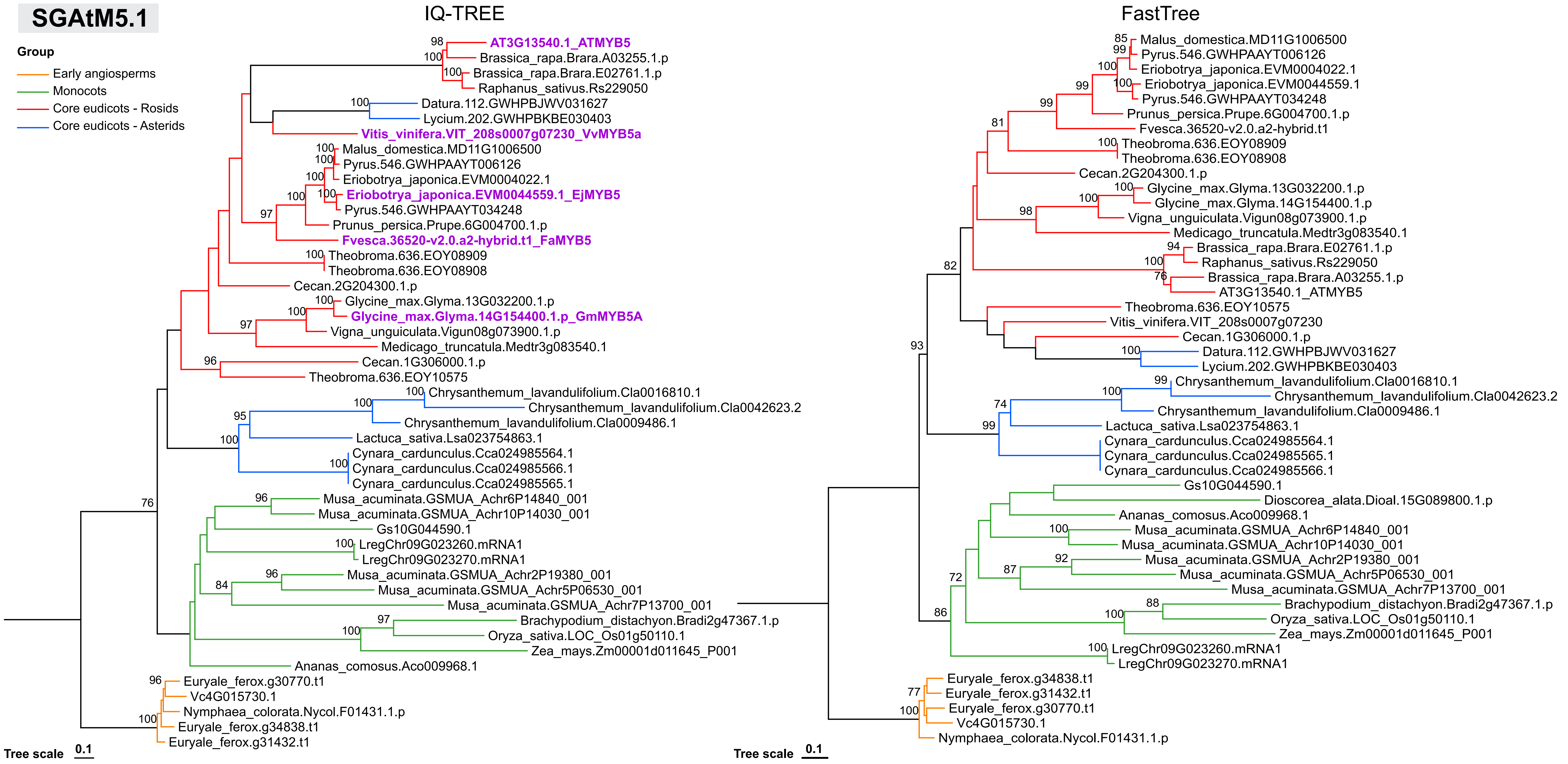
SGAtM5.1
Group
Early angiosperms
Monocots
Core eudicots - Rosids
Core eudicots - Asterids
IQ-TREE
98
100
AT3G13540.1_ATMYB5
Brassica_rapa.Brara.A03255.1.p
100
Brassica_rapa.Brara.E02761.1.p
Raphanus_sativus.Rs229050
100
Datura.112.GWHPBJWV031627
Lycium.202.GWHPBKBE030403
Vitis_vinifera.VIT_208s0007g07230_VvMYB5a
100
Malus_domestica.MD11G1006500
100
Pyrus.546.GWHPAAYT006126
100
Eriobotrya_japonica.EVM0004022.1
100
100
Eriobotrya_japonica.EVM0044559.1_EjMYB5
Pyrus.546.GWHPAAYT034248
97
Prunus_persica.Prupe.6G004700.1.p
Fvesca.36520-v2.0.a2-hybrid.t1_FaMYB5
100
Theobroma.636.EOY08909
Theobroma.636.EOY08908
Cecan.2G204300.1.p
100
Glycine_max.Glyma.13G032200.1.p
100
Glycine_max.Glyma.14G154400.1.p_GmMYB5A
97
Vigna_unguiculata.Vigun08g073900.1.p
Medicago_truncatula.Medtr3g083540.1
96
Cecan.1G306000.1.p
Theobroma.636.EOY10575
100
Chrysanthemum_lavandulifolium.Cla0016810.1
100
Chrysanthemum_lavandulifolium.Cla0042623.2
95
Chrysanthemum_lavandulifolium.Cla0009486.1
100
Lactuca_sativa.Lsa023754863.1
Cynara_cardunculus.Cca024985564.1
100
Cynara_cardunculus.Cca024985566.1
Cynara_cardunculus.Cca024985565.1
96
Musa_acuminata.GSMUA_Achr6P14840_001
Musa_acuminata.GSMUA_Achr10P14030_001
76
Gs10G044590.1
100
LregChr09G023260.mRNA1
LregChr09G023270.mRNA1
96
Musa_acuminata.GSMUA_Achr2P19380_001
84
Musa_acuminata.GSMUA_Achr5P06530_001
Musa_acuminata.GSMUA_Achr7P13700_001
97
Brachypodium_distachyon.Bradi2g47367.1.p
100
Oryza_sativa.LOC_Os01g50110.1
Zea_mays.Zm00001d011645_P001
Ananas_comosus.Aco009968.1
96
Euryale_ferox.g30770.t1
Vc4G015730.1
100
Nymphaea_colorata.Nycol.F01431.1.p
Euryale_ferox.g34838.t1
Euryale_ferox.g31432.t1
Tree scale 0.1
FastTree
85
Malus_domestica.MD11G1006500
99
Pyrus.546.GWHPAAYT006126
100
Eriobotrya_japonica.EVM0004022.1
99
100
Eriobotrya_japonica.EVM0044559.1
99
Pyrus.546.GWHPAAYT034248
Prunus_persica.Prupe.6G004700.1.p
81
Fvesca.36520-v2.0.a2-hybrid.t1
100
Theobroma.636.EOY08909
Theobroma.636.EOY08908
Cecan.2G204300.1.p
100
Glycine_max.Glyma.13G032200.1.p
100
Glycine_max.Glyma.14G154400.1.p
98
Vigna_unguiculata.Vigun08g073900.1.p
Medicago_truncatula.Medtr3g083540.1
94
Brassica_rapa.Brara.E02761.1.p
100
Raphanus_sativus.Rs229050
76
Brassica_rapa.Brara.A03255.1.p
82
AT3G13540.1_ATMYB5
Theobroma.636.EOY10575
Vitis_vinifera.VIT_208s0007g07230
Cecan.1G306000.1.p
100
Datura.112.GWHPBJWV031627
93
Lycium.202.GWHPBKBE030403
99
Chrysanthemum_lavandulifolium.Cla0016810.1
100
Chrysanthemum_lavandulifolium.Cla0042623.2
74
Chrysanthemum_lavandulifolium.Cla0009486.1
99
Lactuca_sativa.Lsa023754863.1
Cynara_cardunculus.Cca024985564.1
Cynara_cardunculus.Cca024985565.1
Cynara_cardunculus.Cca024985566.1
Gs10G044590.1
Dioscorea_alata.Dioal.15G089800.1.p
Ananas_comosus.Aco009968.1
100
Musa_acuminata.GSMUA_Achr6P14840_001
Musa_acuminata.GSMUA_Achr10P14030_001
92
Musa_acuminata.GSMUA_Achr2P19380_001
87
Musa_acuminata.GSMUA_Achr5P06530_001
72
Musa_acuminata.GSMUA_Achr7P13700_001
88
Brachypodium_distachyon.Bradi2g47367.1.p
86
100
Oryza_sativa.LOC_Os01g50110.1
Zea_mays.Zm00001d011645_P001
100
LregChr09G023260.mRNA1
LregChr09G023270.mRNA1
Euryale_ferox.g34838.t1
77
Euryale_ferox.g31432.t1
Euryale_ferox.g30770.t1
100
Vc4G015730.1
Nymphaea_colorata.Nycol.F01431.1.p
Tree scale 0.1

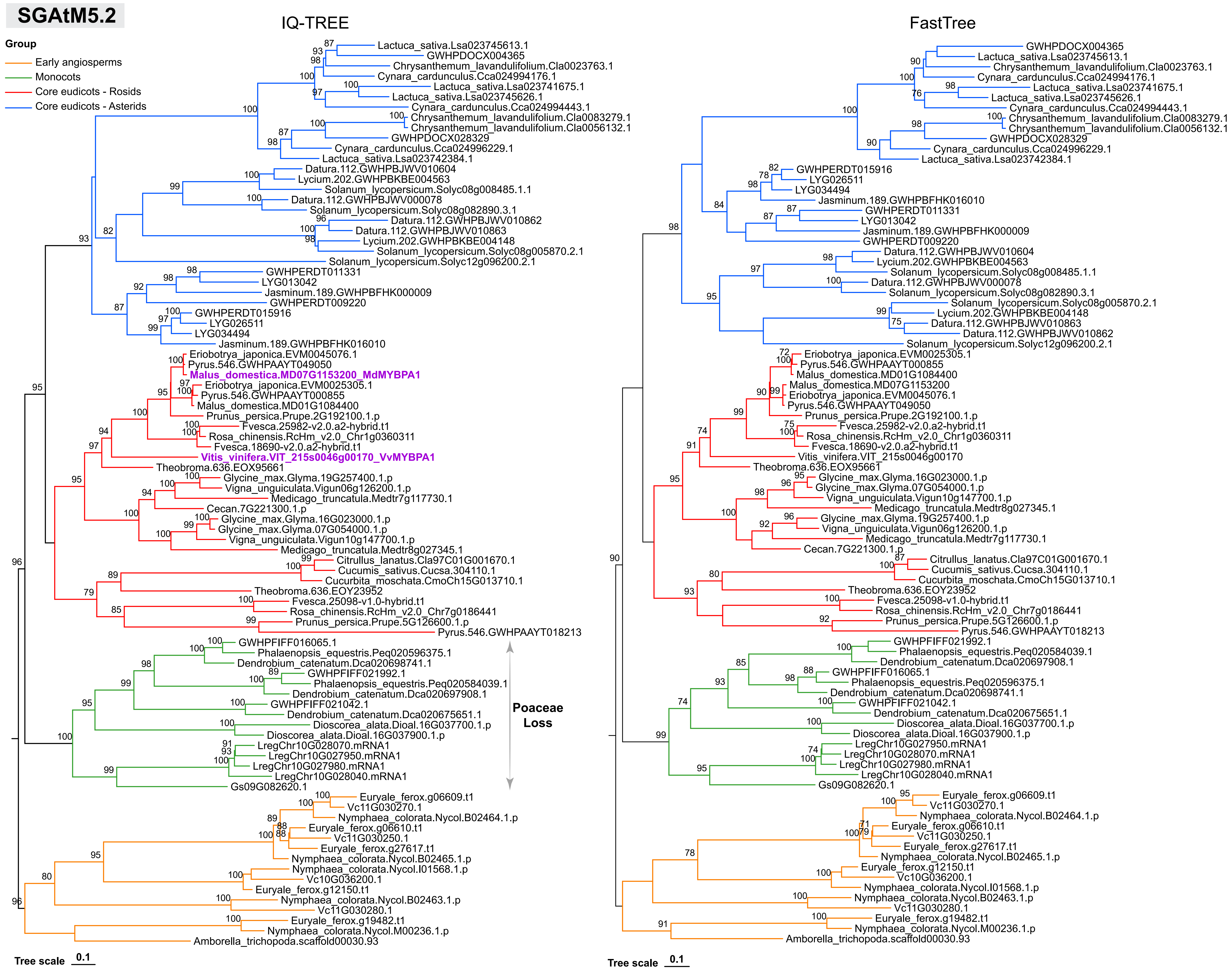
SGAtM5.2
IQ-TREE
FastTree
Group
Early angiosperms
Monocots
Core eudicots - Rosids
Core eudicots - Asterids
Lactuca_sativa.Lsa023745613.1
GWHPDOCX004365
Chrysanthemum_lavandulifolium.Cla0023763.1
Cynara_cardunculus.Cca024994176.1
Lactuca_sativa.Lsa023741675.1
Lactuca_sativa.Lsa023745626.1
Cynara_cardunculus.Cca024994443.1
Chrysanthemum_lavandulifolium.Cla0083279.1
Chrysanthemum_lavandulifolium.Cla0056132.1
GWHPDOCX028329
Cynara_cardunculus.Cca024996229.1
Lactuca_sativa.Lsa023742384.1
Datura.112.GWHPBJWV010604
Lycium.202.GWHPBKBE004563
Solanum_lycopersicum.Solyc08g008485.1.1
Datura.112.GWHPBJWV000078
Solanum_lycopersicum.Solyc08g082890.3.1
Datura.112.GWHPBJWV010862
Datura.112.GWHPBJWV010863
Lycium.202.GWHPBKBE004148
Solanum_lycopersicum.Solyc08g005870.2.1
Solanum_lycopersicum.Solyc12g096200.2.1
GWHPERDT011331
LYG013042
Jasminum.189.GWHPBFHK000009
GWHPERDT009220
GWHPERDT015916
LYG026511
LYG034494
Jasminum.189.GWHPBFHK016010
Eriobotrya_japonica.EVM0045076.1
Pyrus.546.GWHPAAYT049050
Malus_domestica.MD07G1153200_MdMYBPA1
Eriobotrya_japonica.EVM0025305.1
Pyrus.546.GWHPAAYT000855
Malus_domestica.MD01G1084400
Prunus_persica.Prupe.2G192100.1.p
Fvesca.25982-v2.0.a2-hybrid.t1
Rosa_chinensis.RcHm_v2.0_Chr1g0360311
Fvesca.18690-v2.0.a2-hybrid.t1
Vitis_vinifera.VIT_215s0046g00170_VvMYBPA1
Theobroma.636.EOX95661
Glycine_max.Glyma.19G257400.1.p
Vigna_unguiculata.Vigun06g126200.1.p
Medicago_truncatula.Medtr7g117730.1
Cecan.7G221300.1.p
Glycine_max.Glyma.16G023000.1.p
Glycine_max.Glyma.07G054000.1.p
Vigna_unguiculata.Vigun10g147700.1.p
Medicago_truncatula.Medtr8g027345.1
Citrullus_lanatus.Cla97C01G001670.1
Cucumis_sativus.Cucsa.304110.1
Cucurbita_moschata.CmoCh15G013710.1
Theobroma.636.EOY23952
Fvesca.25098-v1.0-hybrid.t1
Rosa_chinensis.RcHm_v2.0_Chr7g0186441
Prunus_persica.Prupe.5G126600.1.p
Pyrus.546.GWHPAAYT018213
GWHPFIFF016065.1
Phalaenopsis_equestris.Peq020596375.1
Dendrobium_catenatum.Dca020698741.1
GWHPFIFF021992.1
Phalaenopsis_equestris.Peq020584039.1
Dendrobium_catenatum.Dca020697908.1
GWHPFIFF021042.1
Dendrobium_catenatum.Dca020675651.1
Dioscorea_alata.Dioal.16G037700.1.p
Dioscorea_alata.Dioal.16G037900.1.p
LregChr10G028070.mRNA1
LregChr10G027950.mRNA1
LregChr10G027980.mRNA1
LregChr10G028040.mRNA1
Gs09G082620.1
Poaceae Loss
Euryale_ferox.g06609.t1
Vc11G030270.1
Nymphaea_colorata.Nycol.B02464.1.p
Euryale_ferox.g06610.t1
Vc11G030250.1
Euryale_ferox.g27617.t1
Nymphaea_colorata.Nycol.B02465.1.p
Nymphaea_colorata.Nycol.I01568.1.p
Vc10G036200.1
Euryale_ferox.g12150.t1
Nymphaea_colorata.Nycol.B02463.1.p
Vc11G030280.1
Euryale_ferox.g19482.t1
Nymphaea_colorata.Nycol.M00236.1.p
Amborella_trichopoda.scaffold00030.93
Tree scale 0.1
Malus_domestica.MD07G1153200
Vitis_vinifera.VIT_215s0046g00170
Tree scale 0.1

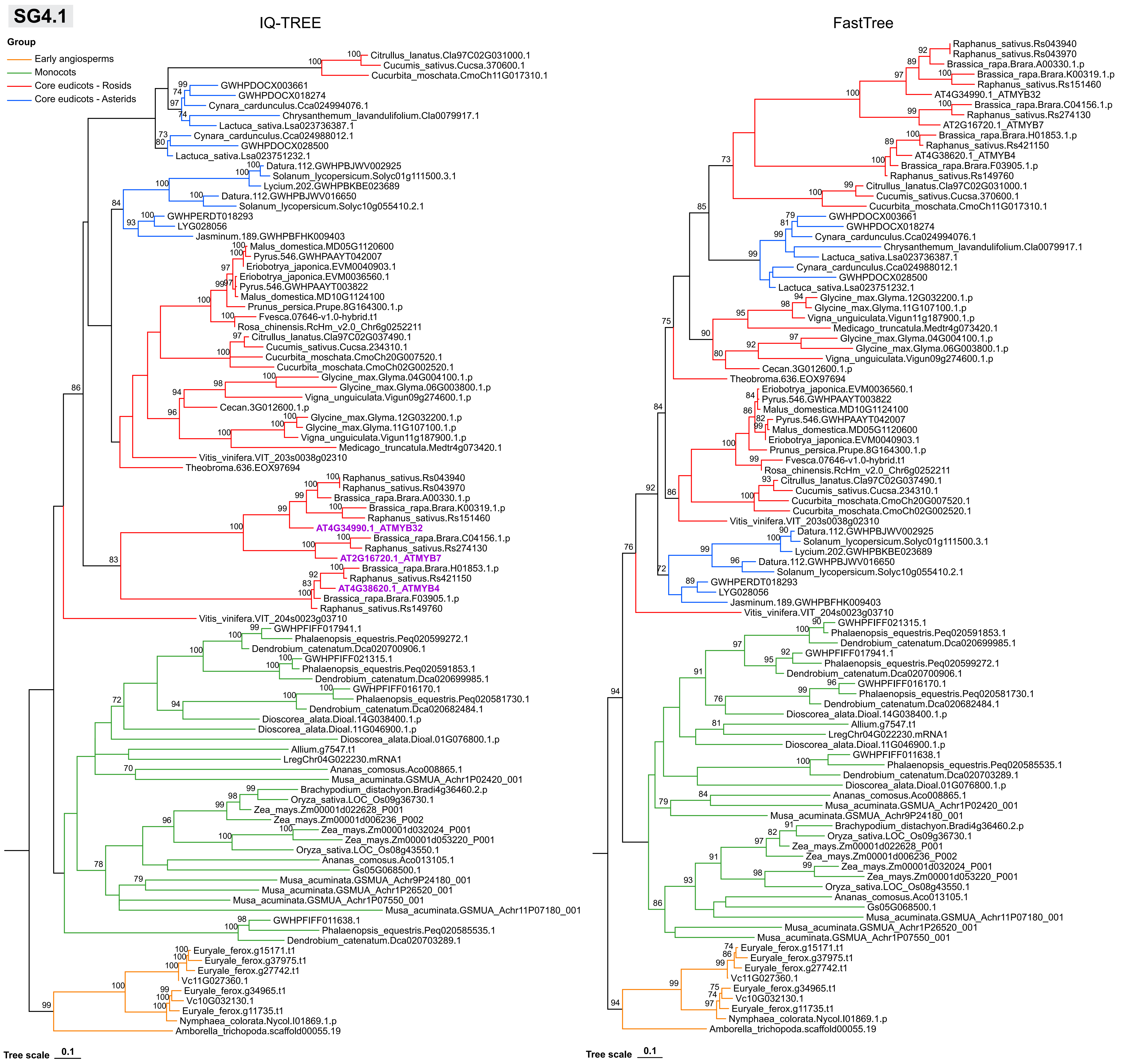
SG4.1
Group
Early angiosperms
Monocots
Core eudicots - Rosids
Core eudicots - Asterids
IQ-TREE
Citrullus_lanatus.Cla97C02G031000.1
Cucumis_sativus.Cucsa.370600.1
Cucurbita_moschata.CmoCh11G017310.1
GWHPDOCX003661
GWHPDOCX018274
Cynara_cardunculus.Cca024994076.1
Chrysanthemum_lavandulifolium.Cla0079917.1
Lactuca_sativa.Lsa023736387.1
Cynara_cardunculus.Cca024988012.1
GWHPDOCX028500
Lactuca_sativa.Lsa023751232.1
Datura.112.GWHPBJWV002925
Solanum_lycopersicum.Solyc01g111500.3.1
Lycium.202.GWHPBKBE023689
Datura.112.GWHPBJWV016650
Solanum_lycopersicum.Solyc10g055410.2.1
GWHPERDT018293
LYG028056
Jasminum.189.GWHPBFHK009403
Malus_domestica.MD05G1120600
Pyrus.546.GWHPAAYT042007
Eriobotrya_japonica.EVM0040903.1
Eriobotrya_japonica.EVM0036560.1
Pyrus.546.GWHPAAYT003822
Malus_domestica.MD10G1124100
Prunus_persica.Prupe.8G164300.1.p
Fvesca.07646-v1.0-hybrid.t1
Rosa_chinensis.RcHm_v2.0_Chr6g0252211
Citrullus_lanatus.Cla97C02G037490.1
Cucumis_sativus.Cucsa.234310.1
Cucurbita_moschata.CmoCh20G007520.1
Cucurbita_moschata.CmoCh02G002520.1
Glycine_max.Glyma.04G004100.1.p
Glycine_max.Glyma.06G003800.1.p
Vigna_unguiculata.Vigun09g274600.1.p
Cecan.3G012600.1.p
Glycine_max.Glyma.12G032200.1.p
Glycine_max.Glyma.11G107100.1.p
Vigna_unguiculata.Vigun11g187900.1.p
Medicago_truncatula.Medtr4g073420.1
Vitis_vinifera.VIT_203s0038g02310
Theobroma.636.EOX97694
Raphanus_sativus.Rs043940
Raphanus_sativus.Rs043970
Brassica_rapa.Brara.A00330.1.p
Brassica_rapa.Brara.K00319.1.p
Raphanus_sativus.Rs151460
AT4G34990.1_ATMYB32
Brassica_rapa.Brara.C04156.1.p
Raphanus_sativus.Rs274130
AT2G16720.1_ATMYB7
Brassica_rapa.Brara.H01853.1.p
Raphanus_sativus.Rs421150
AT4G38620.1_ATMYB4
Brassica_rapa.Brara.F03905.1.p
Raphanus_sativus.Rs149760
Vitis_vinifera.VIT_204s0023g03710
GWHPFIFF017941.1
Phalaenopsis_equestris.Peq020599272.1
Dendrobium_catenatum.Dca020700906.1
GWHPFIFF021315.1
Phalaenopsis_equestris.Peq020591853.1
Dendrobium_catenatum.Dca020699985.1
GWHPFIFF016170.1
Phalaenopsis_equestris.Peq020581730.1
Dendrobium_catenatum.Dca020682484.1
Dioscorea_alata.Dioal.14G038400.1.p
Dioscorea_alata.Dioal.11G046900.1.p
Dioscorea_alata.Dioal.01G076800.1.p
Allium.g7547.t1
LregChr04G022230.mRNA1
Ananas_comosus.Aco008865.1
Musa_acuminata.GSMUA_Achr1P02420_001
Brachypodium_distachyon.Bradi4g36460.2.p
Oryza_sativa.LOC_Os09g36730.1
Zea_mays.Zm00001d022628_P001
Zea_mays.Zm00001d006236_P002
Zea_mays.Zm00001d032024_P001
Zea_mays.Zm00001d053220_P001
Oryza_sativa.LOC_Os08g43550.1
Ananas_comosus.Aco013105.1
Gs05G068500.1
Musa_acuminata.GSMUA_Achr9P24180_001
Musa_acuminata.GSMUA_Achr1P26520_001
Musa_acuminata.GSMUA_Achr1P07550_001
Musa_acuminata.GSMUA_Achr11P07180_001
GWHPFIFF011638.1
Phalaenopsis_equestris.Peq020585535.1
Dendrobium_catenatum.Dca020703289.1
Euryale_ferox.g15171.t1
Euryale_ferox.g37975.t1
Euryale_ferox.g27742.t1
Vc11G027360.1
Euryale_ferox.g34965.t1
Vc10G032130.1
Euryale_ferox.g11735.t1
Nymphaea_colorata.Nycol.I01869.1.p
Amborella_trichopoda.scaffold00055.19
Tree scale 0.1
FastTree
Raphanus_sativus.Rs043940
Raphanus_sativus.Rs043970
Brassica_rapa.Brara.A00330.1.p
Brassica_rapa.Brara.K00319.1.p
Raphanus_sativus.Rs151460
AT4G34990.1_ATMYB32
Brassica_rapa.Brara.C04156.1.p
Raphanus_sativus.Rs274130
AT2G16720.1_ATMYB7
Brassica_rapa.Brara.H01853.1.p
Raphanus_sativus.Rs421150
AT4G38620.1_ATMYB4
Brassica_rapa.Brara.F03905.1.p
Raphanus_sativus.Rs149760
Citrullus_lanatus.Cla97C02G031000.1
Cucumis_sativus.Cucsa.370600.1
Cucurbita_moschata.CmoCh11G017310.1
GWHPDOCX003661
GWHPDOCX018274
Cynara_cardunculus.Cca024994076.1
Chrysanthemum_lavandulifolium.Cla0079917.1
Lactuca_sativa.Lsa023736387.1
Cynara_cardunculus.Cca024988012.1
GWHPDOCX028500
Lactuca_sativa.Lsa023751232.1
Glycine_max.Glyma.12G032200.1.p
Glycine_max.Glyma.11G107100.1.p
Vigna_unguiculata.Vigun11g187900.1.p
Medicago_truncatula.Medtr4g073420.1
Glycine_max.Glyma.04G004100.1.p
Glycine_max.Glyma.06G003800.1.p
Vigna_unguiculata.Vigun09g274600.1.p
Cecan.3G012600.1.p
Theobroma.636.EOX97694
Eriobotrya_japonica.EVM0036560.1
Pyrus.546.GWHPAAYT003822
Malus_domestica.MD10G1124100
Pyrus.546.GWHPAAYT042007
Malus_domestica.MD05G1120600
Eriobotrya_japonica.EVM0040903.1
Prunus_persica.Prupe.8G164300.1.p
Fvesca.07646-v1.0-hybrid.t1
Rosa_chinensis.RcHm_v2.0_Chr6g0252211
Citrullus_lanatus.Cla97C02G037490.1
Cucumis_sativus.Cucsa.234310.1
Cucurbita_moschata.CmoCh20G007520.1
Cucurbita_moschata.CmoCh02G002520.1
Vitis_vinifera.VIT_203s0038g02310
Datura.112.GWHPBJWV002925
Solanum_lycopersicum.Solyc01g111500.3.1
Lycium.202.GWHPBKBE023689
Datura.112.GWHPBJWV016650
Solanum_lycopersicum.Solyc10g055410.2.1
GWHPERDT018293
LYG028056
Jasminum.189.GWHPBFHK009403
Vitis_vinifera.VIT_204s0023g03710
GWHPFIFF021315.1
Phalaenopsis_equestris.Peq020591853.1
Dendrobium_catenatum.Dca020699985.1
GWHPFIFF017941.1
Phalaenopsis_equestris.Peq020599272.1
Dendrobium_catenatum.Dca020700906.1
GWHPFIFF016170.1
Phalaenopsis_equestris.Peq020581730.1
Dendrobium_catenatum.Dca020682484.1
Dioscorea_alata.Dioal.14G038400.1.p
Allium.g7547.t1
LregChr04G022230.mRNA1
Dioscorea_alata.Dioal.11G046900.1.p
GWHPFIFF011638.1
Phalaenopsis_equestris.Peq020585535.1
Dendrobium_catenatum.Dca020703289.1
Dioscorea_alata.Dioal.01G076800.1.p
Ananas_comosus.Aco008865.1
Musa_acuminata.GSMUA_Achr1P02420_001
Musa_acuminata.GSMUA_Achr9P24180_001
Brachypodium_distachyon.Bradi4g36460.2.p
Oryza_sativa.LOC_Os09g36730.1
Zea_mays.Zm00001d022628_P001
Zea_mays.Zm00001d006236_P002
Zea_mays.Zm00001d032024_P001
Zea_mays.Zm00001d053220_P001
Oryza_sativa.LOC_Os08g43550.1
Ananas_comosus.Aco013105.1
Gs05G068500.1
Musa_acuminata.GSMUA_Achr11P07180_001
Musa_acuminata.GSMUA_Achr1P26520_001
Musa_acuminata.GSMUA_Achr1P07550_001
Euryale_ferox.g15171.t1
Euryale_ferox.g37975.t1
Euryale_ferox.g27742.t1
Vc11G027360.1
Euryale_ferox.g34965.t1
Vc10G032130.1
Euryale_ferox.g11735.t1
Nymphaea_colorata.Nycol.I01869.1.p
Amborella_trichopoda.scaffold00055.19
Tree scale 0.1

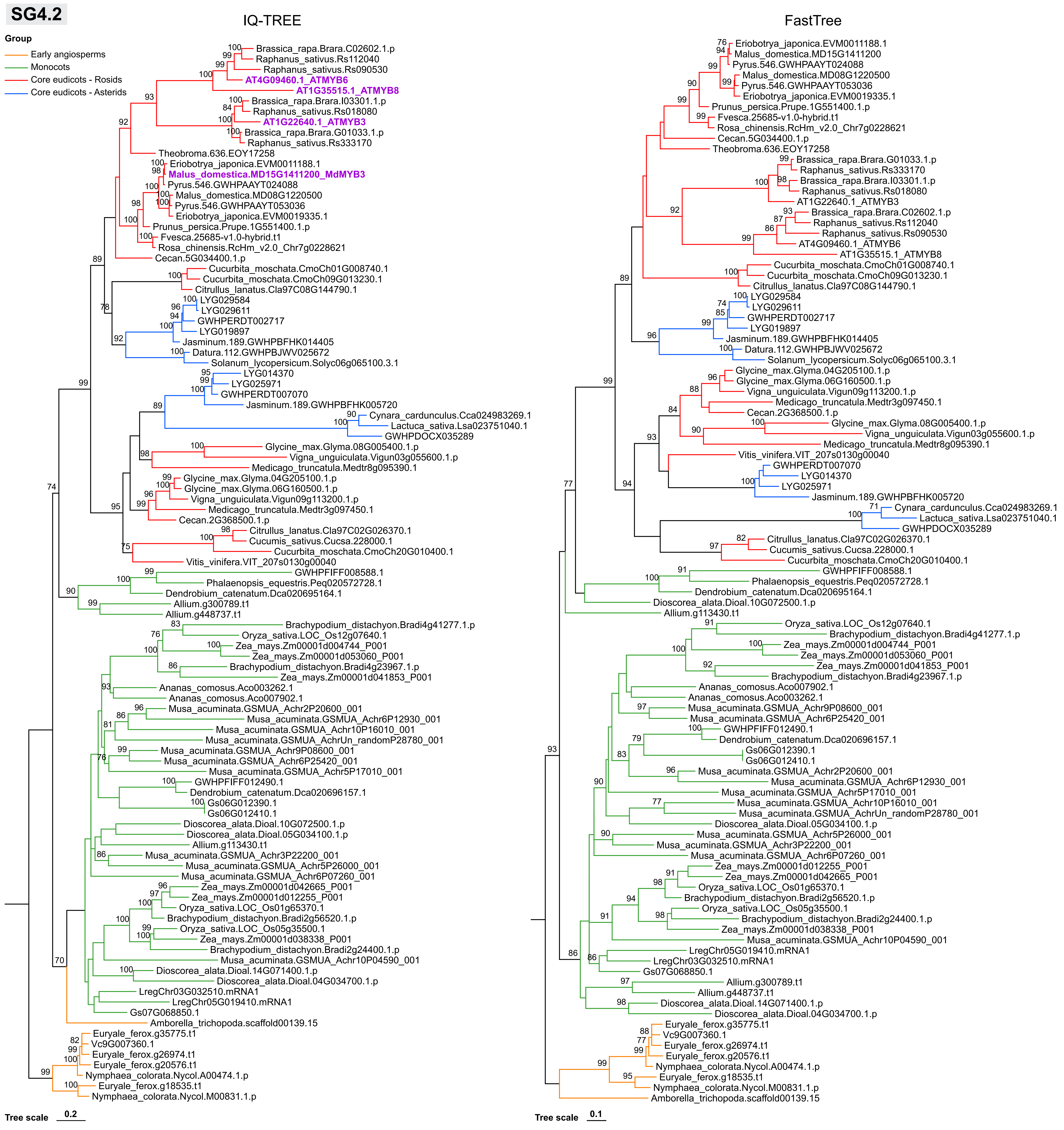

SG4.2
Group
Early angiosperms
Monocots
Core eudicots - Rosids
Core eudicots - Asterids
IQ-TREE
Brassica_rapa.Brara.C02602.1.p
Raphanus_sativus.Rs112040
Raphanus_sativus.Rs090530
AT4G09460.1_ATMYB6
AT1G35515.1_ATMYB8
Brassica_rapa.Brara.I03301.1.p
Raphanus_sativus.Rs018080
AT1G22640.1_ATMYB3
Brassica_rapa.Brara.G01033.1.p
Raphanus_sativus.Rs333170
Theobroma.636.EOY17258
Eriobotrya_japonica.EVM0011188.1
Malus_domestica.MD15G1411200_MdMYB3
Pyrus.546.GWHPAAYT024088
Malus_domestica.MD08G1220500
Pyrus.546.GWHPAAYT053036
Eriobotrya_japonica.EVM0019335.1
Prunus_persica.Prupe.1G551400.1.p
Fvesca.25685-v1.0-hybrid.t1
Rosa_chinensis.RcHm_v2.0_Chr7g0228621
Cecan.5G034400.1.p
Cucurbita_moschata.CmoCh01G008740.1
Cucurbita_moschata.CmoCh09G013230.1
Citrullus_lanatus.Cla97C08G144790.1
LYG029584
LYG029611
GWHPERDT002717
LYG019897
Jasminum.189.GWHPBFHK014405
Datura.112.GWHPBJWV025672
Solanum_lycopersicum.Solyc06g065100.3.1
LYG014370
LYG025971
GWHPERDT007070
Jasminum.189.GWHPBFHK005720
Cynara_cardunculus.Cca024983269.1
Lactuca_sativa.Lsa023751040.1
GWHPDOCX035289
Glycine_max.Glyma.08G005400.1.p
Vigna_unguiculata.Vigun03g055600.1.p
Medicago_truncatula.Medtr8g095390.1
Glycine_max.Glyma.04G205100.1.p
Glycine_max.Glyma.06G160500.1.p
Vigna_unguiculata.Vigun09g113200.1.p
Medicago_truncatula.Medtr3g097450.1
Cecan.2G368500.1.p
Citrullus_lanatus.Cla97C02G026370.1
Cucumis_sativus.Cucsa.228000.1
Cucurbita_moschata.CmoCh20G010400.1
Vitis_vinifera.VIT_207s0130g00040
GWHPFIFF008588.1
Phalaenopsis_equestris.Peq020572728.1
Dendrobium_catenatum.Dca020695164.1
Allium.g300789.t1
Allium.g448737.t1
Brachypodium_distachyon.Bradi4g41277.1.p
Oryza_sativa.LOC_Os12g07640.1
Zea_mays.Zm00001d004744_P001
Zea_mays.Zm00001d053060_P001
Brachypodium_distachyon.Bradi4g23967.1.p
Zea_mays.Zm00001d041853_P001
Ananas_comosus.Aco003262.1
Ananas_comosus.Aco007902.1
Musa_acuminata.GSMUA_Achr2P20600_001
Musa_acuminata.GSMUA_Achr6P12930_001
Musa_acuminata.GSMUA_Achr10P16010_001
Musa_acuminata.GSMUA_AchrUn_randomP28780_001
Musa_acuminata.GSMUA_Achr9P08600_001
Musa_acuminata.GSMUA_Achr6P25420_001
Musa_acuminata.GSMUA_Achr5P17010_001
GWHPFIFF012490.1
Dendrobium_catenatum.Dca020696157.1
Gs06G012390.1
Gs06G012410.1
Dioscorea_alata.Dioal.10G072500.1.p
Dioscorea_alata.Dioal.05G034100.1.p
Allium.g113430.t1
Musa_acuminata.GSMUA_Achr3P22200_001
Musa_acuminata.GSMUA_Achr5P26000_001
Musa_acuminata.GSMUA_Achr6P07260_001
Zea_mays.Zm00001d042665_P001
Zea_mays.Zm00001d012255_P001
Oryza_sativa.LOC_Os01g65370.1
Brachypodium_distachyon.Bradi2g56520.1.p
Oryza_sativa.LOC_Os05g35500.1
Zea_mays.Zm00001d038338_P001
Brachypodium_distachyon.Bradi2g24400.1.p
Musa_acuminata.GSMUA_Achr10P04590_001
Dioscorea_alata.Dioal.14G071400.1.p
Dioscorea_alata.Dioal.04G034700.1.p
LregChr03G032510.mRNA1
LregChr05G019410.mRNA1
Gs07G068850.1
Amborella_trichopoda.scaffold00139.15
Euryale_ferox.g35775.t1
Vc9G007360.1
Euryale_ferox.g26974.t1
Euryale_ferox.g20576.t1
Nymphaea_colorata.Nycol.A00474.1.p
Euryale_ferox.g18535.t1
Nymphaea_colorata.Nycol.M00831.1.p
Tree scale
0.2
FastTree
Eriobotrya_japonica.EVM0011188.1
Malus_domestica.MD15G1411200
Pyrus.546.GWHPAAYT024088
Malus_domestica.MD08G1220500
Pyrus.546.GWHPAAYT053036
Eriobotrya_japonica.EVM0019335.1
Prunus_persica.Prupe.1G551400.1.p
Fvesca.25685-v1.0-hybrid.t1
Rosa_chinensis.RcHm_v2.0_Chr7g0228621
Cecan.5G034400.1.p
Theobroma.636.EOY17258
Brassica_rapa.Brara.G01033.1.p
Raphanus_sativus.Rs333170
Brassica_rapa.Brara.I03301.1.p
Raphanus_sativus.Rs018080
AT1G22640.1_ATMYB3
Brassica_rapa.Brara.C02602.1.p
Raphanus_sativus.Rs112040
Raphanus_sativus.Rs090530
AT4G09460.1_ATMYB6
AT1G35515.1_ATMYB8
Cucurbita_moschata.CmoCh01G008740.1
Cucurbita_moschata.CmoCh09G013230.1
Citrullus_lanatus.Cla97C08G144790.1
LYG029584
LYG029611
GWHPERDT002717
LYG019897
Jasminum.189.GWHPBFHK014405
Datura.112.GWHPBJWV025672
Solanum_lycopersicum.Solyc06g065100.3.1
Glycine_max.Glyma.04G205100.1.p
Glycine_max.Glyma.06G160500.1.p
Vigna_unguiculata.Vigun09g113200.1.p
Medicago_truncatula.Medtr3g097450.1
Cecan.2G368500.1.p
Glycine_max.Glyma.08G005400.1.p
Vigna_unguiculata.Vigun03g055600.1.p
Medicago_truncatula.Medtr8g095390.1
Vitis_vinifera.VIT_207s0130g00040
GWHPERDT007070
LYG014370
LYG025971
Jasminum.189.GWHPBFHK005720
Cynara_cardunculus.Cca024983269.1
Lactuca_sativa.Lsa023751040.1
GWHPDOCX035289
Citrullus_lanatus.Cla97C02G026370.1
Cucumis_sativus.Cucsa.228000.1
Cucurbita_moschata.CmoCh20G010400.1
GWHPFIFF008588.1
Phalaenopsis_equestris.Peq020572728.1
Dendrobium_catenatum.Dca020695164.1
Dioscorea_alata.Dioal.10G072500.1.p
Allium.g113430.t1
Oryza_sativa.LOC_Os12g07640.1
Brachypodium_distachyon.Bradi4g41277.1.p
Zea_mays.Zm00001d004744_P001
Zea_mays.Zm00001d053060_P001
Zea_mays.Zm00001d041853_P001
Brachypodium_distachyon.Bradi4g23967.1.p
Ananas_comosus.Aco007902.1
Ananas_comosus.Aco003262.1
Musa_acuminata.GSMUA_Achr9P08600_001
Musa_acuminata.GSMUA_Achr6P25420_001
GWHPFIFF012490.1
Dendrobium_catenatum.Dca020696157.1
Gs06G012390.1
Gs06G012410.1
Musa_acuminata.GSMUA_Achr2P20600_001
Musa_acuminata.GSMUA_Achr6P12930_001
Musa_acuminata.GSMUA_Achr5P17010_001
Musa_acuminata.GSMUA_Achr10P16010_001
Musa_acuminata.GSMUA_AchrUn_randomP28780_001
Dioscorea_alata.Dioal.05G034100.1.p
Musa_acuminata.GSMUA_Achr5P26000_001
Musa_acuminata.GSMUA_Achr3P22200_001
Musa_acuminata.GSMUA_Achr6P07260_001
Zea_mays.Zm00001d012255_P001
Zea_mays.Zm00001d042665_P001
Oryza_sativa.LOC_Os01g65370.1
Brachypodium_distachyon.Bradi2g56520.1.p
Oryza_sativa.LOC_Os05g35500.1
Brachypodium_distachyon.Bradi2g24400.1.p
Zea_mays.Zm00001d038338_P001
Musa_acuminata.GSMUA_Achr10P04590_001
LregChr05G019410.mRNA1
LregChr03G032510.mRNA1
Gs07G068850.1
Allium.g300789.t1
Allium.g448737.t1
Dioscorea_alata.Dioal.14G071400.1.p
Dioscorea_alata.Dioal.04G034700.1.p
Euryale_ferox.g35775.t1
Vc9G007360.1
Euryale_ferox.g26974.t1
Euryale_ferox.g20576.t1
Nymphaea_colorata.Nycol.A00474.1.p
Euryale_ferox.g18535.t1
Nymphaea_colorata.Nycol.M00831.1.p
Amborella_trichopoda.scaffold00139.15
Tree scale
0.1

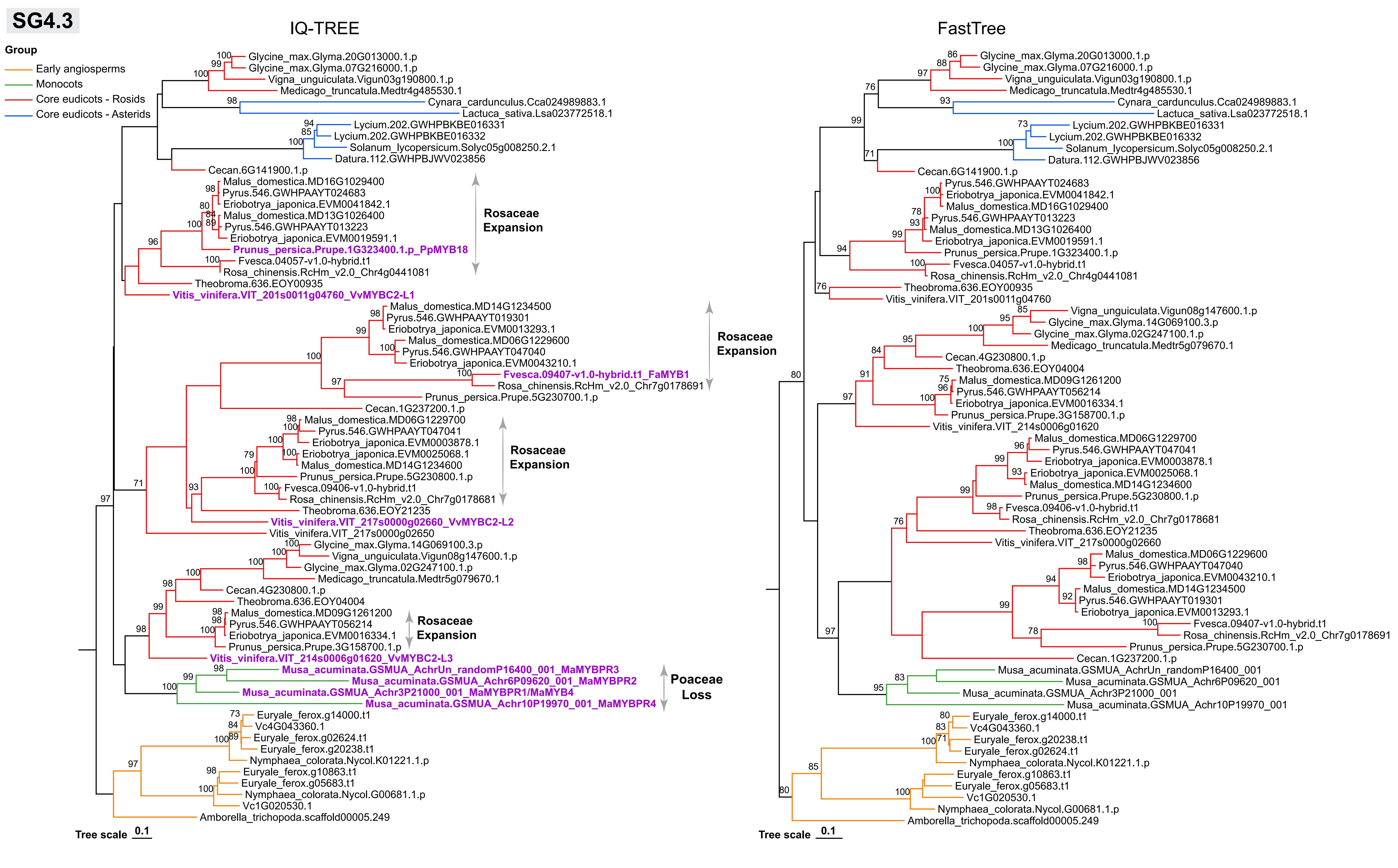
SG4.3
IQ-TREE
FastTree
Group
Early angiosperms
Monocots
Core eudicots - Rosids
Core eudicots - Asterids
Glycine_max.Glyma.20G013000.1.p
Glycine_max.Glyma.07G216000.1.p
Vigna_unguiculata.Vigun03g190800.1.p
Medicago_truncatula.Medtr4g485530.1
Cynara_cardunculus.Cca024989883.1
Lactuca_sativa.Lsa023772518.1
Lycium.202.GWHPBKBE016331
Lycium.202.GWHPBKBE016332
Solanum_lycopersicum.Solyc05g008250.2.1
Datura.112.GWHPBJWV023856
Cecan.6G141900.1.p
Malus_domestica.MD16G1029400
Pyrus.546.GWHPAAYT024683
Eriobotrya_japonica.EVM0041842.1
Malus_domestica.MD13G1026400
Pyrus.546.GWHPAAYT013223
Eriobotrya_japonica.EVM0019591.1
Prunus_persica.Prupe.1G323400.1.p_PpMYB18
Fvesca.04057-v1.0-hybrid.t1
Rosa_chinensis.RcHm_v2.0_Chr4g0441081
Theobroma.636.EOY00935
Vitis_vinifera.VIT_201s0011g04760_VvMYBC2-L1
Rosaceae Expansion
Malus_domestica.MD14G1234500
Pyrus.546.GWHPAAYT019301
Eriobotrya_japonica.EVM0013293.1
Malus_domestica.MD06G1229600
Pyrus.546.GWHPAAYT047040
Eriobotrya_japonica.EVM0043210.1
Fvesca.09407-v1.0-hybrid.t1_FaMYB1
Rosa_chinensis.RcHm_v2.0_Chr7g0178691
Prunus_persica.Prupe.5G230700.1.p
Cecan.1G237200.1.p
Rosaceae Expansion
Malus_domestica.MD06G1229700
Pyrus.546.GWHPAAYT047041
Eriobotrya_japonica.EVM0003878.1
Eriobotrya_japonica.EVM0025068.1
Malus_domestica.MD14G1234600
Prunus_persica.Prupe.5G230800.1.p
Fvesca.09406-v1.0-hybrid.t1
Rosa_chinensis.RcHm_v2.0_Chr7g0178681
Theobroma.636.EOY21235
Vitis_vinifera.VIT_217s0000g02660_VvMYBC2-L2
Vitis_vinifera.VIT_217s0000g02650
Rosaceae Expansion
Glycine_max.Glyma.14G069100.3.p
Vigna_unguiculata.Vigun08g147600.1.p
Glycine_max.Glyma.02G247100.1.p
Medicago_truncatula.Medtr5g079670.1
Cecan.4G230800.1.p
Theobroma.636.EOY04004
Malus_domestica.MD09G1261200
Pyrus.546.GWHPAAYT056214
Eriobotrya_japonica.EVM0016334.1
Prunus_persica.Prupe.3G158700.1.p
Vitis_vinifera.VIT_214s0006g01620_VvMYBC2-L3
Rosaceae Expansion
Musa_acuminata.GSMUA_AchrUn_randomP16400_001_MaMYBPR3
Musa_acuminata.GSMUA_Achr6P09620_001_MaMYBPR2
Musa_acuminata.GSMUA_Achr3P21000_001_MaMYBPR1/MaMYB4
Musa_acuminata.GSMUA_Achr10P19970_001_MaMYBPR4
Poaceae Loss
Euryale_ferox.g14000.t1
Vc4G043360.1
Euryale_ferox.g02624.t1
Euryale_ferox.g20238.t1
Nymphaea_colorata.Nycol.K01221.1.p
Euryale_ferox.g10863.t1
Euryale_ferox.g05683.t1
Nymphaea_colorata.Nycol.G00681.1.p
Vc1G020530.1
Amborella_trichopoda.scaffold00005.249
Tree scale 0.1
Glycine_max.Glyma.20G013000.1.p
Glycine_max.Glyma.07G216000.1.p
Vigna_unguiculata.Vigun03g190800.1.p
Medicago_truncatula.Medtr4g485530.1
Cynara_cardunculus.Cca024989883.1
Lactuca_sativa.Lsa023772518.1
Lycium.202.GWHPBKBE016331
Lycium.202.GWHPBKBE016332
Solanum_lycopersicum.Solyc05g008250.2.1
Datura.112.GWHPBJWV023856
Cecan.6G141900.1.p
Pyrus.546.GWHPAAYT024683
Eriobotrya_japonica.EVM0041842.1
Malus_domestica.MD16G1029400
Pyrus.546.GWHPAAYT013223
Malus_domestica.MD13G1026400
Eriobotrya_japonica.EVM0019591.1
Prunus_persica.Prupe.1G323400.1.p
Fvesca.04057-v1.0-hybrid.t1
Rosa_chinensis.RcHm_v2.0_Chr4g0441081
Theobroma.636.EOY00935
Vitis_vinifera.VIT_201s0011g04760
Vigna_unguiculata.Vigun08g147600.1.p
Glycine_max.Glyma.14G069100.3.p
Glycine_max.Glyma.02G247100.1.p
Medicago_truncatula.Medtr5g079670.1
Cecan.4G230800.1.p
Theobroma.636.EOY04004
Malus_domestica.MD09G1261200
Pyrus.546.GWHPAAYT056214
Eriobotrya_japonica.EVM0016334.1
Prunus_persica.Prupe.3G158700.1.p
Vitis_vinifera.VIT_214s0006g01620
Malus_domestica.MD06G1229700
Pyrus.546.GWHPAAYT047041
Eriobotrya_japonica.EVM0003878.1
Eriobotrya_japonica.EVM0025068.1
Malus_domestica.MD14G1234600
Prunus_persica.Prupe.5G230800.1.p
Fvesca.09406-v1.0-hybrid.t1
Rosa_chinensis.RcHm_v2.0_Chr7g0178681
Theobroma.636.EOY21235
Vitis_vinifera.VIT_217s0000g02660
Malus_domestica.MD06G1229600
Pyrus.546.GWHPAAYT047040
Eriobotrya_japonica.EVM0043210.1
Malus_domestica.MD14G1234500
Pyrus.546.GWHPAAYT019301
Eriobotrya_japonica.EVM0013293.1
Fvesca.09407-v1.0-hybrid.t1
Rosa_chinensis.RcHm_v2.0_Chr7g0178691
Prunus_persica.Prupe.5G230700.1.p
Cecan.1G237200.1.p
Musa_acuminata.GSMUA_AchrUn_randomP16400_001
Musa_acuminata.GSMUA_Achr6P09620_001
Musa_acuminata.GSMUA_Achr3P21000_001
Musa_acuminata.GSMUA_Achr10P19970_001
Euryale_ferox.g14000.t1
Vc4G043360.1
Euryale_ferox.g20238.t1
Euryale_ferox.g02624.t1
Nymphaea_colorata.Nycol.K01221.1.p
Euryale_ferox.g10863.t1
Euryale_ferox.g05683.t1
Vc1G020530.1
Nymphaea_colorata.Nycol.G00681.1.p
Amborella_trichopoda.scaffold00005.249
Tree scale 0.1

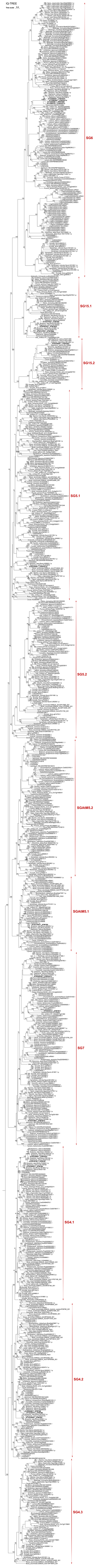
IQ-TREE
Tree scale: 0.1
SG6
SG15.1
SG15.2
SG5.1
SG5.2
SGAtM5.2
SGAtM5.1
SG7
SG4.1
SG4.2
SG4.3

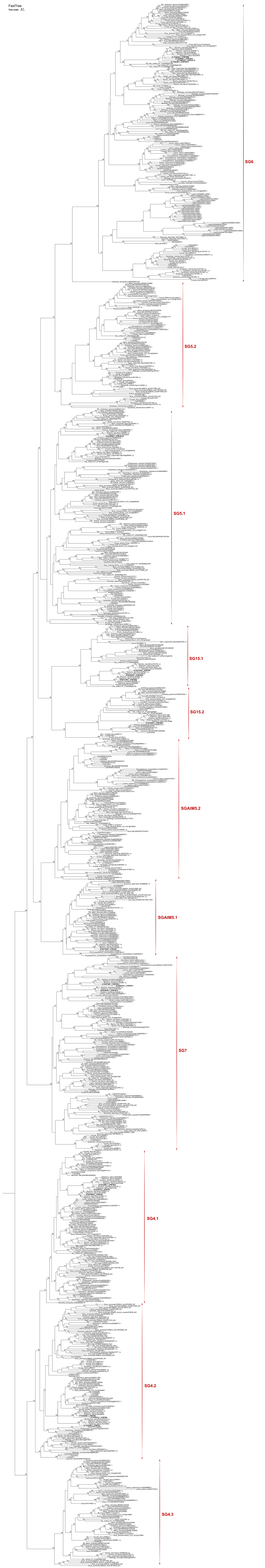
FastTree
Tree scale: 0.1
SG6
SG5.2
SG5.1
SG15.1
SG15.2
SGAtM5.2
SGAtM5.1
SG7
SG4.1
SG4.2
SG4.3

Fig. S3 ML phylogenetic trees of key flavonoid-regulatory R2R3-MYB subgroups across 44 horticultural species. Bootstrap values are displayed on the branches. Different plant lineages are distinguished by distinct branch colors. Functionally validated genes (as referenced in Fig. 2) are highlighted in purple. Inferred gene expansion and loss events are annotated to the right of each tree. (a) SG6; (b) SG5.1; (c) SG5.2; (d) SG15.1/SG15.2; (e) SG7; (f) SGAtM5.1; (g) SGAtM5.2; (h) SG4.1; (i) SG4.2; (j) SG4.3; (k) Comprehensive tree of all key flavonoid-regulatory subgroups constructed using IQ-TREE; (l) Comprehensive tree of all key flavonoid-regulatory subgroups constructed using FastTree.
